# Analysis of Ribosomal Protein Gene Structures: Implications for Intron Evolution 

**DOI:** 10.1371/journal.pgen.0020025

**Published:** 2006-03-03

**Authors:** Maki Yoshihama, Akihiro Nakao, Hung D Nguyen, Naoya Kenmochi

**Affiliations:** Frontier Science Research Center, University of Miyazaki, Kiyotake, Miyazaki, Japan; National Institute of Genetics, Japan

## Abstract

Many spliceosomal introns exist in the eukaryotic nuclear genome. Despite much research, the evolution of spliceosomal introns remains poorly understood. In this paper, we tried to gain insights into intron evolution from a novel perspective by comparing the gene structures of cytoplasmic ribosomal proteins (CRPs) and mitochondrial ribosomal proteins (MRPs), which are held to be of archaeal and bacterial origin, respectively. We analyzed 25 homologous pairs of CRP and MRP genes that together had a total of 527 intron positions. We found that all 12 of the intron positions shared by CRP and MRP genes resulted from parallel intron gains and none could be considered to be “conserved,” i.e., descendants of the same ancestor. This was supported further by the high frequency of proto-splice sites at these shared positions; proto-splice sites are proposed to be sites for intron insertion. Although we could not definitively disprove that spliceosomal introns were already present in the last universal common ancestor, our results lend more support to the idea that introns were gained late. At least, our results show that MRP genes were intronless at the time of endosymbiosis. The parallel intron gains between CRP and MRP genes accounted for 2.3% of total intron positions, which should provide a reliable estimate for future inferences of intron evolution.

## Synopsis

Genes in eukaryotes are usually intervened by extra bits of DNA sequence, called introns, that have to be removed after the genes are transcribed into RNA. Why do introns exist in eukaryotic genes? What is the reason for the increased intron density in higher eukaryotes? There is much that is not known about introns. This research tries to clarify the evolutionary process by which introns arose by comparing the gene structures of two types of ribosomal proteins; one in cytoplasm and the other in mitochondria of the cell. Since cytoplasm and mitochondria are of archaeal and bacterial origin, respectively, cytoplasmic ribosomal proteins (CRPs) and mitochondrial ribosomal proteins (MRPs) are believed to diverge at the same time with the divergence of archaea and bacteria. Thus, a comparative analysis of CRP and MRP genes may reveal whether introns already existed at the last common ancestor of archaea and bacteria (introns-early) or whether they emerged late (introns-late). The results make it clear, at least, that all of the introns in MRP genes were gained during the course of eukaryotic evolution and therefore lend more support to the introns-late theory.

## Introduction

There are two opposing theories regarding when spliceosomal introns originated. The introns-early theory proposes that primordial spliceosomal introns already existed at the beginning of life [1–3]. The earliest version of the introns-early theory postulated that, during the course of evolution, introns were completely lost from prokaryotes while being partly retained in eukaryotes. A recently revised version suggests that although intron loss was the main driving force for intron evolution, some modern introns were inserted recently [4,5]. The introns-late theory, on the other hand, holds that all spliceosomal introns were only recently inserted into eukaryotic genes [6,7]; although the introns-late theory allows for some intron losses, it stresses that intron gains played the primary role in forming the modern pattern of introns. The debate between these two theories remains vigorous [4,8].

Intron insertions have been proposed to occur primarily in “proto-splice sites” with the consensus sequence MAG|R, where M is A or C, R is A or G, and the vertical line represents the intron insertion site [9]. However, the distribution of intron phase predicted from the distribution of proto-splice sites did not account for a set of observed data [10]. (Phase 0, 1, and 2 introns are defined as introns inserted before the first, after the first, and after the second nucleotide of a codon, respectively.) Therefore, more evidence is needed to test the proto-splice site hypothesis.

About 25% to 30% of intron positions are shared between species that diverged in the distant past, such as plants and humans [11,12]. Some of these shared positions may be the result of conservation of intron position, and some may be due to parallel gains. Although parallel gains are known to have occurred [13], it is unclear what percentage of intron positions they account for. Many groups have inferred the evolution of spliceosomal introns without taking into consideration the proportion of parallel gains [14,15]; however, Qiu et al. [8] found that most intron positions shared between distantly diverged kingdoms were due to parallel gains. Recently, Sverdlov et al. [16] estimated that parallel gains accounted for 5%–10% of intron positions shared between homologs. Therefore, determination of the correct proportion of parallel gains is essential for determining the history of intron evolution.

The ribosome is a vital component of the translational machinery and therefore of all cellular life. Consequently, ribosomal proteins (RPs) have been highly conserved throughout evolution [17,18]. Thus, it is possible to compare RPs across a wide range of distantly diverged species [19,20]. Mitochondrial ribosomes are considered to be of bacterial origin (that is, they are a product of endosymbiosis), as evidenced by the considerable homology that exists between mitochondrial ribosomal proteins (MRPs) and bacterial RPs [21,22]. Cytoplasmic ribosomal proteins (CRPs), on the other hand, are thought to have evolved independently from archaea, although there is sufficient homology between MRPs and CRPs to allow a comparison of their gene structures. Like most mitochondrial genes, MRP genes were transferred to the nuclear genome after endosymbiosis [23] and, like their cellular counterparts, contain spliceosomal introns. Thus, by comparing the intron/exon structures of MRP and CRP genes, it may be possible to determine whether spliceosomal introns existed in their last common ancestor. If at least one clear case of intron position conservation is found (i.e., introns at this position are descendants of the same ancestral intron), then it can be concluded that spliceosomal introns existed in the last common ancestor of CRP and MRP genes (introns-early). Otherwise, there are two possibilities. The first is that spliceosomal introns already existed in the last common ancestor of CRP and MRP genes but were mostly (if not completely) lost along the bacterial lineage before endosymbiosis due to the high pressure for genome reduction [24,25]. The second possibility is that spliceosomal introns arose after endosymbiosis (introns-late). For both of these possible scenarios, any intron positions shared between CRP and MRP genes are likely to be the result of parallel gains, allowing the proportion of introns obtained through parallel gains in two distantly diverged lineages to be determined.

## Results

### Comparison of Intron Positions

**Figure 1:**
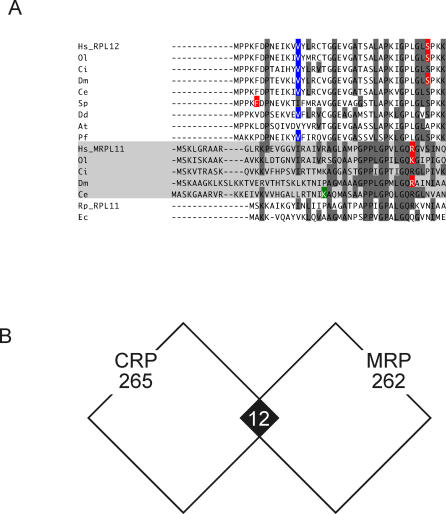
Comparison of Intron Positions Shared by CRP and MRP Genes (A) A part of the sequence alignment of nine *RPL12* genes (top), five *MRPL11* genes (light grey shading), and two prokaryotic *RPL11* genes (bottom). Conserved amino acids are highlighted by a dark grey background. Phase 0, 1, and 2 introns are highlighted by red, blue, and green backgrounds, respectively. (B) A Venn diagram of overlap showing that 12 intron positions are shared by CRP and MRP genes. CRP and MRP genes have 265 and 262 intron positions, respectively. At, *A. thaliana;* Ce, *Ca. elegans;* Ci, *Ci. intestinalis;* Dd, *Di. discoideum;* Dm, *Dr. melanogaster;* Ec, *E. coli;* Hs, *H. sapiens;* Ol, *O. laptides;* Pf, *P. falciparum;* Rp, *R. prowazekii;* Sp, S. pombe.

A total of 79 MRP genes were found in the human genome [21,22]. Of these, 43 were homologous to bacterial genes, and among these 43, 25 were homologous to eukaryotic CRP genes. We compared the gene structures of these 25 homologous pairs. In particular, the gene structures of CRPs from nine eukaryotes *(Homo sapiens, Oryzias laptides, Ciona intestinalis, Drosophila melanogaster, Caenorhabditis elegans, Schizosaccharomyces pombe, Dictyostelium discoideum, Arabidopsis thaliana,* and *Plasmodium falciparum)* were compared with the gene structures of MRPs from five eukaryotes *(H. sapiens, O. laptides, Ci. intestinalis, Dr. melanogaster,* and *Ca. elegans)* (Table S1). In order to make the multiple alignments between CRP and MRP genes more reliable, RP genes of two bacterial species *(Rickettsia prowazekii* and *Escherichia coli)* were also included in the analyses (Figure 1A, Figure S1, and Dataset S1). We found 570 introns (265 intron positions) in the coding regions of the CRP genes and 423 introns (262 intron positions) in the coding regions of the MRP genes. The multiple sequence alignments showed that, out of the total of 527 intron positions, only 12 (2.3%) were shared by CRP and MRP genes; i.e., an intron was present at each of these positions in at least one CRP gene and in at least one MRP gene (Figure 1B, Figure S1, and Dataset S1). We classified these 12 positions according to their level of conservation: Three positions were in highly conserved regions, six positions were in regions of moderate homology, and the remaining three positions were of low homology and therefore had a high probability of being misaligned. The three low-homology positions were excluded, which left nine positions for further analysis.

### Parsimony Analysis of CRP–MRP Shared Positions

The maximum parsimony method was used to infer the most parsimonious scenarios of these nine shared positions. We recently proposed a maximum likelihood approach for inferring the evolution of introns [26]; however, we believe that maximum parsimony is the best choice for the current dataset because maximum likelihood uses only patterns of intron position in the conserved regions of the multiple sequence alignment, and our dataset is not large enough to make valid statistical inferences using this method.

**Figure 2:**
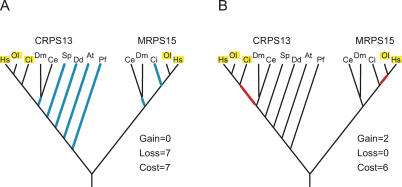
Parsimony Analysis of the *CRPS13*/*MRPS15* Shared Position (*K* = 100) The phylogenetic distribution of the intron position shared between the *CRPS13* and *MRPS15* genes is shown. Branches that are predicted to have an intron gain or loss are shown in red and blue, respectively. Organisms that currently possess an intron are shown in yellow. The most parsimonious scenario for the case of parallel intron gain was favored because the cost was smaller. (A) The parsimonious scenario of intron evolution for the case of intron position conservation. The ancestral intron was lost on five branches leading to *CRPS13* genes *(P. falciparum, A. thaliana, Di. discoideum, S. pombe,* and the ancestral branch of *Ca. elegans* and *Dr. melanogaster)* and on two branches leading to *MRP15* genes (the ancestral branch of *Ca. elegans* and *Dr. melanogaste*r and the terminal branch to *Ci. intestinalis*). (B) The most parsimonious scenario of intron evolution for the case of parallel intron gain. An intron was gained on the ancestral branch of *Ci. intestinalis, O. laptides,* and H. sapiens in the CRP lineage, and another intron was gained on the ancestral branch of O. laptides and H. sapiens in the MRP lineage. The tree is based on data from Hedges [31]. At, *A. thaliana;* Ce, *Ca. elegans;* Ci, *Ci. intestinalis;* Dd, *Di. discoideum;* Dm, *Dr. melanogaster;* Hs, *H. sapiens;* Ol, *O. laptides;* Pf, *P. falciparum;* Sp, S. pombe.

The costs of the most parsimonious scenario of the two possible cases, intron position conservation and parallel intron gain, were inferred for each shared intron position (Figure 2 and Table 1). For the case *K* = 1 (see Materials and Methods for the definition of *K*), all nine of these shared intron positions were predicted to be the result of parallel gains. Even when intron loss was presumed to occur 100 times more easily than intron gain (*K* = 100), seven out of nine positions were still classified as being the result of parallel gains, whereas only one position (the one shared between the *RPL7* gene of A. thaliana and the three *MRPL30* genes of *H. sapiens, O. laptides,* and *Ci. intestinalis*) was classified as being the result of position conservation. (The ninth position shared between *RPS23–2* and *MRPS12–2* genes could not be classified because the costs of each scenario were equal.) However, considering that A. thaliana has a very high rate of intron gain [12,14,26], the possibility that this intron position was also the result of parallel gain is high. Taking all of the results together, we concluded that all 12 intron positions (including the three misaligned positions) shared between CRP and MRP were the result of parallel gains. Parallel gains therefore account for 2.3% of all intron positions, 4.5% of CRP intron positions, and 4.6% of MRP intron positions.

**Table 1:**
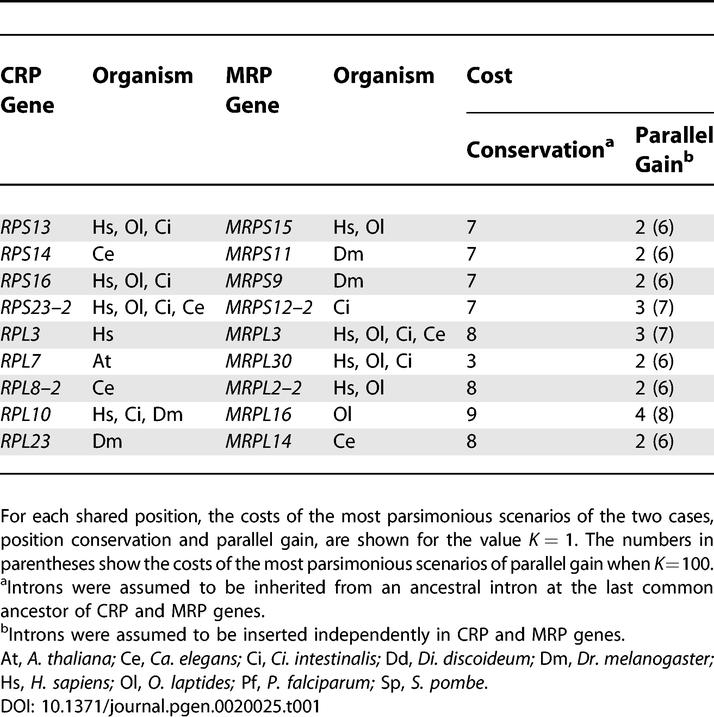
Parsimony Analysis of Intron Positions Shared by CRP and MRP Genes

### Proto-Splice Site Tendency

**Figure 3:**
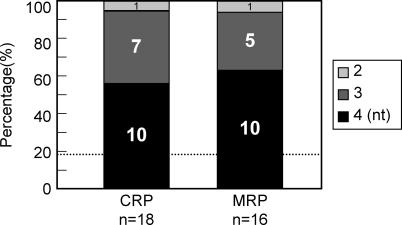
Proto-Splice Site Tendency The percentage of nucleotides that were identical to the proto-splice site sequence was calculated using 18 CRP splice sites and 16 MRP splice sites in nine shared intron positions. For example, ten (63%) of the splice sites in MRP genes have four-nucleotide (nt) matches with the proto-splice site (MAG|R). The dotted line shows the estimated average in the whole genomes of seven species.

In order to analyze the nucleotide sequences surrounding the nine shared positions, multiple alignments of these sequences were generated (Figure S2), and the proto-splice site tendency of each position was investigated (Figure 3). Fifty-six percent of splice sites in CRP genes were completely consistent with the proto-splice site MAG|R. For MRP genes the percentage was even higher (63%). The average distribution of proto-splice sites at all intron positions was 29% among 222 CRP genes from nine species, and 27% among 120 MRP genes from five species**.** The estimated average distribution of proto-splice sites at all intron positions in the whole genomes of seven of the nine species, excluding O. laptides and *Ci. intestinalis,* was 19% (unpublished data). The frequencies of proto-splice sites in the coding regions of 25 CRP genes (14,847 bp) and 25 MRP genes (17,490 bp) were 3.3% and 2.9%, respectively. The strong tendency to have proto-splice sites at intron positions shared by CRP and MRP genes supports the proto-splice site hypothesis and further supports the above conclusion that these nine shared intron positions are the result of parallel gains. The higher-than-average number of proto-splice sites at intron positions in CRP and MRP genes than in the complete genomes can be explained by the higher degree of conservation of CRP and MRP genes than of other genes.

## Discussion

We compared intron positions in genes of MRP origin with those in genes of CRP origin to gain insight into the evolution of spliceosomal introns. Since MRP and CRP genes are of bacterial and archaeal origin, respectively, this comparison was expected to shed new light on this topic. A similar analysis using genes of organelle origin was reported previously, but this generated extensive discussion and, due to the limited numbers of intron positions and species used, no definitive conclusion was reached [27–29].

**Figure 4:**
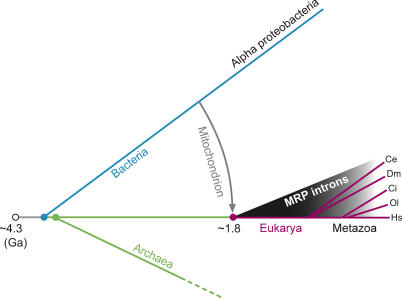
Predicted Time of Appearance of MRP Introns MRP genes were predicted to be intronless at the time of endosymbiosis (arrow) and all of their spliceosomal introns were gained after their transposition to the nuclear genome. The time scale is based on data from Hedges [31] and Battistuzzi et al. [32]. Ce, *Ca. elegans;* Ci, *Ci. intestinalis;* Dm, *Dr. melanogaster;* Ga, billion years ago; Hs, *H. sapiens;* Ol, *O. laptides*.

In this study, no clear case of intron position conservation between CRP and MRP genes was found. Most (if not all) MRP introns appear to have been inserted after endosymbiosis (Figure 4). We believe these results indicate the possibility that spliceosomal introns did not exist in the last common ancestor of CRP and MRP genes. There are mainly two reasons for this. First, if spliceosomal introns did exist at that time, these introns must have been completely lost before endosymbiosis in the bacterial lineage from which mitochondria originated. However, because spliceosomal introns have completely disappeared in all of the present archaeal and bacterial species without leaving any trace, it is very unlikely that this occurred. Second, the intron patterns of CRP genes and MRP genes are quite similar: They have roughly the same number of intron positions and the same ratio of shared intron positions. It is therefore reasonable to assume that rates of intron gain and loss are roughly the same in both lineages. If this is the case, introns in CRP genes would also mostly have been gained after endosymbiosis. This is inconsistent with the introns-early theory, which postulates that intron loss is the main driving force for intron evolution. Consequently, we believe that our results are better explained by the introns-late theory.

Our results show that parallel gains between CRP and MRP genes account for 2.3% of the total intron positions. A recent simulation estimated that parallel intron gains account for 5%–10% of shared intron positions (1.3%–3.0% of total intron positions) [16], consistent with our result. Their method, however, was based on the still-debated proto-splice site hypothesis and assumptions about the target site frequency. In contrast, our estimate was derived from observed data and therefore should be more reliable.

It is known that spliceosomal introns existed in quite high density in the last common ancestor of the three eukaryotic kingdoms: animals, fungi, and plants [11,12,26]. However, the evolution of spliceosomal introns at the earliest stage of eukaryotic evolution remains unclear. This research has shown that, at the least, mitochondrial genes were intronless at the time that eukaryotes emerged, and the intron positions shared between CRP and MRP genes have resulted from parallel gains. Consequently, future inferences made about intron evolution in eukaryotes should take the contribution of parallel gains into account.

## Materials and Methods

### Datasets.

The CRP genes of six eukaryotes: *H. sapiens, Ci. intestinalis, Dr. melanogaster, Ca. elegans, S. pombe,* and *P. falciparum,* together with the RP genes of E. coli were taken from the manually curated Ribosomal Protein Gene database (RPG; http://ribosome.med.miyazaki-u.ac.jp). The CRP gene sequences of O. laptides were collected from Medaka UTGB (http://medaka.utgenome.org) by performing a BLAST search using human CRP genes as queries. The CRP gene sequences of A. thaliana and *Di. discoideum* were first collected from the Arabidopsis Information Resource (TAIR; http://www.arabidopsis.org) and dictyBase (http://www.dictybase.org), respectively, by using annotation, and were then confirmed by aligning their sequences with those of other species. The RP genes of R. prowazekii were taken from the National Center for Biotechnology Information (NCBI; http://www.ncbi.nlm.nih.gov) using annotation. Human MRP genes were taken from the HUGO Gene Nomenclature Committee (HGNC) Gene Family Nomenclature's Mitochondria Ribosomal Proteins homepage (http://www.gene.ucl.ac.uk/nomenclature/genefamily/MRPs.html). The MRP genes for other species were selected in a way similar to that used for the CRP genes of A. thaliana. If there was no annotation, sequences were collected by performing a BLAST search using sequences from humans and other species. Genome homepages for the different species we investigated are as follows: *Ci. intestinalis* (Joint Genome Institute [JGI]; http://genome.jgi-psf.org/ciona4/ciona4.home.html); *Dr. melanogaster* (FlyBase; http://flybase.net); *Ca. elegans* (WormBase; http://www.wormbase.org); S. pombe (http://www.sanger.ac.uk/Projects/S_pombe); and P. falciparum (http://www.sanger.ac.uk/Projects/P_falciparum). When a gene existed in multiple copies, the copy with the most introns was used in the analysis. The sequences of the 25 pairs of CRP/MRP genes are available at http://ribosome.miyazaki-med.ac.jp/crpmrp/view.cgi.

### Construction of the intron matrix and classification of homology.

ClustalW (1.82) [30] was used to align sequences of homologous genes. An ad hoc program was written in the C programming language to automatically mark intron positions and sequence similarities in the output alignments of ClustalW (Figure S1). Introns that appeared at exactly the same position in at least two homologous genes were counted as shared introns. For similarity marking, we divided the investigated species into three groups: group 1 had nine species possessing CRP genes, group 2 had five species possessing MRP genes, and group 3 had two bacterial species. An amino acid was considered to be conserved if it appeared in at least four species and two groups. The conservation of an exon sequence flanking an intron position was classified at three levels according to the number of conserved amino acids in the nine–amino acid window surrounding the intron position: high conservation, from seven to nine conserved amino acids; moderate conservation, from four to six conserved amino acids; and low conservation, fewer than four conserved amino acids.

### Maximum parsimony method.

Our maximum parsimony method, which is based on the assumption that intron gain and loss events occur rarely in evolution, accepted the scenario that had the least cost as measured by a function of gains and losses. Assume that the probabilities of intron gain and loss are constant across branches and are much smaller than the probabilities of unchanging intron state; then the probability of occurrence of an intron evolution scenario will grow proportionally with *α^gains^* × *β^losses^,* where *α* and *β* are the probabilities of gain and loss on each branch, respectively, and *gains* and *losses* are the number of gain and loss events, respectively. A scenario with higher probability of occurrence will be preferred, so the following cost function is used:


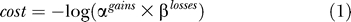


Notice that the relative difference between the costs of two scenarios does not depend much on the particular values of *α* or *β* but depends largely on the ratio *K* = *β*/*α,* which means intron loss occurs *K* times more easily than gain. Therefore, we chose to fix the value of *β* to 0.1. With this simplification, the cost function becomes:


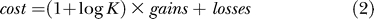


Two different values of *K* (*K* = 1 and *K* = 100) were used. Unlike the method of Rogozin et al. [12] which counts an intron at the root node as a gain event, our method takes the ancestral state “as a given.” An ad hoc program was written in the C language to automatically perform the calculation.

## Supporting Information

Dataset S1 Matrix of the Entire Set of Intron Presences and Absences (8 KB TXT)

Figure S1 Alignments of Amino Acid Sequences and Intron Positions MRP homologs are indicated with light grey shading. Conserved amino acids are highlighted by a dark grey background. Phase 0, 1, and 2 intron positions are highlighted by red, blue, and green backgrounds, respectively.
At, *A. thaliana;* Ce, *Ca. elegans;* Ci, *Ci. intestinalis;* Dd, *Di. discoideum;* Dm, *Dr. melanogaster;* Ec, *E. coli;* Hs, *H. sapiens;* Ol, *O. laptides;* Pf, *P. falciparum;* Rp, *R. prowazekii;* Sp, S. pombe .
(243 KB PDF)

Figure S2 Alignments of 20-bp Sequences Flanking the Intron Positions Shared by the Genes Encoding CRPs and MRPs Splice sites that are completely consistent with the MAG|R sequence are shown in red. Conserved nucleotides between CRP and MRP genes are highlighted by a grey background. + indicates the presence of intron.
At, *A. thaliana;* Ce, *Ca. elegans;* Ci, *Ci. intestinalis;* Dd, *Di. discoideum;* Dm, *Dr. melanogaster;* Hs, *H. sapiens;* Ol, *O. laptides;* Pf, *P. falciparum;* Sp, S. pombe.
(47 KB PDF)

Table S1 List of CRP and MRP Homologs *For O. laptides and *Ci. intestinalis,* the scaffold number was shown. For *Di. discoideum,* the dictyBaseID was shown.
†For genes with multiple copies, only one copy was used. When the number of introns in multiple copies of the same gene was different, the one with the most introns was used.
‡Gene structures were determined by a combination of these sequences.
§Gene sequences were partial.
At, *A. thaliana;* Ce, *Ca. elegans;* Ci, *Ci. intestinalis;* Dd, *Di. discoideum;* Dm, *Dr. melanogaster;* Hs, *H. sapiens;* Ol, *O. laptides;* Pf, *P. falciparum;* Sp, S. pombe.
(59 KB PDF)

## Abbreviations

CRP: cytoplasmic ribosomal protein
MRP: mitochondrial ribosomal protein
RP: ribosomal protein
