# Supplementary material for: Analysis of Ribosomal Protein Gene Structures: Implications for Intron Evolution
Source: PLoS Genet. 2006 Mar 3;2(3):e25. doi: 10.1371/journal.pgen.0020025 (PMC1386722; doi:10.1371/journal.pgen.0020025)
Supplement: Supplementary file 1 [file pgen.0020025.sg001.pdf]

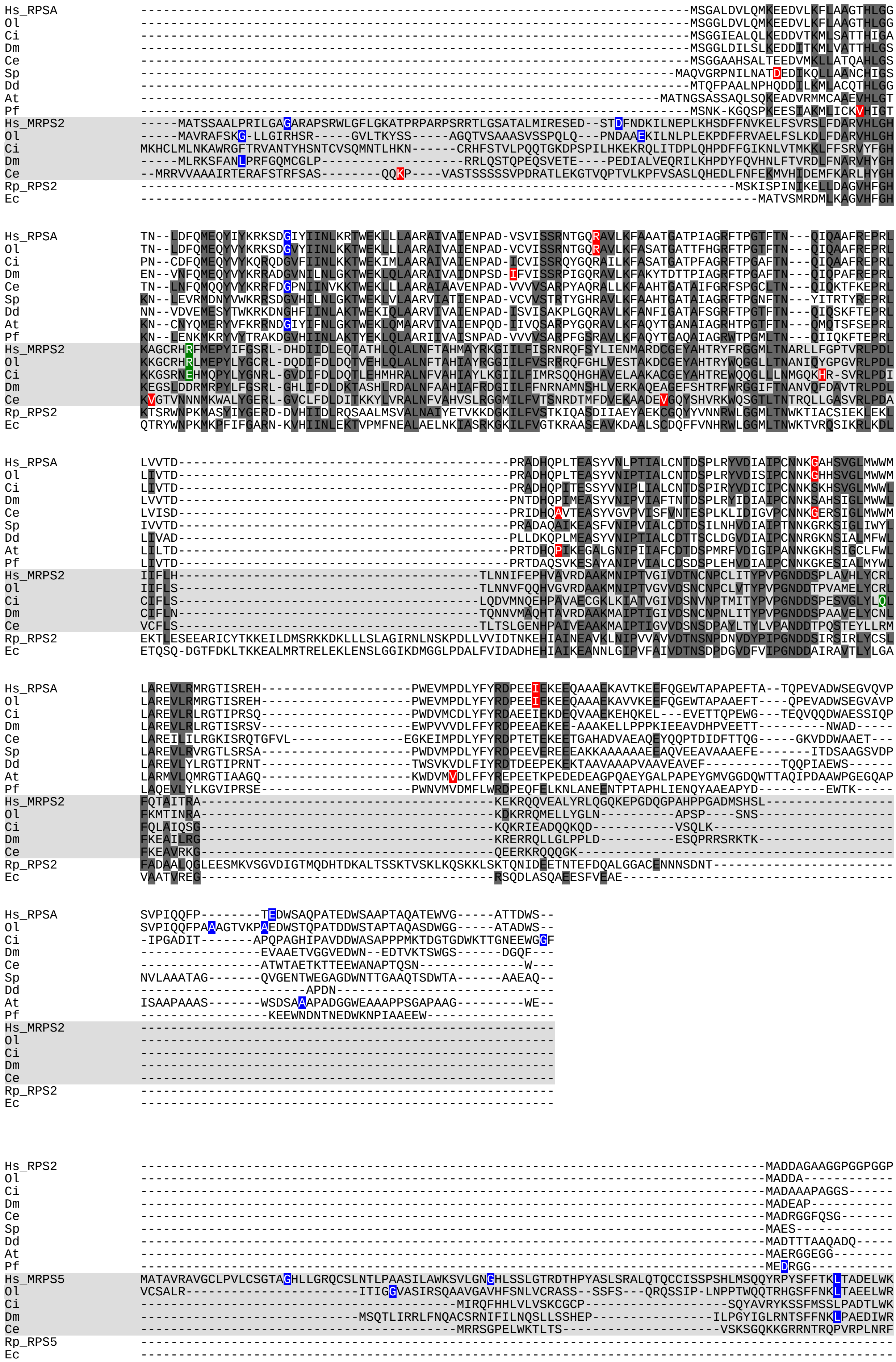

Figure S1. Yoshihama *et al.*

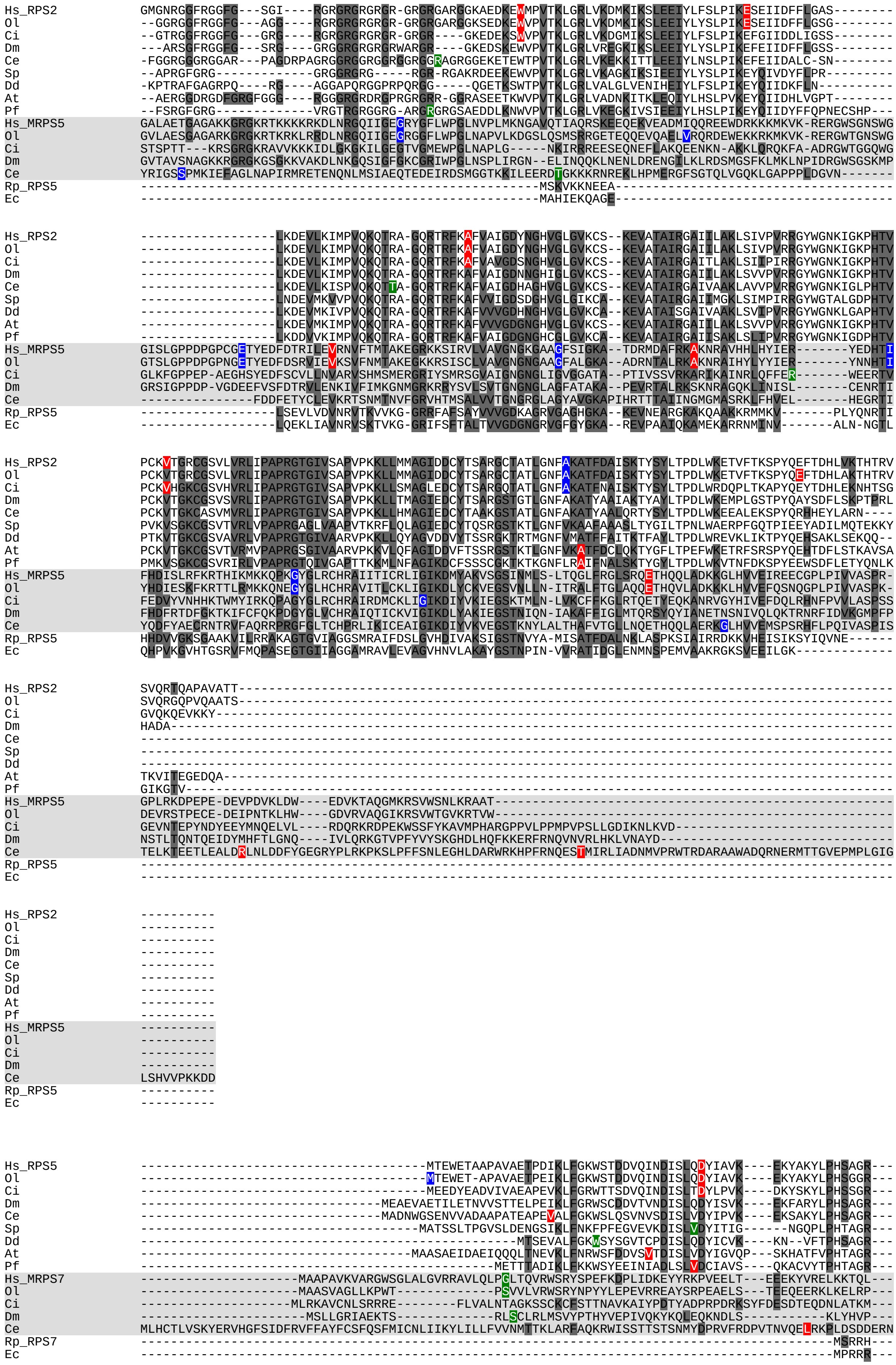

Figure S1. Yoshihama *et al.*

```
Hs_RPS5     ---YAAKRFRKAQCPIVERLTNSMMMHGRNNGKKLMTVRIVKHAFEIIHLLT---------------GENPLQVLVNAIINSGPREDSTRIGRAGTVRRQ
Ol          ---YAAKRFRKAQCPIVERLTNSMMMHGRNNGKKLMTVRIVKHAFEIIHLLT---------------GENPLQVLVNAIINSGPREDSTRIGRAGTVRRQ
Ci          ---YQIKRFRKAQCPIVERLVNSMMMHGRNTGKKLLTVRIIKHAFEIIHLLT---------------GENPLQVLVNAIINSGPREDSTRIGRAGTVRRQ
Dm          ---YAAKRFRKAQCPIVERLTCSLMMKGRNNGKKLMACRIVKHSFEIIHLLT---------------GENPLQILVSAIINSGPREDSTRIGRAGTVRRQ
Ce          ---FQVRRFRKAACPIVERLANSLMMHGRNNGKKLMAVRIVKHAFEIIYLLT---------------GENPLQVLVNAVINSGPREDSTRIGRAGTVRRQ
Sp          ---FQTKRFRKARCFIVERLTNSLMMNGRNNGKKLLATRIVKHAFEIIALLT---------------DQNPLQVLVDAVAACGPREDSTRIGSAGTVRRQ
Dd          ---YNKVRFRKAQCPIVERLANSMMMFGRNAGKKVMAVRIIEQAFEIIYLLT---------------DKNPLQVLVEAVMNSGPREDSTRIGSAGTVRRQ
At          ---YSVKRFRKAQCPIVERLTNSLMMHGRNNGKKLMAVRIVKHAMEIIHLLS---------------DLNPIQVIIDAIVNSGPREDATRIGSAGVVRRQ
Pf          ---YQKKRFRKALCPIVERLVNSMMMHGRNNGKKLKAIRIVAYAFEIIHLMT---------------GENPLQVFVNAVQKGGPREDSTRIGSAGVVRRQ
Hs_MRPS7    ---IKAAPAGKTSSVFEDPVISKFTNMMMIGGNKVLARSLMIQTLEAVKRKQFEKYHAASAEEQATIERNPYTIFHQALKNCEPMIGLVPILKGGRFYQV
Ol          ---IKAATCSTTSSVFDDPLISKFVRMIMTDGNKVLAREITQTLENIKRKQVEKYHKSPAGKKEEIECNPYTIFHTALENCKPVVGLASIQKGGKSYQV
Ci          ---VKAAPNTFTYSVFYDPVLYKFEKMILKKGKGALAKKILQDALTNIKIAQLKKYHAASPEEKESIEVDPLVVFHTAIENASPILGLTRVKKGSMTYQI
Dm          ---IKAAVNKSSDTIFHDDTKHKMINYITKKGNSALARTLLSKTLELIKRTQTEHMNLAK-GEKTTINTNPETLLKQAVENCRPLLQVTAIKRGGVTYQV
Ce          FLFLKAMKSDATPVFYRDHVIDKLIRVCTKDGEKETSRKNVLSALEIIKRRQYKAWAKASEEEKKSIELDPFLVAKKGIKNCHPLMKLQGVTRGGTTYQV
Rp_RPS7     ---VAEKRIILPDMKYNSILLSRFINNIMKAGKKAVAEKIVYSALNKIEKK---------------HSVDPYQTFNNAMHNVKPHLEVTSVRVGGANYQV
Ec          ---VIGQRKILPDPKFGSELLAKFVNILMVDGKKSTAESIVYSALETLAQR---------------SGKSELEAFEVALENVRPTVEVKSRRVGGSTYQV

Hs_RPS5     AVDVSPLRRVNQAIWLLCTGAREAAFRNIKTIAECLADELINAAKGSSNSYAIKKKDELERVAKSNR------------------------------
Ol          AVDVSPLRRVNQAIWLLCTGAREAAFRNIKTIAECLADELINAAKGSSNSYAIKKKDELERVAKSNR------------------------------
Ci          SVDVSPLRRVNQAIWLLCTGAREASFRNIKTIAECLADELINAHKGSSNSYAIKKKDELERVAKSNR------------------------------
Dm          AVDVSPLRRVNQAIWLLCTGAREAAFRNIKTIAECLADELINAAKGSSNSYAIKKKDELERVAKSNR------------------------------
Ce          AVDVAPLRRVNQAIWLLCTGAREAAFRNVKTIAECLADELINAAKGSSNSYAIKKKDELERVAKSNR------------------------------
Sp          AVDVSPLRRVNQALALITIGAREAAFRNVKSISECLAEEIINAAKGSSNSYAIKKKDELERVAKSNR------------------------------
Dd          AVDVSPMRRVNHAVYLLTQGTRAAAFRNIRTVAECLADELINASKGSPNSYSIKQKDALERTAKSHR------------------------------
At          AVDISPLRRVNQAIFLLTTGAREAAFRNIKTIAECLADELINAAKGSSNSYAIKKKDEIERVAKANR------------------------------
Pf          AVDVSPLRRVNQAIYLICTGARNAAFRNIKSISECLAEEIINCANESSSSYAIKKKDEIERVAKANR------------------------------
Hs_MRPS7    PVPLPDRRRRFLAMKWMITECRDKKHQRT-LMPEKLSHKLLEAFHNQGP--VIKRKHDLHKMAEANRALAHYRWW---------------------
Ol          PIPLTDNRRRFLAMKWMITECRENKHRRT-HMYEKLSQELLAAFEKEGN--VIKKRHELHKMAEANRAYAHYRWW---------------------
Ci          PHVLSDEKRLFLVSTWMLKYIRSNRPK-N-EAGIRIGNELLAAYNNQGT--TVKRKQALHKSAEANRAYINIRA----------------------
Dm          PVPITTKRSYFLAMKWLLEAARE-KERKV-SLPEKLAWEILDAAHGQGR--VIKRKDDLHRLCESNRAYAHYRWS---------------------
Ce          PFPIEEPEAEFRAMKMMRDICRVRSKHGETHFKDILATELLAASQNEGS--TIQAKQELHKTCEANRAYAHYRA----------------------
Rp_RPS7     PTHVDERRGYALASRWIINAASKRSEK--MMIDKLAEELFEASNNRGV--AIKKKEDTHKMAEANKAFSHFSPKKMK------------------
Ec          PVEVRPVRRNALAMRWIVEAARKRGDK---SMALRLANELSDAAENKGT--AVKKREDVHRMAEANKAFAHYRWLSLRSFSHQAGASSKQPALGYLN

Hs_RPS11    ---MAD---IQTERAYQKQP-TIFQNKKRVLL---GETGKEKLPRYYKNIGLGFKTPKEAIEGTYIDKKCPFTGNVSIRGRILSGVVTKMKMQ----RTI
Ol          ---MAD---AQTERSYQKQP-TIFQNKKRVLLTDGGKEGKEKLPRYHKSVGLGFKTPRDAIEGTYIDKKCPFTGNVSIRGRILSGVVTKMKMQ----RTI
Ci          ---MADT--AQTERAYQKQP-TVFLNKKRVLP-----SSKKKL-RHVRSIGLGFKTPREAFEGNYIDKKCPFTGNVSIRGRILTAVVTKLKMQ----RTC
Dm          ---MAD----QNERAFQKQF-GVNLNRKVKPG-----ITKKKLLRRSRDVGLGFKTPREAIDGTYIDKKCPWTGDVRIRGRILTGVVRKAKMQ----RTI
Ce          ---MSE----QTERAFLKQP-TVNLNNKARIL-----AGSKKTPRYIREVGLGFKAPRDAVEGTYIDKKCPWAGNVPIRGMILTGVVLKNKMT----RTI
Sp          ---MATELVVQSERAFQKQP-HIFQN---AKKG--------AGRRWYKDVGLGFKTPAEAIYGEYVDKKCPFVGQVSIRGRILTGTVVSTKMH----RTI
Dd          ---MA---DIQAEKAFQKQQ-VSFKNRFQLKASK-----GKEGIRYYKNIGLGYRTPKDAIEGTYIDKKCPFTSDVSIRGAILRGTVISTKMR----RTL
At          ---MAE----QTEKAFLKQP-KVFLSSKKSGK---GKRPGKGGNRFWKNIGLGFKTPREAIDGAYVDKKCPFTGTVSIRGRILAGTCHSAKMQ----RTI
Pf          ---MATTLDVQHERAYQKQEGASFFNSKKIKKG------SKSYIRYWKKVGLGFATPKEAKEGVYVDKKCPFTGNVSIRGRILKGMVISNKMK----RTI
Hs_MRPS17   ---MSVVRSSVHARWIVGKVIGTKMQKTAKVR--------------------VTRLVLDPYLLKYFNKRKTYFAHDALQQCTVGDIVLLRALPV--PRAK
Ol          ---MSVKHASVHAKWIIGKVIGTKMCKTAKVR--------------------VTKLVLDPYLLKYYNKRKTYFAHDALQQCTVGDIVLLKALLE--PRSK
Ci          ----MDKSRFLSMSMALGKVVAKDFTNRAKVV--------------------VQLNRFNPYMNLFFPEAETLESHDPSDKAKVGDIVLLRKLDK--PIRH
Dm          --------MSRRGLLLMGQCMPCIKQNASKIR--------------------IRRMELDKNLNMYFKKDEFYFAHDPQKVCKTGDVVLIRELPE--RLTR
Ce          MPKTSVWKTRVGTQILMGKITDITQIGIDRIP---------------CAQVRCQMNEFNIYLKKYFARSFDFWALDKTSLGNIGDTVLIKQIDGSSRPKA
Rp_RPS17    ----------------------------------------------------------------------------MPK--RVLQGVVISSKTD----KTV
Ec          ----------------------------------------------------------------------------MTDKIRTLQGRVVSDKME----KSI

Hs_RPS11    VIRRDYLHYIRKYNRFEKRHKNMSVHLSPCFRDVQIGDIVTVGECRPLSKTVRFNVLKVTKAAGT----KKQFQKF---------
Ol          VIRRDYLHYIRKYNRFEKRHKNLSVHLSPCFRDVTVGDIVTVGECRPLSKTVRFNVLKVTKAAGA----KKQFQKF---------
Ci          VVRRDYLHYVRKYNRFEKRHQNMSVHISPCFRDVQPGDIATIGECRPISKTVRFNVVKINKAKGAS---KKQFSKF---------
Dm          VIRRDYLHFVRKYSRFEKRHRNMSVHCSPVFRDVEHGDIVTIGECRPLSKTVRFNVLKVSKGQGA----KKSFKKY---------
Ce          VVRRDYLHYIKKYRRYEKRHKNVPAHCSPAFRDIHPGDLVTIGECRPLSKTVRFNVLKVNK-SGTS---KKGFSKF---------
Sp          IIRREYLHFIPKYNRYEKRHKNLAAHVSPAFR-INEGDVVTVGQCRPLSKTVRFNVLRVVKHTE----GPKQFGKF---------
Dd          VVRRNYLHYIKKYNRFEKRHSNIPAHVSPAFN-VKEGDSVTIGQCRPLSKTVRFNVI---KHDISAA-SKKQFKAF---------
At          IVRRDYLHFVKKYQRYEKRHSNIPAHVSPCFR-VKEGDHIIIGQCRPLSKTVRFNVLKVIPAGSSSSFGKKAFTGM---------
Pf          IIRRNYLHYVKKYNRFEKRHKNIPCHCSPCFD-VKEGDIVTVGQCRPLSKTVRFNVLHVEKHQIFGS-ARKQFVLF---------
Hs_MRPS17   HVKHELAEIVFKVGKVIDPVTGKPCAGTTYLESPLS------SETTQLSKNLEELNIS-----------SAQ--------------
Ol          HVKHELAEIVYKVGRVTDPLTGKSVAGTEFLE-PLTD--LQIEEGTTLSERLQELNISATSNGGDST--SAQTPSL---------
Ci          VETHKIESIVYCAGSIVDPLTGLRVERDKYLDIETLSEEKISQLHEDIKEMQVVEKVAHYPRKSEFHWENKDIYEKRK-------
Dm          LITHNVEKVVYPLGDITDPLTGKKVVVGNYREDIEMANQLFGKSEKAKFDYEKAPPRGLEGTKDFTHGETYIKYHEDGKDQPFAV
Ce          NVSHAVDRVVFKFGNIVDPVTGRKIFNDTFADEIDLKKVLVEEVVDKPLEEESMLFEERRALQIRRLEQEKEANV----------
Rp_RPS17    TVKVERKFKHPIYKKFVKVSKKYAAHDI--ENKYKEGDKVSIVESRPISKTKTWVVLNLE-----------------------
Ec          VVAIERFVKHPIYGKFIKRTTKLHVHDE--NNECGIGDVVEIRECRPLSKTKSWTLVRVVEKAVL-------------------

Hs_RPS13    ----------------------------------------------------------------MGRMHAPGKGLSQSALPYRRSVPTWLKLTSDDVKEQ
Ol          ----------------------------------------------------------------MGRMHAPGKGLSQSALPYRRSVPTWLKLTSDDVKEQ
Ci          ----------------------------------------------------------------MGRMHAPGKGLSSSALPYRRSVPTWLKLSSEDVKEQ
Dm          ----------------------------------------------------------------MGRMHAPGKGISQSALPYRRTVPSWLKLNADDVKEQ
Ce          ----------------------------------------------------------------MGRMHNPGKGMAKSAIPYRRSVPSWQKMTAEEVQDQ
Sp          ----------------------------------------------------------------MGRMHSKGKGIASSALPYVRSPPAWCKADADSVVEQ
Dd          ----------------------------------------------------------------MGRMHSNGKGISGSSLPYNRKPHAWTKPTASEVCET
At          ----------------------------------------------------------------MGRMHSRGKGISASALPYKRSSPSWLKTTPQDVDES
Pf          ----------------------------------------------------------------MGRMYGKGKGISSSTLPYKRKQPSWLKQPSEIEDA
Hs_MRPS15   ---------MLRVAWRTLS-LIRTRAVTQVLVPGLPGGGSAKFPFN-----------QWGLQPRSLLLQAARGYVVRKPAQSRLDDDPPPSTLLKDYQ
Ol          -----MLTNVGLRVFLKSSTNIFRGSSSLSSAASLLPRRGPAALTPDSRGCCFPLAFNAPEGFAASQPVRQYARVAKTKTDTGSQLSDLTPEMLKLDYA
Ci          -------MIKLQRFLISTMQNMRIKDCVQPSCHVMQIRMKSSRKRGRMRG------------GHKPMPVPPERLEWSQFDALDKKQPRVELKGMPLLEE
Dm          -------MNKLLNIAQAGHRQFVREYAFKS----------------------------DLKIKWMRPEKIACYKPEKSGDLAKLPPLKADELLPEYR
Ce          MINLRRFVHTSCRLERGRTAFYNVHQKVTDPAKQDPDYFEKKARELPLDQNYIDALTKLYYEKIGSERDLGLKAADNLILEKTEFGLPRIEKSKTRAKYE
Rp_RPS15    ----------------------------------------------------------------------------------------------------
Ec          ----------------------------------------------------------------------------------------------------

Hs_RPS13    IYKLAKKGLTPSQIGVILRDSHGVAQVRFVTGNKILRILKSKGLAPDLPEDLYHLIKKAVAVRKHLERNRKDKDAK-----FRLILIESRIHRLARYYKT
Ol          IFKLAKKGLTPSQIGVILRDSHGVAQVRFVTGNKILRILKSKGLAPDLPEDLYHLIKKAVAVRKHLERNRKDKDAK-----FRLILIESRIHRLARYYKT
Ci          IYKLAKKGLRPSQIGVILRDSHGVAQVRFVTGNQILRVLKAKGLAPDLPEDIYHLIKKAVAMRKHLERNRKDTDSK-----FRLILVESRIHRLGRYYKT
Dm          IKKLGKKGLTPSKIGIILRDSHGVAQVRFVNGNKILRIMKSVGLKPDIPEDLYHMIKKAVAIRKHLERNRKDKDGK-----FRLILVESRIHRLARYYKT
Ce          IVKMAKKGLRPSQIGVILRDSHGVGQVRRLAGNKIFRILKSKGMAPELPEDLYHLVKKAVAIRKHLERSRKDIDSK-----YRLILVESRIHRLARYYKT
Sp          ILKFSKKGMSPSQIGVTLRDSHGIPQVRFITGQKIMRILKANGLAPELPEDLYNLIKKAVSVRKHLERNRKDKDSK-----FRLILIESRIHRLARYYRK
Dd          VCKLAKRGYTPSKIGSSLRDSLGVAQVKNVTGSKILRILKVNGLAPTIPEDLYHLIKKAVTINKHLQRARKDYDGK-----FHLRLVESRIHRLTRPYRK
At          ICKFAKKGLTPSQIGVILRDSHGIPQVKSVTGSKILRILKAHGLAPEIPEDLYHLIKKAVAIRKHLERNRKDKDSK-----FRLILVESRIHRLARYYKK
Pf          IIKLAKKGQTPSQIGATLRDNYGIPQVKSVTGNKILRILRAQGIATTIPEDLYFLIKKAVSMRKHLEKNKKDKDCK-----FRLILTESKIHRISRYYKR
Hs_MRPS15   NVPGIEKVDDVVKRLLSLEMANKKEMLKIKQEQFMKKIVANPEDTRSLEARIIALSVKIRSYEEHLEKHRKDKAHK-----RYLLMSIDQRKKMLKNLRN
Ol          AVPLAQTTDDIVKRLLSLELASHSEKLQLKKEQLIAKVQRDETDRSSVEVQIAILTARIRNYGEHLQKHHKDKANK-----RRMLMAIDRRKKLLKNLRL
Ci          SEKSGKSTDARVLKVLSLEFGNQKDLEKAKVLEYGKHLQRHPNDFDSPEMQVAQLTVHIRTVQEHYARIFRQDKKLP----GAVKKMVERRNDLLDFLHL
Dm          DCKELDKADESVKSLFKLSNNASYLTTKFYRDEMVKEVQRHAQDFGSMEAKLAKMTAVIRRYQEHMDKHPRDKMIK-----VRLKELIDKRKKFLKYLRR
Ce          DLDVLSNAPESVKKIFSVEMATRKELSQEWKQSLIKSVRQHSLDENSLEMKIAWLTALIRHWSLLVNDIGQETKKPTWLTHRIWLVINERRKALRILRE
Rp_RPS15    ----------------MSITTE------RKQQLIKEYAITENDTGSTAVQCAILTERINNLTEHFKSNHKDHTSR-----RGLLILVGRRRRLLNYIKK
Ec          ----------------MSLSTE------ATAKIVSEFGRDANDTGSTEVQVALLTAQINHLQGHFAEHKKDHHSR-----RGLLRMVSQRRKLLDYLKR
```

Figure S1. Yoshihama *et al.*

```
Hs_RPS13    KRVLPPNWKYESSTASALVA--------------------------------------------------------------------------------
Ol          KRVLAPNWKYESSTASALVA--------------------------------------------------------------------------------
Ci          KGVLPPNWKYESATASALVA--------------------------------------------------------------------------------
Dm          KSVLPPNWKYESSTASALVA--------------------------------------------------------------------------------
Ce          KRQLPPTWKYESGTAASLVS--------------------------------------------------------------------------------
Sp          VGALPPTWKYESATASALVA--------------------------------------------------------------------------------
Dd          NGTLAPNWKYESNNASTLVA--------------------------------------------------------------------------------
At          TKKLPPVWKYESTTASTLVA--------------------------------------------------------------------------------
Pf          KKLLPSNWKYQSSTASALIA--------------------------------------------------------------------------------
Hs_MRPS15   TNYDVFEKICWGLGIEYTFPPLYYRRAHRR-----------FVTKKALCIRVFQETQKLKKRRRALKAAAAAQKQAKRRNPDSPAKAIPKTLKDSQ----
Ol          VRYETFERVCEQLGITYTFPPEYYRRATRR-----------WLAKKEFCIKVFKAVQKQK----AEQNLKMKQNLKTTETEETKATAV------------
Ci          YRFETYTKVLKLFKIKHKLPPLETR-------------IRSELIDKVTDMRIRAFAESRRVKFEEEERKRQLSELKQHLKEQRTVDK-------------
Dm          WDYPRFEWILEKLDLVYKPPPTHFHWITRKESLQKLTDIYCENLKEERLEAYHKQLQAQQIPFLEEAIKKMQFVRQEQISCDVPVTVTEEKIADSKRQLE
Ce          RNETAFEKTIAALKISYHVPKQPAHVKTRKAWAEAQLKLRVENEKEKRLEELHEKYDKEVEEHKRETQEKRKALNNELDKLAQEMRQIDVIEGKSFETVG
Rp_RPS15    NNVSEYLDLISKLGIRKIK---------------------------------------------------------------------------------
Ec          KDVARYTQLIERLGLRR-----------------------------------------------------------------------------------

Hs_RPS13    ------------------------------
Ol          ------------------------------
Ci          ------------------------------
Dm          ------------------------------
Ce          ------------------------------
Sp          ------------------------------
Dd          ------------------------------
At          ------------------------------
Pf          ------------------------------
Hs_MRPS15   ------------------------------
Ol          ------------------------------
Ci          ------------------------------
Dm          MLKELQQAEAAASSKKQNEDGFN-------
Ce          KYEPALISSLTETVIHSNLFYHPPPTMTEK
Rp_RPS15    ------------------------------
Ec          ------------------------------


Hs_RPS14    --------------------------------------------------------------------MAP-RKGKEKKEEQVISLGPQVAEGENVFGVC
Ol          --------------------------------------------------------------------MAP-RKGKEKKEEQVISLGPQVAEGENVFGVC
Ci          --------------------------------------------------------------------MAP-RKAKVEKTEEVVQLGPQVAEGENVFGVA
Dm          --------------------------------------------------------------------MAP-RKAKVQKEEVQVQLGPQVRDGEIVFGVA
Ce          --------------------------------------------------------------------MAPARKGKAKEEQAVVSLGPQAKEGELIFGVA
Sp          --------------------------------------------------------------------MAT-------------NVGPQIRSGELVFGVA
Dd          --------------------------------------------------------------------MSGKKEVKAPKVVEALSAKIPCGPSELVFGVA
At          --------------------------------------------------------------------MSK-RKTKEPK-VDVVTLGPSVREGEQVFGVV
Pf          --------------------------------------------------------------------MAS-KKVKTPQPETAIVSGPQPKEGELVFGVA
Hs_MRPS11   MQAVR-------NAGSRFLRSWTWPQTAGRVVARTPAGTICTG-ARQLQDAAAK-QKVEQNAAP----SHTKFSIYPPIPGEESS-LRWAGKKFEEIPIA
Ol          MYKLG-------CVLGNSVRTVCR-QLAVSVNAHTTSINGSTDLQRTIHSSAVRLQEAEAPKEN----SGKDFSLLPPLPGEDSA-LKWGGKKFEELPIV
Ci          --MIRSCAAALLRANCKIGAPKPYHGVLSINRSIHQQEKQDVQFPYLIQDRVYEKVRTQNFAPIPIAKCSKKEDALNAVEFEKRNNLSYDGIPYSELPIV
Dm          MSLK--------SVFLSALRSITMPAVPLQTSRIHTSACWRKAEDRKEMLASLPAKDEGTVGEKTVDIDTLINRKAKFFPDASTANTLFNGIPFNELPIC
Ce          MSLRRLVSDLGRMCLQTASQSMEIQQNRAIHASIRAADSIRDAHRQGTRIGTGQVETMEGTTGAGIQTAVNTSSIDARLPTAETLKQQFDGVAYHELPLV
Rp_RPS11    ---------------------------------------------------------------------------------MNQTIKVKKKKKTITLGVV
Ec          --------------------------------------------------------------------MAK----------APIRARKRVRK-QVSDGVA

Hs_RPS14    HIFASFNDTFVHVTDLSGKETICRVTGGMKVKADRDESSPYAAMLAAQDVAQRCKELGITALHIKLRATGGNRTKTPGPGAQSALRALARSGMKIGRIED
Ol          HIFASFNDTFVHVTDLSGKETICRVTGGMKVKADRDESSPYAAMLAAQDVAQRCKELGITALHIKLRATGGNRTKTPGPGAQSALRALARSGMKIGRIED
Ci          HIYASFNDTFVHVTDLSGKETIVRITGGMKVKADRDEASPYAAMLAAQDVAARCKEIGINALHIKLRATGGTRTKTPGPGAQSALRALARSGMKIGRIED
Dm          HIYASFNDTFVHVTDLSGRETIARVTGGMKVKADRDEASPYAAMLAAQDVAEKCKTLGITALHIKLRATGGNKTKTPGPGAQSALRALARSSMKIGRIED
Ce          HIFASFNDTFVHITDISGRETIVRVTGGMKVKADRDESSPYAAMLAAQDVADRCKQLGINALHIKLRATGGTRTKTPGPGAQSALRALARAGMKIGRIED
Sp          HIFASFNDTFVHITDLTGKETIVRVTGGMKVKTDRDESSPYAAMLAAQDAAAKCKEVGITALHIKIRATGGTATKTPGPGAQAALRALARAGMRIGRIED
Dd          HIFASFNDTFVHVTDLSSKETIVRVTGGMKVKTDRDESSPYAATMAAQDVALRCKELGVTALHIKLRATGGNGSKTPGPGAQSALRALARSGMRIGRIED
At          HIFASFNDTFIHVTDLSGRETLVRITGGMKVKADRDESSPYAAMLAAQDVAQRCKELGITAMHVKLRATGGNKTKTPGPGAQSALRALARSGMKIGRIED
Pf          HIFASFNDTFIHVTDLSGRETLVRITGGMKVKADRDESSPYAAMMAAQDVAARLKELGVTAIHIKLRASGGTKSKTPGPGAQSALRALARSGLKIGRIED
Hs_MRPS11   HIKASHNNTQIQVVSASNEPLAFASCGTEGFRNAK-KGTGIAAQTAGIAAAARAKQKGVIHIRVVVKG--------LGPGRLSAMHGLIMGGLEVISITD
Ol          HIKATYNNTHIQLTDSEGQSLVRTSCGTEGFKNIK-KSTPIAAQTAGISAAAKATGKGVTFVRVVVKG--------IGPGRQSAIKGLAMGGLEVVSITD
Ci          LIQARWNNTLIETK-YRKEVFSYVSCNREGFKNAKKKTDHASEMTGKAGAQKALIAGCHKHVRVVLRG--------IGPGRKAAIKGLTLGGMNVVSITD
Dm          NIRVSPNNTIISVTDHKGVLRLIRSCGIEGFKNTR-KGTNIAAQATAVTISGKAIELGWKTVRVKVRG--------LGPGRMSAIKGLQMGGLNIVSITD
Ce          YIRASKNNTLVTVMDNKNEVITSTSCRLEGFKNAR-KKTTIAGQTTGVAAGQRLVRRGIRTVRVQVKG--------LGPGRMTCVKGLTVAGVHVVSISD
Rp_RPS11    HIRASFNNTIVTFTDIQGNTISSASAGGNGFKGAR-KATPYAAQVTIDKASEKAKECGLKTISIRIGG--------PGAQRESAMRALFGQNFVVTSILD
Ec          HIHASFNNTIVTITDRQGNALGWATAGGSGFRGSR-KSTPFAAQVAAERCADAVKEYGIKNLEVMVKG--------PGPGRESTIRALNAAGFRITNITD

Hs_RPS14    VTPIPSDSTRRKGGRRGRRL
Ol          VTPIPSDSTRRKGGRRGRRL
Ci          VTPIPSDHTRRKGGRRGRRL
Dm          VTPIPSDSTRRKGGRRGRRL
Ce          VTPIPSDCTRRKGGRRGRRL
Sp          VTPIPTDSTRRKGGRRGRRL
Dd          VTPIPTDSTRRKSGHRGRRL
At          VTPIPTDSTRRKGGRRGRRL
Pf          VTPIPTDSTRKKSGRRGRRL
Hs_MRPS11   NTPIPHNGCRPR---KARKL
Ol          NTPVPHNGCRPR---KARRM
Ci          TTPITNNG-LPQRPRKIRRL
Dm          NTHVSFNPPRPR---KQRSL
Ce          HTPLCELGPRPR---KIRRI
Rp_RPS11    VSSIAHNGVRPP---KRRRV
Ec          VTPIPHNGCRPP---KKRRV


Hs_RPS16    ----------------------------------------------------------------------------------------------------
Ol          ----------------------------------------------------------------------------------------------------
Ci          ----------------------------------------------------------------------------------------------------
Dm          ----------------------------------------------------------------------------------------------------
Ce          ----------------------------------------------------------------------------------------------------
Sp          ----------------------------------------------------------------------------------------------------
Dd          ----------------------------------------------------------------------------------------------------
At          ----------------------------------------------------------------------------------------------------
Pf          ----------------------------------------------------------------------------------------------------
Hs_MRPS9    MAAPCVSYGGAVSYRLLLWGRGSLARKQGLWKTAAPELQTNVRSQILRLRHTAFVIPKKNVPTSKRETYTEDFIKKQIEEFNIGKRHLANMMGEDPETFT
Ol          MAAARVCS---VVPVLLKCGNYSFSVN-----VATAKLGKQVLCRNISQNS---ALCRKNLAAAGPEKFTAEFIQKQIEEFNIGKRHLANIMGEDPENFS
Ci          --------------------MSVVNKYRRLIVTQQNLSRLISTCSREYARRILRMDKSYTNLQKMKQIKYPQDYIEKVVEYKLGTRRLAVMMGQDETGFD
Dm          -MALRVFGLNIVKNNRNLLQNACKSRAPMPCYMAAAPFATDVTVQAAPAAVQKQRVSKAMRAYLKRATEHDEFMKTQHLEFQIGKRHLANMMGADAETFT
Ce          -------------------------MLRIVLARSSRAMSSASPASASDSDTSVRKIGKALETYLKHSQQHVAMMEKHRAEFETGRRHLAKMMSLDIHELD
Rp_RPS9     ----------------------------------------------------------------------------------------------------
Ec          ----------------------------------------------------------------------------------------------------
```

Figure S1. Yoshihama *et al.*

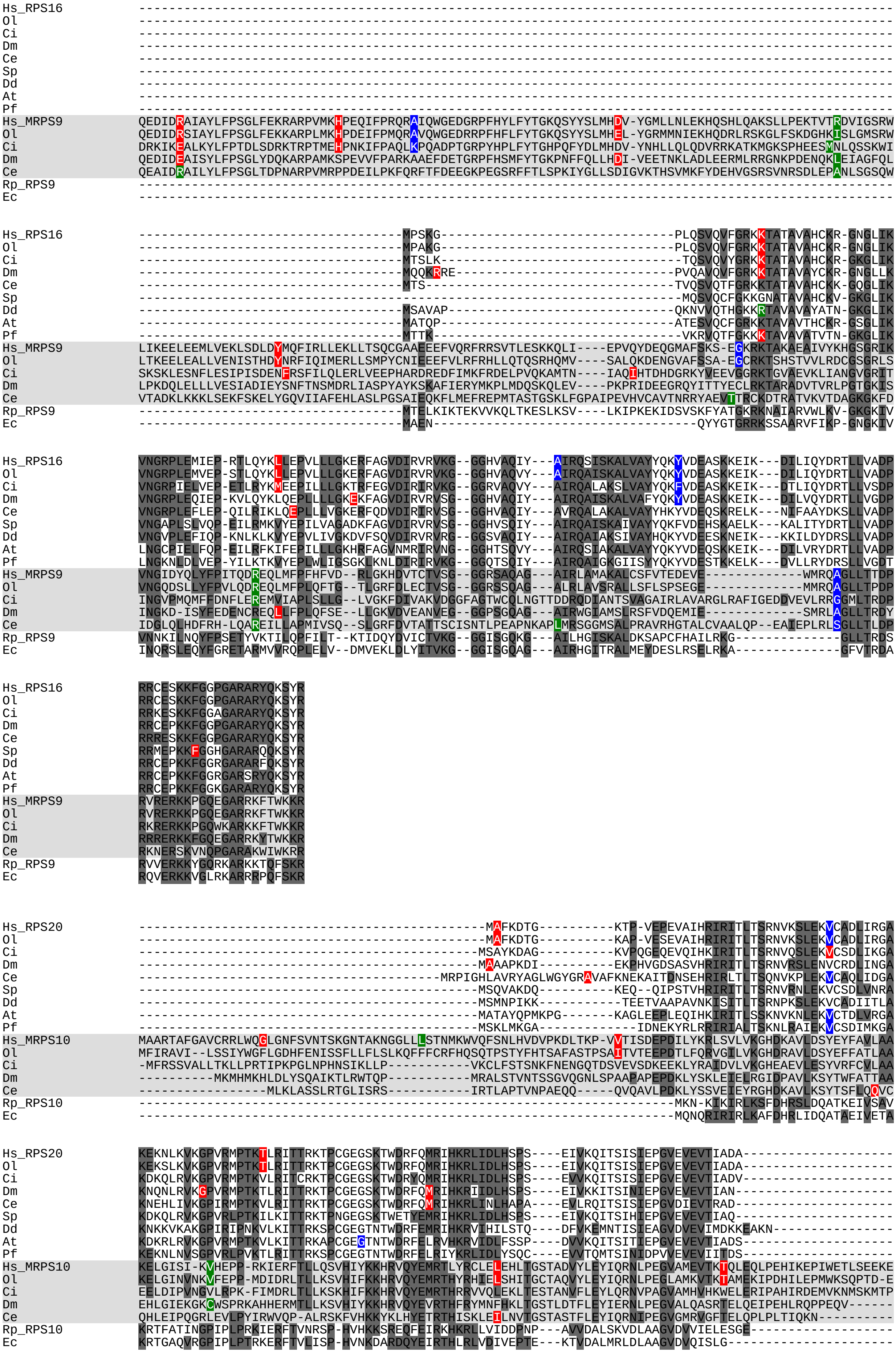

Figure S1. Yoshihama *et al.*

```
Hs_RPS20     ---------------------------------------
Ol           ---------------------------------------
Ci           ---------------------------------------
Dm           ---------------------------------------
Ce           ---------------------------------------
Sp           ---------------------------------------
Dd           ---------------------------------------
At           ---------------------------------------
Pf           ---------------------------------------
Hs_MRPS10    ESKS-----------------------------------
Ol           PSQ------------------------------------
Ci           EDWKRESKYAEKMSIKKKTSNSDYEEYHTTKKYLLGTTI
Dm           ---------------------------------------
Ce           ---------------------------------------
Rp_RPS10     ---------------------------------------
Ec           ---------------------------------------

Hs_RPS23     ---------------------------MG--KCRGLRTARKLRSHRRDQKWHDKQYKKAHLGTALKANPFGGASHAKGIVLEKVGVEAKQPNSAIRKCVR
Ol           ---------------------------MG--KCRGLRTARKLRNHRREQKWHDKQYKKAHLGTALKANPFGGASHAKGIVLEKIGVEAKQPNSAIRKCVR
Ci           ---------------------------MG--KPSGLRCARKLKNRRRDQKWHDKDYKKSHLGTALKANPFGGASHAKGIVLEKIGVEAKQPNSAIRKCVR
Dm           ---------------------------MG--KPRGLRTARKHVNHRRDQRWADKDYKKAHLGTRWKANPFGGASHAKGIVLEKVGVEAKQPNSAIRKCVR
Ce           ---------------------------MG--KPKGLCTARKLKTHRQEQRWNDKRYKKAHIGTRWKSNPFGGASHAKGIVLEKIGVEAKQPNSAIRKCVR
Sp           ---------------------------MG--KPAGLNAARKLRNHRREERWADAHYKKRLLGTAYKSSPFGGSSHAKGIVVEKIGVEAKQPNSAIRKCVR
Dd           ---------------------------MG--KPNGLKTARKLRNHRRVQRWNDKLYKKAHFGTALKANPFGGASHASGIVVSRLGIEAKQPNSAIRKCVR
At           ---------------------------MG--KTRGMGAGRKLKQLRITQRWADKHYKKSNRGNEWK-KPFACSSHAKGIVLEKIGIEAKQPNSAIRKCAR
Pf           ---------------------------MGSGKPSGLRAARKLRIRRRTQRWADKSYKKSHLGTRWKSNPFRGSSHAKGIVVEKVAIEAKQPNSAYRKCVR
Hs_MRPS12    -----------MSWSGLLHGLNTSLTCGPALVPRLWAT---C-SMATLNQMH-RLGPPKRPPRKL--GPTEGRPQLKGVVLCTFTRKPKKPNSANRKCCR
Ol           ------------MWS--LFSLRPALTHVSQLAS-AWTSPLLCRTMATLNQMH-RQGKPKPMPKSL--GATFGRPQLKAVVLKTMIRKPKKPNSANRKCAR
Ci           ------------------MAMKKQRSN--------FLT---YEEIDRRMMLY-GDGHYFKKKKKK--SKMDGRPQMKGVVLKVMIKKPKKPNSANRRCCK
Dm           -----------MNFLRQSFGITKQLASQAIQCS--YET--AVRGMASLQQMH-RSGPHIKTRPPR--QPLDGKPFAKGVVLKTLIKKPKKPNSANRKCVL
Ce           MLPRLGGFLAGFSSTISSFLPKTTASTLFTHQSSQFSTSFPAWRNNFLQFIHYRNGPPKRRARSKDKSAISGYSHYKGIVLKTVIRHPKKPNSGNRKCAI
Rp_RPS12     --------------------------------------------MPTYNQLV-RFGRKSKTRKTK-SPALESNPFKSGVCLVVKTVTPKKPNSALRKIAT
Ec           --------------------------------------------MATVNQLV-RKPRARKVAKSN-VPALEACPQKRGVCTRVYTTTPKKPNSALRKVCR

Hs_RPS23     VQLIKNGKKITAFVPNDGCLNFIEENDEVLVAGFGRKGHAVGDIPGVRFKVVKVANVSLLALYKGKKERPS------------
Ol           VQLIKNGKKITAFVPNDGCLNFIEENDEVLVAGFGRKGHAVGDIPGVRFKVVKVANVSLLALYKGKKERPS------------
Ci           VQLIKNGKKITAFVPNDGCLNYIEENDEVLVAGFGRSGHAVGDIPGVRFKIVKVANVSLHALFTGKKERPS------------
Dm           VQLIKNGKKITAFVPRDGSLNYIEENDEVLVAGFGRKGHAVGDIPGVRFKVVKVANVSLLALYKEKKERPS------------
Ce           VQLIKNGKKITAFVPNDGCLNFVEENDEVLVSGFGRSGHAVGDIPGVRFKIVKVANTSLIALFKGKKERPS------------
Sp           VQLIKNGKKVTAFVPHDGCLNFVDENDEVLLSGFGRKGKAKGDIPGVRFKVVKVAGVGLSALFHEKKEKPRA-----------
Dd           VQLKKNGKKITAFVPNDGCLNYIQENDKVLVAGLGRSGHSVGDIPGVRFKVVKVSSVSLIAIYRGIKDKPNV-----------
At           VQLIKNGKIAAFVPNDGCLNYIEENDEVLIAGFGRKGHAVGDIPGVRFKVVKVSGVSLLALFKEKKEKPRS------------
Pf           VQLIKNGKKITAFVPGDGCLNFIDENDEVLVSGFGRSGHSVGDLPGVKFKVVKVARVSLLALFKEKKEKPRS-----------
Hs_MRPS12    VRLS-TGREAVCFIPGEG--HTLQEHQIVLVE-----GGRTQDLPGVKLTVVRGKYDCGHVQKK-------------------
Ol           VRLS-NGKEAVVFIPGEG--HNLQEHNVVLVQ-----GGRTQDLPGVKLTVVRGKYDCAHVVKKKQ-----------------
Ci           LRLS-NGKEVTAYIPGEG--HNLQQHNVVLVR-----GGKRQDLIGVHYRVMRGKLDCGPVIKRTS-----------------
Dm           VRLS-TGKEMVAYIPGIG--HNLQEHNIVLCR-----VGRLQDVPGVKLKAVRGVYDLAHVVKKSQ-----------------
Ce           VRLS-TGAEVCAYIPNVG--HNLQEHSQVLVK-----GGRRRDLISVKANIVRGKYDCAPAGGRK------------------
Rp_RPS12     VRLS-NKRTVNAYIPGEK--HSVKEHDRVLVR-----GGQVPDLPGVKYHIVLGAYDIAGVKGRKQGRSRYGAHRKQVAATKK
Ec           VRLT-NGFEVTSYIGGEG--HNLQEHSVILIR-----GGRVKDLPGVRYHTVRGALDCSGVKDRKQARSKYGVKRPKA-----

Hs_RPS29     ----------------------------------------------------------------------------------------------------
Ol           ----------------------------------------------------------------------------------------------------
Ci           ----------------------------------------------------------------------------------------------------
Dm           ----------------------------------------------------------------------------------------------------
Ce           ----------------------------------------------------------------------------------------------------
Sp           ----------------------------------------------------------------------------------------------------
Dd           ----------------------------------------------------------------------------------------------------
At           ----------------------------------------------------------------------------------------------------
Hs_MRPS14    ------------------------MAAFMLGSLLRTFKQMVPSSASGQVRSH-----------------------------------------------Y
Ol           ------------------------MAARRLTCLGLNAVYSTVFSPNQQMRSY-----------------------------------------------Y
Ci           --------------MFALKYKSLSQELGKVTKVNLTQTRLVASKRKKPYRFKTCSTAKNHVGGPKPPTTETVAYLALPEAYDPVLWGKKGDANGKNCLWH
Dm           -----------------------MNSLARIGGFVCQSVQIAGCG-LQQVRTK-----------------------------------------------Y
Ce           MLRALRGAADLLSTALYKNEMMMSRRYLSTPAPEPAKPSSEETTESTEPATSVEDAGEPMKEKRITQPYSSEALTKLKLDQYPLYVEREWWKTGKRMTFW
Rp_RPS14     --------------------------MAKVS---------------------------------------------------------------------
Ec           --------------------------MAKQS---------------------------------------------------------------------

Hs_RPS29     ----------------------MGHQQLYWS-----------------HPRKFGQG--SRSCRVCSNRHGLIRKYGLN-MCRQCFRQYAKDIGFIKLD-
Ol           ----------------------MGHQQLYWS-----------------HPRKFGQG--SRSCRVCSNRHGLIRKYGLN-MCRQCFRQYAKDIGFVKLD-
Ci           ----------------------MGHQNIWHS-----------------HPRKYGRG--SRFCRITGNKHGLIRKYGLN-ISRQCFREFAKDIGFQKLD-
Dm           ----------------------MGFATLWYS-----------------HPRKYGQG--SRCCRACSNRHGLIRKYGLN-ICRQCFREYANDIGFKKLD-
Ce           ----------------------MGFQNLWFS-----------------HPRKFGPG--SRSCRVCAGHHGLIRKYGLD-LCRRCFREQARDIGFKKLD-
Sp           ----------------------MAHENVWFS-----------------HPRKYGKG--SRQCAHTGRRLGLIRKYGLN-ISRQSFREYANDIGFVKYR-
Dd           ----------------------MAR-ELWLT-----------------HPRNFGPG--SRTCRKCGNHHGIIRKYDLN-MCRRCFRTDAEAIGFNKYR-
At           ----------------------MGHSNVWNS-----------------HPKKYGPG--SRLCRVCGNSHGLIRKYGLN-CCRQCFRSNAKEIGFIKYR-
Hs_MRPS14    VDWRMWRDVKRRKMAYEYADERLRINSLRKNTILPKILQDVADEEIAALPRDSCPVRIRNRCVMTSRPRGVKRRWRLSRIVFRHLADHGQLSGIQRATW
Ol           VDWRMLRDVKRRQMAFDYADERLRINALRKNTILPKELQEIADKEIAALPRDSCPVRIRNRCVMTSRPRGVKRRWRLSRIVFRHLADPNQMSGIFRARW
Ci           VNWLMQRDNRRRHIATYYGALYTRLQCIRYSGHLPLELKQVADQETSGLPRGSDKNWTVNRCVVTSRPRGNKDKWRISRMVFRHFADHGKMSGVKRAMW
Dm           ADWKMIRDVKRRKCVKENAVERLRVNSLRKNDILPPELREVADAEIAAFPRDSSLVRVRERCALTSRPRGVVHKYRLSRIVWRHLADYNKLSGVQRAMW
Ce           ASWRQLRDVKRREQIQEVGADRMRLKAIKFNTILPQAIRDEAAEKMQKARKYDHPRLILNMCQFTGRQRGKIKPYRLSRHLFRRFADRSALSGVQRAMW
Rp_RPS14     ---SIQKNKSRQKKSQSLHNKRSELKSKIYDKSLSLEQRFPLIIALAQLPRNSSSTRIRNRCELTGRPRGVIRKFGISRNKLRELIGRGLVPGVVKASW
Ec           ---MKAREVKRVALADKYFAKRAELKAIISDVNASDEDRWNAVLKLQTLPRDSSPSRQRNRCRQTGRPHGFLRKFGLSRIKVREAAMRGEIPGLKKASW

Hs_RPL3      -----------------MSHRKFSAPRHGSLGFLPRKRSSRHRGKVKSFPKDDPSKPVHLTAFLGYKAGMTHIVREVDRPGSKVNKKEVVEAVTIVETPP
Ol           -----------------MSHRKFSAPRHGSLGFLPRKRSSRHRGKCKSFPKDDPSKPVHLTAFLGYKAGMTHIVREVDRPGSKVNKKEVVEAVTIVETPP
Ci           -----------------MSHRKFSAPRHGSLGFLPRKRSSRHRGKVKAFPRDDPSKPCHLTAFISYKAGMTHVVREVDRPGSKVNKKEVVDAVTILEAPP
Dm           -----------------MSHRKFSAPRHGSMAFYPKKRSARHRGKVKAFPKDDASKPVHLTCFIGYKAGMTHIVREADRPGSKINKKEVVEAVTVLETPP
Ce           -----------------MSHRKFSAPRHGHMGFTPKKRSRTYRGRIKAFPKDDKSKPIHLTAFLGYKAGMTHIVRDVDKPGSKVNKKEVVEAVTIVETPP
Sp           -----------------MSHCKFEQPRHGSLGFLPRKRASRQRGKVKAFPKDDASKPVHLTAFLGYKAGMTHIVRDLDRPGSKMHKREILEAVTVIETPP
Dd           -----------------MSHRKFEAPRHGNLGFRPRKRAARHQGKVKSFPKDDRTQKVHLTAFMGYKAGMTHVVRDLEKPGSKMHKKEIVEAVTIIECPP
At           -----------------MSHRKFEHPRHGSLGFLPRKRANRHRGKVKAFPKDDQTKPCKFTAFMGYKAGMTHIVREVEKPGSKLHKKETCEAVTIIETPA
Pf           -----------------MSHRKFERPRHGSLGFLPRKRCKRLRGKIRSFPKDDKEKPPHFTAFMGYKAGMTHIVREVDKPGSKLHKKEIVEACTIIECAP
Hs_MRPL3     -----MPGWR-------LLTQVGAQVLGRLGDGLGAALGPGNRTHIWLFVRGLHGKSGTW-WDEHLSEENVPFIKQL-VSD-EDKAQLASKLCPLKDEPW
Ol           ---MAASGWR-------FVLQRGSRFILSRTTVESSSQLVG-------CIR--TVKIPTW-FEEHLTEDNQKYMRHS-MAE-EYRRQTAERLCPLKDEPW
Ci           ---MALRTKRGAGQPKNVFASVPTLHALKRGEQYAVRYTDKYGVRSDKFVPKGKLPAPRWHWDYDLTDENKSFLKEI-SNDGNIEMLQQARQSPLKDSDW
Dm           MMRSVIN------------QLLGLRLSGNSSVLVTQVRDKGHLSRPRLRNPQWFLRKEITKYNDLMTAENKQFVNEVGTNNFGVPAVIKDSLVKTAGPTA
Ce           -----------------------------MAFSGHISQQRGRRRTLTPAPWLPPVKESVPKSSVGISQGTKNLLETVIEKETLAASTIAFQNS--QETNV
Rp_RPL3      ----------------------------------------------------------------------------------------------------
Ec           ----------------------------------------------------------------------------------------------------
```

Figure S1. Yoshihama *et al.*

```
Hs_RPL3    MVVVGIVGYVETPRGLRTFKTVFAEHISDECKRRFYKNWHKSK-KKAFTKYCKKWQDEDGKKQLEKDFSSMKKYCQVIRVIAHTQMRLLP-LRQKKAHLM
Ol         MVVVGVTGYVSTPRGLRSFKTIFAEHISDECKRRFYKNWYKSK-KKAFTKYCKKWQDEEGKKQLEKDFASMKKYCQVIRVIAHTQMRLLP-LKQKKSHLM
Ci         MIVVGVIGYIETPRGLRTLKTLFAEHLSDECKRRFYKNWYACK-KKAFTKSCRKWQDEEGKRQIDRDFNQMKKYCKVIRVIAHTQMKLLP-ITQKKSHIM
Dm         MIVVGAVGYIETPFGLRALVNVWAQHLSEECRRRFYKNWYKSK-KKAFTKASKKWTDDLGKKSIENDFRKMLRYCKVIRVIAHSQIRLIK-QRQKKAHVM
Ce         MVIAGVTGYVDTPQGPRALTTIWAEHLSEEARRRFYSNWAKSK-KKAFTKYAKKWQDEDGKKLIEADFAKLKKYCSSIRVIAHTQMKILR-RRQKKAHLV
Sp         MVVVGVVGYVETPRGLRSLTTVWAEHLSEEVKRRFYKNWFKSK-KKAFTKYAKKYAES--TQSINRELERIKKYCSVVRVLAHTQIRKTP-LAQKKAHLM
Dd         MYIVGLVGYVETAQGLKTYKTVWAQHLSDNFRRRLYKNWYKSKSKKAFTKYVKQYETEEGKKSIEASLQAIKKRCSVVRVIAHTQVHKLK-LTQKKAHVL
At         MVVVGVVAYVKTPRGLRSLNTVWAQHLSEEVRRRFYKNWAKSK-KKAFTGYAKQYDSEDGKKGIQAQLEKMKKYATVIRVLAHTQIRKMKGLKQKKAHMM
Pf         MVVVGIVGYRETPKGLRILSTVWANHVSDEFRRRYYKNWYKSE-KKAFTKSLN-----VPEATKNCLLKRIEKYCTVLRAICHTQPSKTP-LRMKKAHIM
Hs_MRPL3   PIHPWEPGSFRVG---LIALKLGMMPLWTKDGQKHVVTLLQVQDCHVLKYTSKENCN--------------GKMATLSVGGKTVSRFRKATSILEFYREL
Ol         PRHEWTEGSRRVG---LVAIKLGMAPIWTKSGERHAVTMLQVQDCHVIRCMSKEEYD--------------GHSAALAVGGKNVSPFYRSEKARELFRNA
Ci         PVLPWQPGSKRCG---AVGIKLGVHPIWFKDGTYANCTLIQIQDCHVIKYFSKEEYN--------------TKTAAVIIGGKNASPFYKNEKYHEFCREA
Dm         NEAVWTPNLRRCG---VIARKIGQYPLWLKNGERIRTTLLQIVDNHVIKYIPPEEYLPAQVP----TVANLHKRGCILVGSETTNPALLTKEYAGIFRNS
Ce         SVVDIPDSSRRVG---LVVRKIGMLPQWTNEGNRILCTVLEVDENHVVSITSPEAWYKSSAVGKRKAFNRHGPMWRVTVGAGNDDPTKYTLGYRRQFVRA
Rp_RPL3    ---------MRTG---IIAQKVGMTSVFNDKGERISLTLVKVDDCQVVGHKTLAKHG----------------YNALVIGVKDQKISKVTKPMKQVFANA
Ec         ----------MIG---LVGKKVGMTRIFTEDGVSIPVTVIEVEANRVTQVKDLANDG----------------YRAIQVTTGAKKANRVTKPEAGHFAKA

Hs_RPL3    EIQVNGGTVAEKLDWARERLEQQVPVNQVFGQDEMIDVIGVTKGKGYKGVTSRWHTKKLPRKT--HRGLRKVACIGAWHPARVAFSVARA-GQKGYHHRT
Ol         EVQLNGGTISDKVDWAREKLEQAVPVNTVFTQDEMIDVIGVTKGHGYKGVTSRWHTKKLPRKT--HRGLRKVACIGAWHPARVAFSVARA-GQKGYHHRT
Ci         EIQVNGGTIPEKVDYARDLLEKPVPVSNIFAKDEMIDIIGVNKGHGEKGVTSRWHTKKLPRKT--HKGLRKVACIGAWHPARVAYSVARS-GQKGYHHRT
Dm         EIQLNGGSIEDKVKWAREHLEKPIQVSNVFGQDEMIDCVGVTKGKGFKGVTSRWHTKKLPRKT--HKGLRKVACIGAWHPSRVSTTVARA-GQKGYHHRT
Ce         EIQVNGGTIEQKVDWAREHLEKQVQVDTVFAQDEMIDTIGVTRGHGFKGVTSRWHTKKLPRKT--HKGLRKVACIGAWHPSRVAFTVARA-GQKGFHHRT
Sp         EIQVNGGSVADKVEWAREHFEKTVDIKSTFEQNEMIDVIGVTRGKGNEGTTARWGTKRLPRKT--HRGLRKVACIGAWHPANVQWTVARA-GNAGYMHRT
Dd         EIQVNGGSIVEKVNFAVANFEKTVNVTGVFAENELIDVIGVTKGKGFNGVIKRWGVRKLPRKT--HKGLRKVACIGAWHPSRVSTTVPRA-GQLGYHHRV
At         EIQINGGTIAQKVDFAYSFFEKQIPIEAVFQKDEMIDIIGVTKGKGYEGVVTRWGVTRLPRKT--HRGLRKVACIGAWHPARVSYTVARA-GQNGYHHRT
Pf         EVQINGGSMKEKLEFVKEMLEKNLPVSTVFNPNEMIDVISVTKGHGTKGVVSRYGVKRLPRKT--HRGLRKVACIGAWHPARVQFQIPRH-GQKGYFHRT
Hs_MRPL3   GLPPKQTVKIFNITDNAAIKPGTPLYAAHFRPGQYVDVTAKTIGKGFQGVMKRWGFKGQPATHGQTKTHRRPGAVATG-DIGRVWPGTKMPGKMGNIYRT
Ol         GVPPKQKVTIFKVSDNAIIKPGTPLYAAHFRPGQYVDVTAKSIGKGFQGVMKRWGFKGQPASHGQTKTHRRPGASGPGGDPAKVFKGKKMPGKMGNVFVT
Ci         GVPVKAKCFRFLISENAALKPGTEITAMHFKPGQFVDCTAQSIGYGFQGVIQRWHFAGGHARK-DKKWMRRPGSIGGS-QIGWVMKGKKMAGQLGGTNEI
Dm         GVLPKKNLARFIVSPEAQLAPGTPLNVNHFRVGDFVDVRGKTVDHGFQGVVKRHGFKGMPASHGVTKTHRRAGNIGGGEKGRVWPGTKMPGHMGNRWRI
Ce         GIPVKEKLGCFLVTEDALPSAGQPLDARHFGVGQYVTATGKTIDWGFQGGMHRWGMRGQ-PTRRTTKSHRRIGSVGSVGDAR-IWPGKRMPGHMGYEWRT
Rp_RPL3    KIAPKTKLKEFRISEDNFIDIASILEVDHFRVGQFVDITATTIGKGFAGSMKRHNFRGLEASHGVSISHRSHGSTGQRQDPGKVFKGKKMAGHMGCNKVT
Ec         GVEAGRGLWEFRLAEGEEFTVGQSISVELFADVKKVDVTGTSKGKGFAGTVKRWNFRTQDATHGNSLSHRVPGSIGQNQTPGKVFKGKKMAGQMGNERVT

Hs_RPL3    EINKKIYKIGQGYLIKDGKLIKNNASTDYDLSDKSINPLGGFVHYGEVTNDFVMLKGCVVGTKKRVLTLRKSLLVQTKRRALEKIDLKFIDTTSKFGHGR
Ol         EINKKIYKIGQGYHTKDGKVVKNNASTEYDLSNKSINPLGGFVHYGQVANDFVMVKGCVVGTKKRVLTLRKSLLVQTSRRALEKIDLKFIDTTSKFGHGR
Ci         EVNKKIYRLGAGIHKEGGKLIKNNAATETDITDKSITPMGGFPHYGEVKNDFVMIKGCCIGPKKRVLTLRKSLITHTKRAALEKINLKWIDTSSKFGHGR
Dm         EINKKIYRIGAGIHTKDGKVIKNNASTEYDLTDKSITPMGGFPHYGEVNNDFVMIKGCCIGSKKRIITLRKSLLKHTKRSALEQIKLKFIDTSSKMGHGR
Ce         IINNKIYRIGKSALTEEG---KNNGSTEFDLTQKTITPMGGFPRYGIVNQDYIMLRGAVLGPKKRLITLRKSLITQTKRVAHEKINLKWIDTSSKTGHGR
Sp         QLNSKIYRIGAGDD-------AKNASTDFDATEKRITPMGGFVRYGVVENDFVMLNGATPGPVKRVLTLRKSLLTHTSRKALEPVSLKWIDTASKFGHGR
Dd         ERNKKIYRIGQAQPEDGK--QISTGKTEFDLTEKTINPMGGFAHYGMVKHEFLMLKGCVAGPRKRALTLRKSITTQTGRAALEKITLKFIDTSSKFGHGL
At         ELNKKIYRLGKVGT------EAHTAMTEYDRTEKDVTPMGGFPHYGIVKDDYLMIKGCCVGPKKRVVTLRQSLLTQTSRLALEEIKLKFIDTASIFGHGR
Pf         ERNKKIYRIGLKTD-------KNSASTDADITEKKITPMGGFPHYGVVNEDFLLLKGCVAGTKKRPITLRKNIVPQVSRDALAQVSLKFIDTSSKIGHGR
Hs_MRPL3   EYGLKVWRIN---------------------TKHNIIYVNG-SVPGHKNCLVKVKDSKLPAYKDLGKN------LPFPTYFPDGDEEELPE---------
Ol         AYGLKIWRVN---------------------TKYNVLYVHG-SVPGHTNCVLKVRDTVLPTRRSTILN------PPFPTYFTE-EEGDLDE---------
Ci         TRGLKVLRIN---------------------TRYNVLYVKG-RIAGHINQFVKIADSSLYKYQPQSEEEKDKLIGPFPRYIPEFSDEELPE---------
Dm         IKGLRVWRIN---------------------TKYNVMWVQGSSVAGPTGGLVYIYDTILPTRKNKEAP-------PFPTFYGE--PQQDGD---------
Ce         VSGLEIVRIN---------------------VDKQVIYVKG-SVPGDIGETILLKDCLQEEKRLKSGP--MPTWAPSLETIEEEPETEPEEGTIPKNLLF
Rp_RPL3    IQNLKIFAVD---------------------TNRKLIMIQG-SIPGHKNSYLLVKDAIKKAAITIA----------------------------------
Ec         VQSLDVVRVD---------------------AERNLLLVKG-AVPGATGSDLIVKPAVKA----------------------------------------

Hs_RPL3    FQTMEEKKAFMGPLKKDRIAKEEGA----------------
Ol         FQTIEEKKAFMGPLKKDRIAKEETA----------------
Ci         FQTHEEKRAFMGPLKKDRLAAAAAAATTTTTTA--------
Dm         FQTPADKLAFMGPLKKDRLKEEAAATTAAAAAATTTSA---
Ce         FQTTAEKRAFMGKLKRDFLAEAEAKA---------------
Sp         FQTPAEAKQFLGTLKKDVA----------------------
Dd         HQTAEDKTKYFG-VKKSRSTKA-------------------
At         FQTSLEKMRFYNRVTK-------------------------
Pf         FQTSEEKVKYYGPLKKDLKA---------------------
Hs_MRPL3   -DLYDENVCQPGAPSITFA----------------------
Ol         -DLYNDDLFVHTEPSVTLS----------------------
Ci         -ELYDDNVLSLNSLPIVS-----------------------
Dm         -DVWYEQVHNFKSESITYENE--------------------
Ce         NEKFSPKLFRFTSPSILFTDADAKKATGRDKTKAKIAKVKK
Rp_RPL3    -----------------------------------------
Ec         -----------------------------------------


Hs_RPL5    MGFVKVVKNKAYFKRYQVKFRRRREGKTDYYARKRLVIQDKNKYNTPKYRMIVRVTNRDIICQIAYARIEGDMIVCAAYAHELPKYGVKVGLTNYAAAYC
Ol         MGFVKVVKNKAYFKRYQVKFRRRREGKTDFFARKRLVVQDKNKYNTPKYRMIVRFSNRDIVCQIAYAKIEGDMIVCAAYSHELPKYGVTVGLTNYAAAYC
Ci         MGFVKVVKNKAYFKRYQVKYRRRREGVTDYFARKRLVVQDRNKYNTPKYRMIVRFTNKDIITQIAYARIEGDVVICAAYAHELPRYGVKVGLTNYSAAYC
Dm         MGFVKVVKNKQYFKRYQVKFRRRREGKTDYYARKRLTFQDKNKYNTPKYRLIVRLSNKDITVQIAYARIEGDRVVCAAYSHELPKYGIQVGLTNYAAAYC
Ce         MGLVKVIKNKAYFKRYQVKLRRRREGKTDYYARKRLTVQDKNKYNTPKYRLIVRITNKDVVAQLAYSKIEGDVVVASAYSHELPRYGLKVGLTNYAAAYA
Sp         MPFIKAVKSSPYFSRYQTKYRRRREGKTDYYARKRLIAQAKNKYNAPKYRLVVRFSNRFVTCQIVSSRVNGDYVLAHAHSSELPRYGIKWGLANWTAAYA
Dd         MGFVKVVKTKAYYSRYQVQFRRRREGKTDYQQRHRLITQDKNKYNSPKYRLVARITNTDVIAQVVYSKIVGDFVLCAAYAHELPRFGVKVGLTNYASCYA
At         MVFVKSTKSNAYFKRYQVKFRRRRDGKTDYRARIRLINQDKNKYNTPKYRFVVRFTNKDIVAQIVSASIAGDIVKASAYAHELPQYGLTVGLTNYAAAYC
Pf         MAYVKVVKNKAYFKRYQVKYRRRREGKTDYRARKALILQDKNKYNAQKLRFVVRKTNSQVICQIACAHIEGDKILAEAKSKELIRYGIPVGLKNYAAAYA
Hs_MRPL18  MALRSRFWGLFSVCRNPGCRFAALSTS-------SEPAAKPEVDPVENEAVAPEFTNRNPRNLELLSVARKE-------------RGW--------RTVF
Ol         MFLNEVSRNVRVLLGQIQRRCPALPANNTTARCMSQAGTDPEPTPDENEVVSPTFVNRNPRNLEQMALAVKD-------------RGW--------KTTW
Ci         -MFASKLLRVPYRKSIRKVVTSAEKLSLTSAAVDTFYQNEKKKSIKTDIVPHKTFINRNPRNLERLALAHKD-------------QGWGKGIPDRPATNW
Dm         -------------------------MSRSARPLTSARVLHKIKELTTPQASTEYVINRNPRNLERLRIAYKP---------------------VGYHLEK
Ce         ----------------------------------------------MAARFARGFVNRNPRNNELMGRQAPN-------------------TGYQFEKDR
Rp_RPL18   -----------------------------------------------MRSAKLKFEKRKSRIRYKISKTSN-----------------------------
Ec         -----------------------------------------------MDKKSARIR-RATRARRKLQELG------------------------------

Hs_RPL5    TGLLLARRLLNRFGMDKIYEGQVEVTGDEYNVESIDG--QPGAFTCYLDAGLARTTTGNKVFGALKGAVDGGLSIPHSTKRFPGYDSESKEFNAEVHRKH
Ol         TGLLLARRLLNKFGLDKVYEGQVEVTGDEFNVESIDG--QPGAFTCYLDAGLARTTTGNKVFGALKGAVDGGLSIPHSTKRFPGYDTESKEFNAEVHRKH
Ci         TGLLLARRLLTKFGLDKIYEGQTEIDGDEYNVEDIDG--QPGAFRAFLDIGLARTTTGARIFGAMKGAADGGLDIPHSVKRFPGYDAESKEFNAEVHRNH
Dm         TGLLVARRVLNKLGLDSLYAGCTEVTGEEFNVEPVDD--GPGAFRCFLDVGLARTTTGARVFGAMKGAVDGGLNIPHSVKRFPGYSAETKSFNADVHRAH
Ce         TGLLLARRHLKTIGLDSTYKGHEELTGEDYNVEEEGD--R-APFKAVLDIGLARTTTGSKIFAVMKGVADGGINVPHSESRFFGFDQESKEYNAEAHRDR
Sp         TGLLVARRALAKVGLADKYEGVTEPEGEFELTEAIED--GPRPFKVFLDVGLKRTSTGSRVFGAMKGASDGGLFIPHSPNRFPGFDIETEELDDETLRKY
Dd         TGLLLARRLLAKLKLADLYKGAEKVDGKVFLVKPVDD--KPRPFKANLDVGLARTSTGAKVFAVLKGAVDGGIYVPHGETRFAGYNAESKKLNAETLRGY
At         TGLLLARRVLKMLEMDDEYEGNVEATGEDFSVEPTDS---RRPFRALLDVGLIRTTTGNRVFGALKGALDGGLDIPHSDKRFAGFHKENKQLDAEIHRNY
Pf         TGLLCARRFLKSLNLDTQFLGVEKVTGEIENNEDKEDEEGRKPIKAFLDVGITRTTTGNRVFAALKGACDGGLNIPHGNNRFPG---SKNEFNPEQLRKN
Hs_MRPL18  PSREFWHRLRVIRTQHHVEALVEHQNGKV-VVSASTR---EWAIKKHLYSTRNVVACESIGRVLAQRCLEAGINFMVYQ---------------------
Ol         PHREFYHRLMFSRTQHHVTAQVFSSSSPVPVLSCSTK---EWALKKELPSTNCVAACQAVGEVLARRCQQAGITRMVYR---------------------
Ci         PTGSYHHRILFEVTGKHTYASLLHYHGNQ-VVAVSTR---EWSIQKMLYKGNDVSACYNIGTIFAERCIRAGISCAAWT---------------------
Dm         PGRSYWHTLEINTSGRYVSGDVKHFENGTILSASTSE----WAIKQQLYKTNDTSAFVNLGRVLAQRCLQSGITEMTCN---------------------
Ce         AARSYIYKVELVEGKSHREGRLVHYQDGVVISASTKE----PSIASQLYSKTDTSAALNIGRVLALRCLQSGIHFAMPG---------------------
Rp_RPL18   -----RMRLSIFKSCRHIYAQIIDDSKSITIAAASTL---DKKIKKIKKSHCNIENAIKVGKEIAKKADSAGIKEVVFD---------------------
Ec         -----ATRLVVHRTPRHIYAQVIAPNGSEVLVAASTV---EKAIAEQLKYTGNKDAAAVGKAVAERALEKGIKDVSFD----------------------
```

Figure S1. Yoshihama *et al.*

```
Hs_RPL5       IMGQNVADYMRYLMEEDEDAYKKQFSQYIKNSVTPDMMEEMYKKAHAAIRENPVYEKKPKKE-----VKKKRWNRPKMSLAQKKDRVAQKKASFLRAQER
Ol            IMGVNVAEYMRYLMEEDEEAYKKQFSRFIKNGVNPDMMEDMYKKAHASIRENPVHEKKPPKE-----FVKKRWNRAKLSLAQKKDRVAQKKASFLRAQEQ
Ci            IFGQHVSEYMTSLEESDEDAFKRQFSQYIKNGITADGVEEMYKKAHASIREDPTPKAKVEKQ-----VPKKRWNRAKLTKSQREDRVRQKKASYLRAIGA
Dm            IFGQHVADYMRSLEEEDEESFKRQFSRYIKLGIRADDLEDIYKKAHQAIRNDPTHKVTAKKSS---AVTKKRWNAKKLTNEQRKTKIAAHKAAYVAKLQS
Ce            ILGKHVADYMTYLKEEDEDRYKRQFSKFLAAGLNADNLVATYQKVHSAIRADASPAAKKAAK------PSKRHTAKRLTYDERKQRVADKKALLLQLKEQ
Sp            IYGGHVAEYMEMLIDDDEERYQKQFSGLIADGIESDQLEDIYAEAYAKIREDPSFQKSGKDAAAFKAESLKHTQ-RKLTAEERKERFNAKVIEAGRA---
Dd            IFGKHVASYMTLLQTEDEDAYKKIFSQYIKNGVAPKDIETIYANAHKAIRANPAPAAK-STK------SYKDIKSKNLSKRTLKQKKARVASIKASYKAR
At            IYGGHVSNYMKLLGEDEPEKLQTHFSAYIKKGVEAESIEELYKKVHAAIRADPNPKKTVKPAP----KQHKRYNLKKLTYEERKNKLIERVKALNGAGGD
Pf            ILGIHVAEYMKTLQEEDNDKYKTHFNDYLKNNIGADDIEQMYLNAHEKIRQNP--EKESKDTKNVKKFVSKHEKPKKLNAKLRKKRVKEKLALYVEKLQ-
Hs_MRPL18     ---------PTP-WEAASDSMKRLQSAMTEGGVVLREPQRIYE---------------------------------------------------------
Ol            ---------AIP-WTYRSDFVQAFRSAMKEGGIVLSEPRRKYIGT-------------------------------------------------------
Ci            ---------IPQNMVDNSDRIKSFCTAVVETGFVLEEPDQISFKSFQPPHVEKKGPRPPRRV--------------------------------------
Dm            ------------VEAVPGSKLQKLLQTIQDNGVSFKEPSRLPNEQPWDEKRHEKPWEVSEDALEAPKSK-------------------------------
Ce            ---------ATKEAIEKSQHQTHFFKALEEEGLTLKEPAHVEHSYETDKTFTWKRYPQKPTRQDKLDEL-------------------------------
Rp_RPL18      ---------RGG--YKYHGIIKALADAAREK-IKF-----------------------------------------------------------------
Ec            ---------RSG--FQYHGRVQALADAAREAGLQF-----------------------------------------------------------------

Hs_RPL5       AAES----
Ol            EAAD----
Ci            GDN-----
Dm            ETEA----
Ce            QE------
Sp            --------
Dd            L-------
At            DDDEDDEE
Pf            --------
Hs_MRPL18     --------
Ol            --------
Ci            --------
Dm            --------
Ce            --------
Rp_RPL18      --------
Ec            --------

Hs_RPL7       ---MEGVEEKKKEVPAVPETL-KKKRRNFAELKIKRLRKKFAQKMLRKARRKLIYEKAKHYHKEYRQMYRTEIRMARMARKAGNFYVPAEPKLAFVIRIR
Ol            ------MADAEKKVPAVPESL-LKRRKAYAAMKAMRVKKLLAEKKARKVTRKLIYKRAEKYHKEYRQMERREIRLARMARKVGNYYVPAEPKLAFVIRIR
Ci            -------MVASKKLPRVPESL-LKKRKEYQAQKAKQAQAAILNKKKNKEKRKIIFKRAEQYVKEYRIKERDGIRLARQARTHNNYYVPDEPKLAFVIRIR
Dm            MPAPVVKKPAAKKLPAVPESK-LKFSKKQISKRVAESKRRLKKAAVIALRKKENLVRAEKYQNEYIKAEQREIKLRRLAKKRNQFYVPAEAKLAFVVRIR
Ce            -------MAPTKKVPQVPETV-LKRRKQRADARTKAAQHKVTVAAKNKEKKTQYFKRAEKYVQEYRNAQKEGLRLKREAEAKGDFYVPAEHKVAFVVRIR
Sp            --MVASSTVPSVASIFAPESL-LKKTKAQKQSREQIVAAAAEKKSARQKKRELIAKRAEAYEAEYRAAEREQIELARKARAEGNYFVPHEPKLIFVVRIR
Dd            -----MSQKVATKTAPVPESI-LKKRATSQKKAVDVAKLGRAQQLRNSRLNSVYFKRAEKYVSEYHKTEREAIRLNRIAKNSGTFYVPPAAKVAFVIRIR
At            --------MVESKVV-VPESV-LKKRKREEEWALEKKQNVEAAKKKNAENRKLIFKRAEQYSKEYAEKEKELISLKREAKLKGGFYVDPEAKLLFIIRIR
Pf            -MADRYENQVENELGKSMTSLRAKKVNKNKELKQAAAKKIKTLKLINKKKRNDLRQRTLRYEEEYESERKKIIELKREARKNNCFYREAEKKVVFVIRLK
Hs_MRPL30     ---------------------------MAGILRLVVQWPPGRLQTVTKGVESLICTDWIRHKFTRSRIPEKVFQASPEDHEKYGGDPQNPHKLHIVTRIK
Ol            -------------------------------MSAVSFLPAKSLKAAVLSPRGWLVS--ARSTFRKARILKEVFAERSKEHDKYGGDPDQPHKLHIVTRVK
Ci            ----------------------------MSVINYIQLRLARMCTRGFHNSAANLVKNSYPLKPSSINETAKKLGFTNAKEMYPEYPLEEPPKLWMITQVQ
Dm            ------------------------------------MNLGRLLNPLRSTACSVRSYGKHNKKFLYKNGQKFEGITYYPRTPDHQDPPVEPAKLFRVQRIK
Ce            ----------------------------------MAKVIRNRWVYRTDNKWTRYLPRRNDAVKEFDYDKNGASLKHYTEEISEEMRPKQASKLWMAWLYR
Rp_RPL30      ----------------------------------------------------------MNNKINNIKIT--------QIKSAIGCKYD--QRFTLIG---
Ec            ----------------------------------------------------------MA---KTIKIT--------QTRSAIGRLPK--HKATLLG---

Hs_RPL7       GINGVSPKVRKVLQLLRLRQIFN--GTFVKLNKASINMLRIVEPYIAWGYPNLKSVNELIYKRGYGKINKKR----IALTDNALIARSLGKYG-IICMED
Ol            GINGVSPKVRKVLQLLRLRQIFN--GVFVKLNKASINMLRIAEPYIAWGYPNLKSVRELIYKRGYGRMRKQR----IALTDNALVEKALGKYG-IICVED
Ci            GINQVSPKVKKVLQLFRLRQIHN--GVFIKLNKPTLQMLRIAEPYIAWGYPNLKTVRELIYKRGHGKVNKSR----IPITDNNIIEDSLGKKG-IICVED
Dm            GINKVAPKVRKVLQLFRLRQINN--GVFIKLNKATINMLRIAEPYITWGYPNLKSVRELIYKRGFVKHNRQR----VPITDNFVIERKLRQAHQIQCVED
Ce            GINQLHPKPRKALQILRLRQINN--GVFVKLNKATLPLLRIIEPYVAWGYPNNKTIHDLLYKRGYAKVDGNR----VPITDNTIVEQSLGKFN-IICLED
Sp            GINNIPPKARKIMQLLRLLQINN--GIFVKFNKAIKEMLQVVEPYVTYGIPNHKTVRELIYKRGFGKVNKQR----IPLSDNAIIEAALGKYS-ILSVED
Dd            GINGVSPKPRKVLKLLRLLQLNN--GVFVKLNKASINMLKLVEPYVAYGFPNLKSIKELIYKRGHLKIDGQR----IPLTSNDMVEKQLGKFG-IICVED
At            GINAIDPKTKKILQLLRLRQIFN--GVFLKVNKATMNMLRRVEPYVTYGFPNLKSVKELIYKRGYGKLNHQR----IALTDNSIVEQALGKHG-IICTED
Pf            GVNKLPPKVRSVFRLLRLLQVHN--GVFVKVNKATKEMLKIVEPYVTYGYPTLSTVRKLLYKRGYVRVGKVRRYARKKIQDNADISKHLGKYN-VHGIED
Hs_MRPL30     STRRRPYWEKDIIKMLGLEKAHT--PQVHKNIPSVNAKLKVVK--------HLIRIKPLKLPQGLPAEENMS-------NTCLKSTGELVVQWHLKPVEQ
Ol            STMRRPYWEKEMVKHLGLQKAHV--PVIHKNTPAVNSQLKFVK--------HLVRIQPLQTPYGLPAEQDMA-------DAFLNSKGELIVRRLLQPVAR
Ci            RLNSRPYWEKEAMKELGLYRVND--ISIQINSTKMNEILWGIK--------HLIKIQPVRMPQGEPTKSDIG-------YTQLKLNGEFEIKKRVECIEN
Dm            PLKGNPYWENRILKDLGLDGKQSD-FTVVKNIPENNARLWKIK--------HLIKVTPVTFPYGEPTAQDVR-------HTILKENGECLVTKDLGPIDT
Ce            DTSAEPKWTKKHVENLFGADFKVGRMEVFRNTELINTELWKIK--------HLIELRPVEFKNGVEPTEDDIF------STSLAPNGQCEVIQGGHVASE
Rp_RPL30      ---------------LGLNKINK--SVILKNTNSIRGMVEKVK--------HLLKIENM-----------------------------------------
Ec            ---------------LGLRRIGH--TVEREDTPAIRGMINAVS--------FMVKVEE------------------------------------------

Hs_RPL7       LIHEIYTVGK---------------RFKEANNFLWPFKLSSPRGGMK-KKTTHFVEG--GDAGNREDQINRLIRRMN-----------
Ol            LIHEIYTVGK---------------NFKPANNFLWPFKLSSPRGGMN-KKTTHFVEG--GDAGNREDQINRMIRRMN-----------
Ci            LIHEIFTVGP---------------NFKAAAAFLWPFKLSSPRGGFQ-KVTNHFVEG--GDCGNREDKINALVRQMN-----------
Dm            LVHEIFTVGP---------------NFKYASNFLWPFKLNTPTGGWR-KKANHYVNG--GDFGNREDQINRLLRKMV-----------
Ce            LAHEIATVGP---------------HFKEATNFLWPFKLNNPTGGWT-KKTNHFVEG--GDFGNREDQINNLLRKMV-----------
Sp            LIHEIYTVGP---------------NFKQAANFLWPFKLSSPLGGWRERKFKHFIEG--GDAGKRDEHINGLVQKML-----------
Dd            IIHEITTCGK---------------HFKQVNNSLWPFKLNCPRGGFN-MKKTPFLQG--GDAGNREHLINNLIHRMN-----------
At            LIHEILTVGP---------------HFKEANNFLWPFQLKAPLGGLK-KKRNHYVEG--GDAGNRENFINELIRRMN-----------
Pf            MVYQLYTCGP---------------VFKKVNNFLWAFKLKPPRKGFK-AKRHAFNEPRPGDWGNREAHINELINRMI-----------
Hs_MRPL30     KAHES-----------------------------------------------------------------------------------
Ol            IS-ES-----------------------------------------------------------------------------------
Ci            PEDVVPVAPEEEWKVVDGFRTCKNCRQKRCLALQMCQKLWFQYGKNNLERIGPVLFNYVENPLKKQKAIEENQLKTEKGSNNIRESAV
Dm            RLAARQDYDE---------------QPKRLDTDLLRKDARLKWLNPW-----------------------------------------
Ce            NDLKLADGKQ-------------WTRKELGRDLNKKYHLYQSVYEQNVHTPNNITVVK------------------------------
Rp_RPL30      ----------------------------------------------------------------------------------------
Ec            ----------------------------------------------------------------------------------------

Hs_RPL8       -----------------------------------------------------------------------------MGRVIRGQRKG-----------A
Ol            -----------------------------------------------------------------------------MGRVIRGQRKG-----------A
Ci            -----------------------------------------------------------------------------MGRVIRSQRKG-----------P
Dm            -----------------------------------------------------------------------------MGRVIRAQRKG-----------A
Ce            -----------------------------------------------------------------------------MGRRIRIQRKG-----------A
Sp            -----------------------------------------------------------------------------MGRVIRAQRKS------------
Dd            -----------------------------------------------------------------------------MGRIIRAQRKGK----------A
At            -----------------------------------------------------------------------------MGRVIRAQRKG-----------A
Pf            -----------------------------------------------------------------------------MGRVIRGQRKG-----------R
Hs_MRPL2      -MALCALTRALRSLNLAPPTVAAPAPSLFPAAQMMNNGLLQQPSALMLLPCRPVLTSVALN----ANFVSWKSRTKYTITPVKMRKSGGRD---------
Ol            -MAVSCLTRALRSLSLSRPVLVSSQVKPRARRPAAPQPKLGALVSSGAGQWRGFLTTASLE----QNRTYWKQREKYSIRPMGMKKTGGRD---------
Ci            MFTQTVKLVTQKLPTVSSSLTFNVRNLRTLPCANSKLNLNIFGPPANLPTEKHYHTSTALNGRYGHRLVKLWEQPEYTTKPLKVYRTGGRLPTSRFGSDE
Dm            --MQSVTRLLSTTLTIQTPFRTAPAFLQQLRGKTKVVE-------------KPKPGAGQQF----RRTVHFP--EKYTVEPLKITHLAGRDP--------
Ce            --MISSLAVEFGALRLACTSKIVMATRNMPARGTKPLP-------------RHLWDMSDLE----EKTS-----GQYTHKPLRINRLGGRDP--------
Rp_RPL2       ----------------------------------------------------MALKNFNPITPSLRELVQVDKTNLWKGRPLKTLTKGMSKT-----GGR
Ec            ----------------------------------------------------MAVVKCKPTSPGRRHVVKVVNPELHKGKPFAPLLEKNSKS-----GGR
```

Figure S1. Yoshihama *et al.*

```
Hs_RPL8   GSVFRAHVKHRKG--AARLRAVDFAERHG-------YIKGIVKDIIHDPGRGAPLAKVVFRDPYRFKKRTELFIAAEGIHTGQFVYCGKKAQLNIGNVLP
Ol        GSVFKAHVKHRKG--AAKLRHIDFAERNG-------YIKGIVKDIIHDPGRGAPLAKVVFRDPYRFKKRTELFIAAEGIHTGQFIYCGKKAQLNIGNVLP
Ci        GSIFKAHTTHRKG--AAKLRSVDFAERHG-------YIKGIIKDIVHDPGRGAPLVKVQFQDPYKYRRRLETFVAVEGNFTGQSIYCGKKAQLTIGNVLP
Dm        GSVFKAHVKKRKG--AAKLRSLDFAERSG-------YIRGVVKDIIHDPGRGAPLAVVHFRDPYRYKIRKELFIAPEGMHTGQFVYCGRKATLQIGNVMP
Ce        GGIFKSHNKHRKG--ASKLRPLDYAERHG-------YIKGLVKDIIHDPGRGAPLAIIAFRDPYKYKTVKTTVVAAEGMHTGQFIHCGAKAQIQIGNIVP
Sp        GGIFQAHTRLRKG--AAQLRTLDFAERHG-------YIRGVVQKIIHDPGRGAPLAKVAFRNPYHYRTDVETFVATEGMYTGQFVYCGKNAALTVGNVLP
Dd        GSVFGAHTHHRKG--TPRFRALDYAERQG-------YVKGVVKEIIHDPGRGAPLARVVFKGLTQFKLDKQLFIAPEGMHTGQFVFAGKKATLTIGNILP
At        GSVFKSHTHHRKG--PAKFRSLDFGERNG-------YLKGVVTEIIHDPGRGAPLARVTFRHPFRFKKQKELFVAAEGMYTGQFLYCGKKATLVVGNVLP
Pf        GSIFKSHNHHRKG--AAKLRHLDYCEKKG-------YIKGLVKDIIHDPGRGAPLAKVIFKRTEKYGKKEELIIASEGMFTGQYISCGTKAPLSVGNILP
Hs_MRPL2  -HTGRIRVHGIGGGHKQRYRMIDFLRFRPEETKSG-PFEEKVIQVRYDPCRSADIALVAGGSRKRWIIATENMQAGDTILNSNHIGR-MAVAAREGDAHP
Ol        -HTGRVRTHGIGGGHKQKYRMIDFQRLRYEAPDGGRPFEEKVVEVRYDPCRSADIALVAGGNRKRWIIATETMQAGNIIKTSGVIGR-MAVSANEGDAYP
Ci        KVPGKKWTSKIGGGLPRQWYWVDNNRLTFEEVKNEVIFQERILKVMEDDNRSGHIALVAGPLKQRWILATETMKPGQLITNCATELP-GGTEFKDGDAYP
Dm        -VSGRLVAKGIGGGIKQQYRWVKWVRDGP--TEG--AQEELVLEVLRDGCRTAKVALVAVGDELKYILATENMKAGDILKTSRFIPR-IPVRPNEGDAYP
Ce        -ETGRKVNQHIGGGVKFDYFMIDFHRGP--ADQGATYDERILEIRRDPNRTCHIALCAGINGKRWILATENMKAGDVISTSGHLSE-NPVIGVEGNAYP
Rp_RPL2   NQQGRITSWHRGGGHKKLYRVIDFKRKKI-------DISAIVERIEYDPNRTAFIALIKYDD-----GEYSYILAPQKLSIGDRVISSQAADIKIGNCLP
Ec        NNNGRITTRHIGGGHKQAYRIVDFKRNKD-------GIPAVVERLEYDPNRSANIALVLYKD-----GERRYILAPKGLKAGDQIQSGVDAAIKPGNTLP

Hs_RPL8   VGTMPEGTIVCCLEEKPGDRGK-LARASGNYATVISHNPETKKTRVKLPSGSKKVISSANRAVVGVVAGGGRIDKPILKAGRAYHKYKAKRNCWPRVRGV
Ol        VGTMPEGTIICCLEEKPGDRGK-LARASGNYATVISHNPETKKSRVKLPSGAKKVIASANRAVVGVVAGGGRIDKPILKAGRAYHKYKAKRNCWPRVRGV
Ci        VGILPEGTVVCGLEEKTGDRGK-LAKTSGNYATIIAHNPDTNRTRVKLPSGAKKLIPSTNRAIIGVVAGGGRIDKPLLKAGAAYHKYKVKRNSWPKVRGV
Dm        LSQMPEGTIICNLEEKTGDRGR-LARTSGNYATVIAHNQDTKKTRVKLPSGAKKVVPSANRAMVGIVAGGGRIDKPILKAGRAYHKYKVKRNSWPKVRGV
Ce        VGTLPEGTTICNVENKSGDRGV-IARASGNYATVIAHNPDTKKTRIRLPSGAKKVVQSVNRAMIGLVAGGGRTDKPLLKAGRSYHKYKAKRNSWPRVRGV
Sp        VGEMPEGTIISNVEEKAGDRGA-LGRSSGNYVIIVGHDVDTGKTRVKLPSGAKKVVPSSARGVVGIVAGGGRIDKPLLKAGRAFHKYRVKRNCWPRTRGV
Dd        IGKLPEGTIICNVEEKLGDCGA-VARCSGNYATIVSHNPDEGVTRIKLPSGSKKNVSSLARAMIGIVAGGGRIDKPMLKAGRAFHKYRVKKNNWPKVRGV
At        LRSIPEGAVVCNVEHHVGDRGV-LARASGDYAIVIAHNPDSDTTRIKLPSGSKKIVPSGCRAMIGQVAGGGRTEKPMLKAGNAYHKYRVKRNSWPKVRGV
Pf        IGKMPEGTLICNLEHRTGNRGT-LVKASGCYATVVGQSEDGKKTKVRLPSGAKKTIDAKARAMVGVVGAGGRIDKPILKAGVAHHKYRVKRNCWPKVRGV
Hs_MRPL2  LGALPVGTLINNVESEPGRGAQ-YIRAAGTCGVLLRK--VNGTAIIQLPSKRQMQVLETCVATVGRVSNVDHNKRVIGKAGRN-------RWLGKRPNSG
Ol        LGALPVGTLVNNLEVQPGRGAE-YIRAAGTSGVLLRK--VNGTAIVQLPSKQQVQVLETCMVTVGRVSNVDHNKRVIGKAGRN-------RWLGIRPSSG
Ci        VGLIPVGTKICSLEFRPGKGAQ-IARSAGAYCTVSRK--TNTNTVVQMPSKREIMISSSCVAVIGRVSNVGHDDVKWGSPQRL-------REYGLRPKSG
Dm        LGALPVGTRIHCLEKNPGQMCH-LIHAAGTFGTILRK--FDEKVVVQLPSKREFAFQRTCMATVGRLSNPEHNKEHVGSAQRM-------REMGNRPRSG
Ce        IGSLAAGTVINSIERYPTMDSETFVKAAGTSATIVRH--QGDFTVVKLPHKHEFSLHRTCMATVGRLSHADIDGKIFGSAQMH-------RRFGYKMSSG
Rp_RPL2   LKSIPIGTTLHNVEMKIGKGGQ-IARSAGTSVELVGK--DSGYAQIKLRSGEFRLVPLDCKATIGSISNPDQKNINLGKAGRNRWLG-----WRPHVRGV
Ec        MRNIPVGSTVHNVEMKPGKGGQ-LARSAGTYVQIVAR--DGAYVTLRLRSGEMRKVEADCRATLGEVGNAEHMLRVLGKAGAARWRG-----VRPTVRGT

Hs_RPL8   AMNPVEHPFGGGNHQHIGKPSTIRRDAPAGRKVGLIAARRTGRLRGTKTVQ-EKEN-----------
Ol        AMNPVEHPFGGGNHQHIGKPSTIRRDAPAGRKVGLIAARRTGRLRGTKTVQ-EKEN-----------
Ci        AMNPVEHPFGGGNHQHIGSPSTVRRDASPGRKVGLIAARRTGRLRGSRSVK-DKE------------
Dm        AMNPVEHPHGGGNHQHIGKASTVKRGTSAGRKVGLIAARRTGRIRGGKGDSKDK-------------
Ce        AMNPVEHPHGGGNHQHIGHPSTVRRDASAGKKVGLIAARRTGRIRGGKPVKFTKEENV---------
Sp        AMNPVDHPHGGGNHQHVGHSTTVPRQSAPGQKVGLIAARRTGLLRGAAAVEN---------------
Dd        AMNPVEHPHGGGNHQHVGHATTTKRDDPAGKKVGLIAARRTGRLRGTKNISE---------------
At        AMNPVEHPHGGGNHQHIGHASTVRRDAPPGQKVGLIAARRTGRLRGQAAASAAKAD-----------
Pf        AMNPVEHPHGGGNHQHIGHPSTVSRSAPAGQKVGLIAARRTGLLRGAKKTKLVGKSEE---------
Hs_MRPL2  R-WHRKGGWAGRKIRPLPPMKSYVKLPSASAQS----------------------------------
Ol        L-WQRKGGWAGRKIKPLPPMKSYVNRPSISAA-----------------------------------
Ci        RPNIKRDGRFGRKIKPPKHTIVIGDKALHQRTTDFNE------------------------------
Dm        L-WKRKEGKHGRKIRRLPPMTTISPP--APPKEEAIKLT----------------LPL---------
Ce        L-FHKKDGYFGRKVRALPPVRILDTPPQAPPPNQRFTLSKKDLSGLHGHAKVHNLIPAGYSTRDYPE
Rp_RPL2   AMNPVDHPHGGGEGKTSGGRHPVTPWGFSTKGKKTRKNKRTSKFIVKKRK-----------------
Ec        AMNPVDHPHGGGEGRNFG-KHPVTPWGVQTKGKKTRSNKRTDKFIVRRRSK----------------

Hs_RPL10  ---------------------------------MGRRPARCYRYCKNKPYP-----KSRFCRGVPDAK--IRIFDLGRKKAKVDEFPLCGHMVSDEYEQ
Ol        ---------------------------------MGRRPARCYRYCKNKPYP-----KSRFCRGVPDPK--IRIFDLGRKKAKVDEFPLCGHMVFDEYEQ
Ci        ---------------------------------MGRRPARCYRYCKNKPYP-----KSRFCRGVPDPK--IRIFDLGRKRADVRDFPLCMHMVSDELEQ
Dm        ---------------------------------MGRRPARCYRYIKNKPYP-----KSRFCRGVPDPK--IRIFDLGRKKATVEDFPLCVHLVSDEYEQ
Ce        ---------------------------------MGRRPARCYRYIKNKPYP-----KSRFCRGVPDAK--IRIFDLGNKRANVDTFPACVHMMSNEREH
Sp        ---------------------------------MARRPARCYRYCKNKPYP-----KSRYNRAVPDSK--IRIFDLGRKRAGVDEFPLCIHLVSNEYEQ
Dd        ---------------------------------MGRRPARCYRYCKNKPYI-----KSRYCRGVPDAK--IRIFDLGRKKASTDEFPLCVHLISLEKEQ
At        ---------------------------------MGRRPARCYRQIKGKPYP-----KSRYCRGVPDPK--IRIYDVGMKRKGVDEFPFCVHLVSWEKEN
Pf        ---------------------------------MGRRPARCYRYCKNKPYP-----KSRYCRGVPDPK--IRIYDMGRKKADVNEFSGVVHLVSYEYEQ
Hs_MRPL16 MWRLLARASAPLLRVPLSDSWA-------LLPASAGVKTLLPVPSFEDVSIP-EKP-KLRFIERAPLVP---KVRREPKNLSDIRG-PST-EATEFTEGN
Ol        MVSVLKAAVGGLMDVCRANNHLRGFLHNHLKLFAAGMKTYEIPPDYSNVVLP-AKP-KLKFLNKVPNLK---KAKKEMKKLRDIQG-PAK-AANAFTVGQ
Ci        ----------------------------------------------MYENIDVP-ETERALPLVAKQPDVP----PKDRRKMYLIRG-CCFGEDAQLKYGQ
Dm        --MLSLKLVSQLFKQSLASGNM-------AIVNTAGLKYFAPPIKYQNVEQP-ERP-KLRVIERQPQLPPNIRPPKMQKRLRYMRG-PEM-VHNTLLHKQ
Ce        ---------------------------------MKKYSFPVTYENVRFPPNGQLKLPVMPAEPFYDAEKGEKKYKSTKRMIEARGVEEVHTELIHEQ
Rp_RPL16  ----------------------------------------------MLAP--------------------KKQKFRKAHKGRVMSKAKAGMTLAFGS
Ec        ----------------------------------------------MLQP--------------------KRTKFRKMHKGRNRGLAQG-TDVSFGS

Hs_RPL10  LSSEALEAARICANKYMVKSCGKDGFHIRVRLHPFHVIRINKMLSCAGADRLQTGMRGAFGKPQGTVARVHIGQVIMSIRTKLQNKEHVIEALRRAKFKF
Ol        LSS-------------------------------------------------------------------------------------------------
Ci        LSSEALEAARICANKYMVKYCGKDSFHLRVRVHPFHVVRINKMLSCAGADRLQTGMRGAYGKPQGLVARVKIGQPIMSMRCKDSARAHAIEALRRAKFKF
Dm        LSSEALEAGRICCNKYLVKYCGKDQFHIRMRLHPFHVIRINKMLSCAGADRLQTGMRGAFGKPQGTVARVRIGQPIMSVRSSDRYKAQVIEALRRAKFKF
Ce        LSSEALEAARICANKYMVKNCGKDGFHLRVRKHPFHVTRINKMLSCAGADRLQTGMRGAYGKPQGLVARVDIGDILFSMRIKEGNVKHAIEAFRRAKFKF
Sp        ITSEALEAARICANKYLVKIGGKDSFHLRVRAHPFHVVRINKMLSCAGADRLQTGMRHAFGKPNGLVARVNIGQILMSVRTKDSSRATAIEALRRCQYKF
Dd        LSSEAIEAGRISCNKYISKTGGKDSFHMRVRVHPWHVLRINKMLSCAGADRLQTGMRGAFGKPMGTVARVNIGQIIFSIRTRDNMLANVVEALRSSYKF
At        VSSEALEAARIACNKYMVKSAGKDAFHLRIRVHPFHVLRINKMLSCAGADRLQTGMRGAFGKALGTCARVAIGQVLLSVRCKDNHGVHAQEALRRAKFKF
Pf        ISSEALEAARISANKYMITNCGKDNFHLRVRIHPFHVLRINKMLSCAGADRLQTGMRGAFGKPNGVVARVDIGQVLLSIRTKENFVSKACEALRRAKYKF
Hs_MRPL16 FAILALGGGYLHWGHFEMMRLTIN--RSMDPKNMFAIWRVPAPFKPITRKSVGHRMGGGKGAIDHYVTPVKAGRLVVEMGGR-CEFEEVQGFLDQVAHKL
Ol        YAIVAMGGGYLHWGHFEMMRLTIN--RKMDARTTFARWRVNAPYKPVTRKGLGQRMGGGKGAIDHYVTPVRYGRLIVELGGK-VELGEVESVLTEVAKKL
Ci        YGVVALSGGFFKIGNFNFMMQRVN--KFIKKKPLFAKWRIPAPYHAVTKHPLQAVMGGGKGKIDHYVTPVKSRQIILEIGGK-AEFTEIYPLLNSLAVVM
Dm        YAIVATGGGRLRWGHYEMMRLTIG--RKMNVNTMFATWRVPAPWQPITKKGQGQRMGGGKGAIDHYVTPIKAGRVIVEIAGK-CEFVEVKQFLQQVANQL
Ce        YGLAAVSGGFIDSDDFKFIQDRIN--KNLIDN-QFSVWRVDAPWLPRTKKAQGTRLGGGKGSIQKYVTPVRANRIILEVGGH-ITEIEAKAFLLYLCERF
Rp_RPL16  FGLKSIDGWRVTARQIEAGRKAAT--RCMKRQ--GRLWIRIFPDVPVSKKPAEVRMGKGKGTPEFFAVRVSPGRIMFEIEG--VEENIALRALELASAKL
Ec        FGLKAVGRGRLTARQIEAARRAMT--RAVKRQ--GKIWIRVFPDKPITEKPLAVRMGKGKGNVEYWVALIQPGKVLYEMDG--VPEELAREAFKLAAAKL

Hs_RPL10  PGRQKIHISKKWGFTKFNADEFEDMVAEKRLIPDGCGVKYIPSRG----PLDKWRALHS-------------
Ol        ------------------------------------------------------------------------
Ci        PGRQKIYDSPKWGFTKWNREDYERMLAEGLLIPDGVSCQYKPNKG----KLDLWKKRQ--------------
Dm        PGRQKIYVSKKWGFTKYEREYEELRDDNRLEPDGCNVKYRPEHG----PIAAWEKAQRDVYA----------
Ce        PGRQIIVSSRKWGFTKWDREDYERMRAEGRLRSDGVGVQLQREHG----PLTKWIENPI-------------
Sp        PGQQRIIVSKKWGFSQYARDEYIEKRSRGEIIPDGCYAKFLNKRGSLQEKLDLFPEASFNLA----------
Dd        PGRQKIVVSKKWGFTAYNREAYQKLKADGRLMNDGANVKVITNHG----TLAQYAKDIAAAN----------
At        PGRQKIIVSRKWGFTKFNRAEYTKLRAMKRIVPDGVNAKFLSNHG----PLANRQPGSAFISATSE------
Pf        PGRQKVFVSNKWGFTPFSKDEYQQYKKKGRIISDGVSCKFIREKG----PLDKIYKDINTVLES--------
Hs_MRPL16 PFAAKAVSRGTLEKMRKDQEERERNNQNPWTFERIATANMLGIRK----VLSPYDLTHKGKYWGKFYMPKRV
Ol        PFPAKVMSREDLEALKKVHADMEQNNQNPWTFKQIVQGNMLGIRK----VLSPFDLHNHGRFTGKFHLPGRV
Ci        PVKALPVDAEMLQAMYDEERRIEEENENFFTFREVIEKNMQGIRS----KMSRDFKHYG-------------
Dm        PFQATVVSQEMLDEQRVAEEEQTRQNENPFTMKYVIQNNLSGCHR----WLSPVDHKWFGKHL---------
Ce        SFPVEFVSETMLAERRKKAEEERAMNKNGFNWGKVIKYNMQNCNS----WLSSYDVAWKGKYR---------
Rp_RPL16  PVRTRIVRRYE-------------------------------------------------------------
Ec        PIKTTFVTKTVM------------------------------------------------------------
```

Figure S1. Yoshihama *et al.*

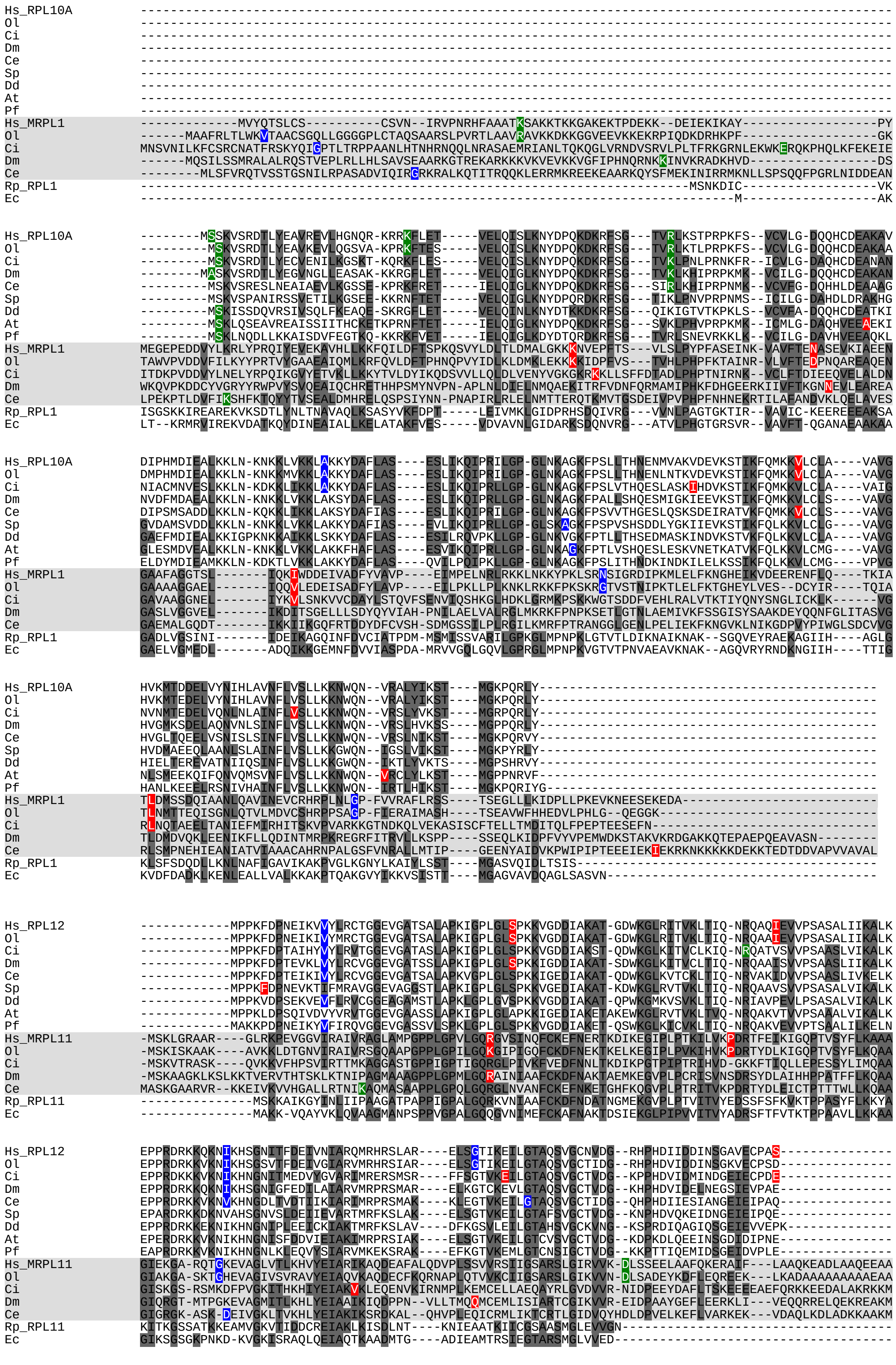

Figure S1. Yoshihama *et al.*

```
Hs_RPL12           -----
Ol                 -----
Ci                 -----
Dm                 -----
Ce                 -----
Sp                 -----
Dd                 -----
At                 -----
Pf                 -----
Hs_MRPL11          KK---
Ol                 SSKKK
Ci                 IGKE-
Dm                 LRTG-
Ce                 LRTT-
Rp_RPL11           -----
Ec                 -----


Hs_RPL13A          ------------MAE-----VQVLVLDGRGHLLGRLAAIVAKQVLL------------GRKVVVVRCEGINISG---NFYRNKLKYLAFLRKRMNTNPSR
Ol                 ------------MADR---FNKVLLIDGRGHLLGRLAAIVAKQVLL------------GHKVVIVRCEGINISG---NFYRNKLKYLAFLRKRMNTNPSR
Ci                 ------------MADG---FVKVRIIDAKDHLLGRLAALIAKNLLN------------GQKIVVVRCESILISG---NFYRNKLKYLDFLRKRIRTNPKR
Dm                 ------------MTGLTN---RTVVIDGRGHLLGRLASVVAKYLLQ------------GGKVAVVRCEELNLSG---HFYRNKIKFLAYLRKRCNVNPAR
Ce                 -------------MGLSN---RAIIIDGKNHLLGRLASIVAKKLLQ------------GDKVVVLRAEEIVISG---NFHRSKLKYMSFLRKRCNINPAR
Sp                 ------------MSEFQ----KLVIIDAKGHLMGRLASTVAKQLLA------------GQKVVVVRCEELNISG---HFFRNKLKYLAYLRKACRYNPSR
Dd                 -----------MFQK------KAIVIDGKGHLLGRLASVVAKSLLS------------GQKIVVVRCEELNISG---PLSRNKLKWADFLNLTMNTNHAR
At                 ------------MVSGSGICSKRVVVDARHHMLGRLASITAKELLN------------GQKVVIVRCEEICLSG---GLVRQKMKYMRFLRKRMNTKPSH
Pf                 ------------MYK------KEYVIDCKGHLLGRLASLIAKELLN------------GQRIVAVRCEDINISG---SLYRNKLKYQEFLRLRTNTNPKK
Hs_MRPL13          -----MSSFSRAPQQWATFARIWYLLDGKMQPPGKLAAMASIRLQGLHKPVYHALSDCGDHVVIMNTRHIAFSG---NKWEQKVYSSHTGYPGGFRQVTA
Ol                 -----MSSFSRSAQQWATFARSWFLIDARMQPPGKIASMCSVRLQGKHKPIYHALSDCGDHVVVINSRQIAFSG---NKWEQKVYSSHTGYPGGFKQVTA
Ci                 ------MSFGRANQQWSTFSKAWYLLNADRQPPGKLADVAAKYLMGMHKPIYHPTSRVGDHVVIINAKEIAYSG---NKWEEKTFHSHTGRKKGRLMMRA
Dm                 ------MSIAKRVQQWATFARTWHIYDCTWQNPFESAKLVKTHLLGLQKPIYHPMNDCGDHVVLINTREIALPG---DEWVKRVYFHHTGYPGGASWTLA
Ce                 MSYRRAVSRFDRVNQWLQFSRQWHVIDANQQDAELLGDKVARYLAGKHKPIFHPETDCGDHVVVTNCKDIAMHA---FDWKHTIYKFNMEYPKSKADIPA
Rp_RPL13           --------MKTYSAKPSEIEKKWWVIDAKNVVLGRLASRVAIMLRGKHKPSFTPHLDCGDNIIIINAEHIKLTGKKLNPKDGKVYYRHTGFPGGIKDTTA
Ec                 --------MKTFTAKPETVKRDWYVVDATGKTLGRLATELARRLRGKHKAEYTPHVDTGDYIIVLNADKVAVTG---NKRTDKVYYHHTGHIGGIKQATF

Hs_RPL13A          GPYHFRA-PSRIFWRTVRGMLPHKT---KRGQAALDRLKVFDGIPPPYDKKKRMVVPAALKVVRLKPTRKFAYLGRLAHEVGWKYQAVTATLEEKRKEKA
Ol                 GPYHFRA-PSRIFWRTVRGMLPHKT---KRGQAALERLKVFDGIPPPYDKRKRVVVPAALKIVRLKPSRKFALLGRLAHEVGWKYQAITATLEEKRKEKA
Ci                 GPFHFRA-PSKILWRTVRGMLPHKL---KRGAEALDRLKVFEGVPPPYDKKKRMVVPSCMKVVKLKPKRKFALLSRLSSEVGWKYNDVIQALEEKRKAKS
Dm                 GPFHFRA-PSRIFYKAVRGMIPHKT---KRGQAALARLRVFDGIPSPYDKRRRVVVPIAMRVLTLRSDRKYCQVGRLSHEVGWHYQDVIKSLERKRKAKL
Ce                 GAFHYRA-PGKIFWRTVRGMLPHKT---NRGNEALKNLRAYEGVPAKYQKTKSLHAPSASR-FRLQPRRKFCVVGRLSHEVGWQFQDVVAKLEAKRKVKG
Sp                 GAFHFRA-PSRIFTKAVRGMLPHKT---TRGNIALKNLQALEGIPPPFDKQKRLVVPAALRVLRLKPSRKYCTIGRLSSEVGWKYKNIVSKLEERRKIKS
Dd                 GHRHGRS-PSKIFWRAVRGMLPHKT---PRGQAALDNMKVFEGVPAPYDKVKRVVVPSALRVVKLNTTRKYTVLSRLSQEVGWKYRAVVAKLEVQRKQRS
At                 GPIHFRA-PSKIFWRTVRGMIPHKT---KRGAAALARLKVYEGVPTPYDKIKRMVIPDALKVLRLQAGHKYCLLGRLSSEVGWNHYDTIKELETKRKERA
Pf                 GPLHLRE-PSKILWRCVRGMLPHKT---YKGQLALKKLKVCVGMPYPYDKKKKYVLPSALRAFRLKKHRRYCRLGTLSSRVGWNYDTLVKKNEVLRKQVS
Hs_MRPL13          AQLHLRD-PVAIVKLAIYGMLPKN----LHRRTMMERLHLFPDEYIPEDILKNLVEELPQPRKIPKRLDEYTQEEIDAFPRLWTPPEDYRL---------
Ol                 AQLHHKD-PKAIVKLAVYGMLPRN----LHRRTMMQRLHIFSDDALPDDIRANLTEELQQPRAIPRKLSEYTPEEVEAFPRLWTPPEDYKMK--------
Ci                 FEVHELD-PTWIVKRYVYRRLPKGSYGGMYKRNLFQKLHVFADENIPEEIKENISFVIPQPRRVFKGINDYTQEEIDNFPKLWIPSPDFLKR--------
Dm                 WQLHEKD-PTMVMKKAVYNSMRGN----LQRRHTMQRLHLFADDQVPEEILQNVTNQIRTPRSIPQRLDHIDKETLENFPNIMDYPKDYILR--------
Ce                 WQIHDYD-QCRIAFLSVYRSLGNN----LLRRRHIQRLHLFPDEEMAEFVRKNIGSQLRQVQGVVKRSDEYTAEERANFPRVVKFADKHVVDWEKSIPNP
Rp_RPL13           GKILSGKYPERVIKMAVKRMITRN----VLGAKQMSNLYVYANCEHPHMAQQPTIYDFASENPKNKK---------------------------------
Ec                 EEMIARR-PERVIEIAVKGMLPKG----PLGRAMFRKLKVYAGNEHNHAAQQPQVLDI------------------------------------------

Hs_RPL13A          KIHYRKKKQLMRLRKQAEKNVEKKIDKYT-EVLKTHGLLV-
Ol                 KLHYVKKKTLVKLSKLAEKNVEGKIAKYT-DVLKQYGVLV-
Ci                 KLYWDKKQSNLKIRKEAIANVQDQIADLD-KQIQDMGCYAV
Dm                 RVTLKHNRELKKLTVKARENIAKAAEPFN-KIIKSYGYEV-
Ce                 AAYFEQKKKMDKLAVQAKKNAAPKIAQYQ-KIIEALGYN--
Sp                 AAFYQA-----KSANQKHINVAKTKSSVN-EKLAVFGY---
Dd                 STYYRKAA---------------LVKAYRTQALKKFSA---
At                 HVVYERKKQLNKLRVKAEKVAEEKLGAQL-DILAPVKY---
Pf                 KAYYKKKVNNLNEKKEIKTEALNLINPEQRQVLENFGYA--
Hs_MRPL13          -----------------------------------------
Ol                 -----------------------------------------
Ci                 -----------------------------------------
Dm                 -----------------------------------------
Ce                 GRHVKPVPGQKDK----------------------------
Rp_RPL13           -----------------------------------------
Ec                 -----------------------------------------


Hs_RPL17           -----------------------------------------------MVRYSLDPENPTKSCKSRGSNLR--------------------VHFKNTRETA
Ol                 -----------------------------------------------MVRYSLDPENPTKSCKARGSNLR--------------------VHFKNTRETA
Ci                 -----------------------------------------------MTRYSQDPENPAKSCKARGSDLR--------------------VHFKNTRETA
Dm                 -----------------------------------------------MGRYSRESDNVAKSCKARGPNLR--------------------VHFKNTHETA
Ce                 ---------------------------------------------MTKVHYSRAPENSTKSCKARGSDLR--------------------VHFKNTHEAA
Sp                 -----------------------------------------------MVRYSASPALETKCAKARGAYLR--------------------THFKNSREVA
Dd                 ---------------------------------------------MTKPVYSKTPSNPEKSCKSRGSNLR--------------------IHFKNTRESA
At                 -----------------------------------------------MVKYSQEPDNQTKSCKARGSDLR--------------------VHFKNTRETA
Pf                 -----------------------------------------------MVKYAKKIRNLGKCAKGAGVDLR--------------------VHFKNTYEAA
Hs_MRPL22          -------------------------MAAAVLGQLGALWIHNLR---SRGKLALGVLPQSYIHTSASLDI--------------------SRKWEKKNKIV
Ol                 -------------------------MAAAGAGS-GFSFVRSLSGLFRSSRLAAPPQRLSWLHTGSSLS---------------------SKSWERKNLKV
Ci                 --------------------------------------------------------------------------------------------WTENNKML
Dm                 --------------MHKVIRQMSQLRLQAPQGAALLRSADSSSISPAISPVSPPALQSKSLHTAASAGMLCAKWNKY---------NYGPRKWLEYNKTV
Ce                 MLRVLVRTGSEACRAVSTSTAVLSVHPVVKSEDLSAKEIWDRRSKLRVPKLQKDEQLSSKVYFAPEWDLEKKPNLDEGFVNPLKGYGMTPEKWEYYNKVV
Rp_RPL22           -----------------------------------------------------MIQKNKNFATAKAKSIR--------------------VSPRKLNLVA
Ec                 ------------------------------------------------------MET-----IAKHRHAR--------------------SSAQKVRLVA

Hs_RPL17           QAIKGMHIRK---ATKYLKDVTLQKQCVPFRRYNGGVGRCAQAKQWGWT-QGRWPKKSAEFLLHMLKNAESNAEL-KGLDVDS-LVIEHIQVNKAPKMRR
Ol                 QAIKGMHIRK---ANKYLRDVVVKRQCVPFRRYNGGVGRCAQAKQFDWT-QGRWPKKSAEFLLHMLKNAESNAEL-KGLDVDS-LVIEHIQVNKAPKMRR
Ci                 QAIKGMHIRK---ATQYLKDVCEKKRCIPFRRFNGGVGRCAQAKEFKTT-QGRWPKKSATFLLQMLKTAESNADV-KGLDVDS-LVIDHIQVNAAAKMRR
Dm                 QAIKRMPLRR---AQRYLKAVIDQKECVPFRRFNGGVGRCAQAKQWKTT-QGRWPKKSAEFLLQLLRNAEANADC-KGLDADR-LVVHHIQVNRAQCLRR
Ce                 MALRGMPLRR---AQAFLNHVKEHKEIVPFRRFHGGIGRAAQTKQWNTT-QGRWPVKSADFLLDLLKNAESNAEY-KGLDVDH-LVIEHINVQRAAKLRR
Sp                 FTINGMSLKK---AFIFLDNVKEHKQAVPFRRFNGGVGRTAQGKEFGVT-QARWPVKSVKFFYDLLKNAEANAEA-KGLDMDK-LIIKHVQVNAAPKQRR
Dd                 MAIKGMLLTR---AKAYLNNVLAHRECIPFRRFKGGVGRTGQAKIFGTS-QGRWPKKSVEHILSLLQNAEANAEA-KGLNVEK-LKIAHVQVQRAQQQRR
At                 HAIRKLPLIK---AKRYLEDVIAHKQAIPFTRFCRGVGRTAQAKNRHSNGQGRWPAKSAQFVLDLLKNAESNAEV-KGLDVDA-LFISHIQVNQAAKQRR
Pf                 RAIRRMKLLE---AKKYLNDVIEKKRCVPFRKYNGGVGRTNQAKEFNHT-QGRWPAKSCKFLLNILDNVQANAES-RNLDVSK-LRLIHVMVNKARPGRR
Hs_MRPL22          YPPQLPGEPR---RPAEIYHCRRQIKYSKDKMWYLAKLIRGMSIDQALAQLEFNDKKGAKIIKEVLLEAQDMAVRDHNVEFRSNLYIAESTSGRGQCLKR
Ol                 FPPQQPEEPR---RPAEIHHCRRQIKYSKDKMWYLAKMIRGMSIDEAVAQLEFNDKKGAKIMKEVLLEAQEMAVKTHNVEYKSNLHVAESFSSKGKYLKR
Ci                 YSPMDPEKGV---RPAEIYYGRANVRYPPKKLYHVAYIVKGLNIDEAITHLGYINNKAARTIADVLREAQEHAVAEHNVEFKSNLHIVDSFAESSGAVKF
Dm                 HPPQETDEEP---RNAYVCHMRSNIKYSPDKMWYIAAFVRGMSVDEALKQLNFVLKKGATDVKETILEAQQIAVERHNVEYKSNLWIAESFVGKGRVFKG
Ce                 WPPNYIVPETGLPKPKEVFHCKESVHFSPKRMWAACQLVWKMNVDEAITQLDMQQLKACNLLMDTIKKAKSRAADEFHIEYPSQMYVADAFPVQSNIVKG
Rp_RPL22           SFIRNMKVSE---ALVQLTFSP---------------------------------KRIAKIVKDCLQSAIANAENNLGLDIDR-LIITKATVGKSVVMKR
Ec                 DLIRGKKVSQ---ALDILTYTN---------------------------------KKAAVLVKKVLESAIANAEHNDGADIDD-LKVTKIFVDEGPSMKR
```

Figure S1. Yoshihama *et al.*

```
Hs_RPL17    RTYRAHGRINPYMSSPCHIEMILTEKEQIVPK-----PEEEVAQKKKISQKKLKKQK--LMARE------------
Ol          RTYRAHGRINPYMSSPCHIEMILTEKEQIVPK-----PEEEVAQKKKVSQKKLKKQK--LMARE------------
Ci          RTYRAHGRINPYMSSPCHIEMILTEKEQIVPR-----VEEEQK-KKKVSKKKIARQK--LMGKE------------
Dm          RTYRAHGRINPYMSSPCHVEVILTEKEELVSK-----ATDDEPAKKKLSKKKLQRQKEKMLRSE------------
Ce          RTYRAHGRINPYMSSPCHIEVILAEKEDVVSK-----PTDDAAPKVKKESKRKQRRQ--LARGEF-----------
Sp          RTYRAHGRVTAYLSSPSHIEIIVAEEEEAVPKAND--TVSRVSLKQGAKARNLAARKAITAA--------------
Dd          RTYRAHGRINPYMCSPSTVEFILTEVEKAVPKP----AEESAQKKKSVATQEISA---------------------
At          RTYRAHGRINPYMSNPCHIELILSEKEEPVKKE----PETQLAAKSKKSAA-------------------------
Pf          RTYKAHGRINPFMSSPCHIQIIAREINKPAKKSLLSNAEKQKKLPFKVTLKKLIKLNISNQNRLKNKSVKKQQTVQ
Hs_MRPL22   IRYHGRGRFGIMEKVYCHYFVKLVEGPPPP-------PEPPKTAVAHAKEYIQQLRSRTIVHTL------------
Ol          IRYHGRGMFGIMDRVYCHYFVKLVEGPPPR-------TEQ-KTGLDQKAEYVQMLKDRTVIHSL------------
Ci          PIYHARGRMALGKCRFSNYYCLLREGPAPP-------KRPKLTAL-------------------------------
Dm          VRRHARGRFGKVEYKHCHYFVRLEEGEPPQHY-----YQEPQTPEQQYESWMEQMRSRKIINSL------------
Ce          ARRHAHDNWNTIRYRYIHIFVRLEEGPAPQQKQ---RHPQKNGWDHMDEYYNYLRSRTVKYSI------------
Rp_RPL22    IMPRAKGRATRINKFFSNLYITVTEKEDN-----------------------------------------------
Ec          IMPRAKGRADRILKRTSHITVVVSDR--------------------------------------------------
```

```
Hs_RPL23    -------------MSKRGRGGSSGAKFRISLGLPVGAVIN--------------------CADNTGAKNLYIISVKGIKGRLNRLPAAGVGDMVMATV
Ol          -------------MSKRGRGGSSGAKFRISLGLPVGAVIN--------------------CADNTGAKNLYIISVKGIKGRLNRLPAAGVGDMVMATV
Ci          -------------MSKRGRGGAAGGKFRISLSLPVGAVVN--------------------CADNTGAKNLYIIAVKGIRGRLNRLPAAGVGDIVLATV
Dm          -------------MSKRGRGGTAGGKFRISLGLPVGAVMN--------------------CADNTGAKNLYVIAVHGIRGRLNRLPAAGVGDMFVATV
Ce          -------------MSKRGRGGASGAKFRISLGLPVGAVMN--------------------CADNTGAKNLFVISVYGIRGRLNRLPSAGVGDMFVCSV
Sp          -------------MSR-GRGAASGTKYRMTLGLPVQAIMN--------------------CADNSGAKNLYIVSVFGTGARLNRLPAASCGDMVLATV
Dd          -------------MSK---AQAVGSNYRVSLGLPVGAVMN--------------------SADNSGAKNLYVIAVKGIKGRLNRLPSAGVGDMVMATV
At          -------------MSKRGRGGTSGNKFRMSLGLPVAATVN--------------------CADNTGAKNLYIISVKGIKGRLNRLPSACVGDMVMATV
Pf          --------------MKRGRAGTLKNKMRITLSLPVGALIN--------------------CCDNSGGKNLYIIAVQGFGSCLNRLPAASLGDMVLATV
Hs_MRPL14   -----------MAFFTGLWGPFT----CVSRVLSHHCFST--------------------TGSLSAIQKMTRVRVVDNSALGNSPYH--RAPRCIHVY
Ol          -----------MALQR-LSGSFSGLIVQNSSKILQRAFSV--------------------SVVAAAIQKMTRVRVVDNSSLGNTPYH--RPPRVIHVY
Ci          MLSTNILKLTALNQLQKFCFGTTTQRFLPDSRNYDNQCFYDGVQFGYKATKSNIRNRDRQVKKMTKRAFQKQTRVRIVDNTTYALSPVL--RAARVIQVY
Dm          ----------MALRILKTVRQVAQPLAVTLGGQQTQQLIHT--------------------TPACCEIRKLARLRVVDNSDLGKKAMAEGRPPRCIHVY
Ce          -MANLPSSSGMFSRLLANVFNTTVASRCSASGRWWPQPEYELSKHR---------------SRAPSMGIHRRTRLNVVDNSALGKEADTTGKLPYCIHVY
Rp_RPL14    -------------------------------MIQMQSILE--------------------VADNSGAKKVMCIKVLGGSHHM----MAKLGDVIVVSI
Ec          -------------------------------MIQEQTMLN--------------------VADNSGARRVMCIKVLGGSHRR----YAGVGDIIKITI
```

```
Hs_RPL23    KKGKPE-------LRKKVHPAVVIRQRKSYRRK--------DGVFLYFEDNAGVIVNNKGE-MKGSAITGPVAKEC-----------ADLWPRIASNAGS
Ol          KKGKPE-------LRKKVHPAVVIRQRKSYRRK--------DGVFLYFEDNAGVIVNNKGE-MKGSAITGPVAKEC-----------ADLWPRIASNAGS
Ci          KKGKPE-------LRKKVHPAVIIRQSKSYRRK--------HGQMIYFEDNAGVIVNQKGE-MKGSAITGPVAKEC-----------ADCWPRIASNASS
Dm          KKGKPE-------LRKKVMPAVVIRQRKPFRRR--------DGVFIYFEDNAGVIVNNKGE-MKGSAITGPVAKEC-----------ADLWPRIASNASS
Ce          KKGKPE-------LRKKVLQGVVIRQRKQFRRK--------DGTFIYFEDNAGVIVNNKGE-MKGSAITGPVAKEC-----------ADLWPRIAANAGS
Sp          KKGKPD-------LRKKIMPAIVVRQRKAWRRK--------DGVYLYFEDNAGVIVNPKGE-MKGSAITGPVAKEC-----------ADLWPRIASNAGT
Dd          KKGKPE-------LRKKVCTGLVVRQRKHWKRK--------DGVYIYFEDNAGVMCNPKGE-VKG-NILGPVAKEC-----------SDLWPKVATNAGT
At          KKGKPD-------LRKKVLPAVIVRQRKPWRRK--------DGVFMYFEDNAGVIVNPKGE-MKGSAITGPIGKEC-----------ADLWPRIASAANA
Pf          KKGKPD-------LRKKVLNAIICRQSKAWRRH--------EGYYIYFEDNAGVIVNPKGE-MKGSAITGPVAREC-----------AELWPKLSSAASA
Hs_MRPL14   KKNGVG--K----VGDQILLAIKGQKKKALIVGH---CMPGPRMTPRFDSNNVVLIEDNGN-PVGTRIKTPIPTSLR--------KREGEYSKVLAIAQN
Ol          TKNGVG--K----VGDKVLLAIKGQKKKALIVGH---KMPGDRMTPRFDSNNVVLIEDNGN-PTGTRIKVPIPTHLR--------QMEGDFSKVLAIASS
Ci          NKSGHG--S----VGDQVLITVNGAMKRALIVGQ---RRPMGVMKPRTDSNNVIILDNDGN-PEGTRITVPLPSWLRG--YDPKTKRQLALSKILALATT
Dm          NKRGVG--F----IGDKVLVAIKGQMKKGILVGL---KQNQKPKQPKFDSNNLVLIDDNGS-PLGTRIHVPIPTILRTILKEKTLAKGADYTKVLAIASR
Ce          KQGRRAKHMPHAELGDKILVAIRGQMRKAYVVGANTHVHYRKHGVPVTDQNMIVLLDEEGN-PLGNRITAPIPTKLMAN------RGNVQFSKVLALANK
Rp_RPL14    KEAIPGGKV----KKGDVYKGVIVRTKTGVVRS--------DGSTIKFDKNALVLLNKQDE-PIGTRVFGPVTRELR----------AKKYVRIMSLAEE
Ec          KEAIPRGKV----KKGDVLKAVVVRTKKGVRRP--------DGSVIRFDGNACVLLNNNSEQPIGTRIFGPVTRELR----------SEKFMKIISLAPE
```

```
Hs_RPL23    IA
Ol          IA
Ci          IA
Dm          IA
Ce          IA
Sp          VV
Dd          IV
At          IV
Pf          IV
Hs_MRPL14   FV
Ol          FV
Ci          FV
Dm          YV
Ce          FI
Rp_RPL14    VL
Ec          VL
```

```
Hs_RPL23A   -------------------MAPKAKKEAPAP------------PKAEAKAKALKAKKAVLKG-VHSHKKKIRTSPTFRRPKTLRLRRQPKYPRKSAPR
Ol          -------------------MAPKAKKEA-VP------------AKTEAKSKALKAKKAVLKG-VHSHKKKIRTSPTFRRPKTLRLRRQPKYPRKSAPR
Ci          -------------------MAVGG-----------------------AKEKALRAKKAVLKG-LHSHRKKFRKSVHFRLPKTLKLPRAPKYPRKSVAK
Dm          -------------------MPPKKPTEK----------------SATAGAAAKKPAAIIKG-AFGTRARKIRTNVHFRRPTTLKLPRSPKYPRKSVPT
Ce          -------------------MA-------P--------------SAPAKTAKALDAKKKVVKG-KRTTHRRQVRTSVHFRRPVTLKTARQARFPRKSAPK
Sp          ------------------------------------------------MSVAKAKGAQKTVQKG-IHNKVAKKVRTSTTFRRPKTLQLSRKPKYARKSVAH
Dd          -------------------MAGKKVKSNTPKQDLSVSKSKLTSIKAPAAAIKAKAAASAVKKG-VSNKSTRKVRTSVIFRRPVTLNNPKKPAYPRRSVNK
At          -------------------MSPAKVDVT---------------KKADPKAKALKAAKAVKSGQIVKKPAKKIRTKVTFHRPKTLTVPRKPKYPKISATP
Pf          MTQVEKKKNVKNVNKHKIIHKRVIKKGNRKYTNGNTKVKYRKQVVKKTINKNVSKKKLLKENVDKDEKKKMKISIRFKKPKTLIYARNPKCPRIVKSC
Hs_MRPL23   -----------------MARNVVYPLYRLG--------------GPQLRVFRTNFFIQLVR-PGVAQ--PEDTVQFRIPMEMTRVDLRNYLEGIYNV
Ol          -----------------MLESCVVPIYQLG--------------NPQLRIFRPNWFLTLVR-PGKEQ---PPDTAQFRVPMEMTKYDVKNYLEKIYNV
Ci          ----------MSRIRIGTKLPGYKFPLHYNG--------------APQRRIFIPEWYITLVK----PRKEMPKNYVRFHVPADMTKYDMKEYLDKVYNI
Dm          -----------------MSTRWPIYQRG--------------NPQLRVFLPNFWMKLIR-PTEEQ---PPNVTFSVSMEMTKYDVRNYLEKIYKL
Ce          -----------------MTSRLARLWQPG--------------NPQRRVFLPDFWMAVVESPSVGRNRLPRNCVKFEVDPRMSRHDIREYLTKIYDL
Rp_RPL23    ----------------------------------------------------------------------------MSVYKYYDLIRKPIITEKTTSI
Ec          ----------------------------------------------------------------------------MIREERLLKVLRAPHVSEKASTA
```

```
Hs_RPL23A   ---RNK-LDHYAIIKFPLTTESAMKK---IEDNNTLVFIVDVKANKHQIKQAVKKLYDIDVAKVNTLIRP----DGEKKAYVRLAPDYDALDVANKIGII
Ol          ---RNK-LDHYAIIKFPLTTESAMKK---IEDNNTLVFIVDVKANKHQIKHAVKKLYDIDVAKVNTLIRP----DGEKKAYVRLAPDYDALDVANKIGII
Ci          ---RNK-MDQFAVVKHPLTTESAMKK---IEDNNTLVFIVDLKANKPQIKQAVKKLYDIDVAKVNTLIRP----DGRKKAYVKLAPDYDALDVANKIGII
Dm          ---RNR-MDAYNIIKYPLTTEAAMKK---IEDNNTLVFLTHLRANKNHVRAAVRKLYDIKVAKVNVLIRP----DGQKKAYVRLARDYDALDIANKIGII
Ce          ---TSK-MDHFRIIQHPLTTESAMKK---IEEHNTLVFIVSNDANKYQIKDAVHKLYNVQALKVNTLITP----LQQKKAYVRLTADYDALDVANKIGVI
Sp          ---APR-LDEYKIIVNPINSESAMKK---IEDDNTLVFHVHLKANKFTIKEAVRKLYSVEPVKINTLIRP----NGTKKAFVKLSADADALDVANRIGFL
Dd          ---ITK-MDQFRILKAPLTTESATQK---IEGSNTITFMVDMFANKSQVADAVAKMYDVKVKRVNTLITP----RGEKKAFVTLSPEFEAADVANKIGLI
At          ---RNK-LDHYQILKYPLTTESAMKK---IEDNNTLVFIVDIRADKKKIKDAVKKMYDIQTKKVNTLIRP----DGTKKAYVRLTPDYDALDVANKIGII
Pf          ---HSKTLDKYGLIKYPLTSEKAMKK---IEEINTLVFMCDKRANKKNIKKSVKNLFGIECDKVNVLNTL----NGDKKAYVRLSGEHDALEVANKIGIL
Hs_MRPL23   PVAAVRTRVQHGSNKRRDHRNVRIKK-----PDYKVAYVQLAHGQTFTFPDLFPEKDESPEGSAADDLYS----MLEERQQRQSSDPRRGGVPSWFGL-
Ol          PVGVVRTRIQFGSNKKRNHLNQKVKK-----PDYKVAYVQLAQGQTFTFPDVFPEKDQTPAEGSMEEMQN----KFMEDEKQRQKLDPRRGGVTEWFGL-
Ci          PVTSINLSIVNYIRYRQPTPWRKDQYMWKFMEPWKIAHVYLGNNETFEFPEILTKVKEEDGDDEEMEDMDSKTMVEQQEAYSKFAEKQQTSGVSIKDKTY
Dm          PVVDVRTRIAMGETKKDQTYGYITKK-----DDVKLAYVTLPREESFVFPDFFSEKAEKQAK--EEKSLD----ESKAGFRRFLDRNKKRPGTPGWFSI-
Ce          PVRDVRTEVQMGDITWNSKLDHQYKKAMWKDEDKKIAYVFMSKGFEFSYPQMFEALEEDLELVKAMKQQE----ELKDKLNERYANRNRR--VGQFLGA-
Rp_RPL23    ---SEQ--NKYTFYVNKFAKKLSLKR---AIEA---IFKVKVKKVNILNIKGKKRFK------GIIGTQ----INRKKAIVTLEKDHN-IDYAGGIK--
Ec          ---MEK-SNTIVLKVAKDATKAEIKA---AVQK---LFEVEVEVVNTLVVKGKVKRHG------QRIGRR----SDWKKAYVTLKEGQN-LDFVGGAE--
```

Figure S1. Yoshihama *et al.*

```
Hs_RPL23A          ------
Ol                 ------
Ci                 ------
Dm                 ------
Ce                 ------
Sp                 ------
Dd                 ------
At                 ------
Pf                 ------
Hs_MRPL23          ------
Ol                 ------
Ci                 FENKWL
Dm                 ------
Ce                 ------
Rp_RPL23           ------
Ec                 ------


Hs_RPL26           MKFNPFVTSDRS--------------------------------------------KNRKRHFNAPS----------------HIRRKIMSSPLSKELRQ
Ol                 MKLNPFVTSSRR--------------------------------------------KNRKRHFNAPS----------------HIRRKIMSSPLSKELRQ
Ci                 MKFNKLKSSSRR--------------------------------------------KNRKEYFNAPS----------------HIRRKFMGAPLSKELRQ
Dm                 MKQNPFVSSSRR--------------------------------------------KNRKRHFQAPS----------------HIRRRLMSAPLSKELRQ
Ce                 MKVNPFVSSDSG--------------------------------------------KSRKAHFNAPS----------------HERRRIMSAPLTKELRT
Sp                 MKFSRDVTSSRR--------------------------------------------KQRKAHFGAPS----------------SVRRVLMSAPLSKELRE
Dd                 MKRNTNVSSSRR--------------------------------------------KSRKAHFTASS----------------GERRIRMSAALSHDLRT
At                 MKFNPRVSSSRR--------------------------------------------KNRKAHFTAPS----------------SVRRVLMSSPLSKDLRN
Pf                 MKFNKQKSSSRR--------------------------------------------KMRKAHFTAPA----------------GLRRKIMSSKLSKELRL
Hs_MRPL24          MRLSALLALASK----------------------------------------VTLPPHYRYGMSPPG-------------SVADKRKNPPWIRRRPVVVE
Ol                 MRLTALLSMAAK----------------------------------------VVAPKDYRYGTNRPW-------------TVAAKRLNPPGKRRRKVFVE
Ci                 MRLSARLFILRGRSRLPSDPNESERLVKYGERKWRDFQSP-----------VVDPPYWYKWSKDSPYHPVE--------QQKILKGEYLQWYRYNFHKQK
Dm                 MRLTQYLASKLKNFSNLPKEYIERSKKQVYWQTPKEINYL----------PRTVERKRFRYTTNRSW-------------TGQFRQQNMPGTVRRKVLVE
Ce                 MLTSRALRIPRKPFVDLDYARHMPSAYVERTKRTVPRKVFGDRFGAPDIKMYYVHPDDYVPSHRRPWEDKQISSHLHRSDKYFSAQLSNQYFNLRRPKSE
Rp_RPL24           ----------------------------------------------------------------------------------------------------
Ec                 ----------------------------------------------------------------------------------------------------


Hs_RPL26           KYNVRSMPIRKDDEVQVVRGHYKGQQIGKVVQVYRKKYVIYIERVQ-REKANGTTVHVGIHPSKVVITRLKLD--------KDRKKILERKAKSRQV--G
Ol                 KYNVRSMPIRKDDEVQVVRGHYKGQQIGKVVQVYRKKYVIYIERVQ-REKANGTTVHVGIHPSKVVITRLKLD--------KDRKKILERKAKSRAD--G
Ci                 KYSVRSMPIRKDDEVQIVRGHYKGQQVGKIIQVYRKKYVVHIERIQ-REKANGATVHVGIHPSKVVIVKLKLD--------KDRKRILEHKARSKLV--D
Dm                 KYNVRSMPIRRDDEVQVIRGHFKGNQVGKVVQAYRKKFVVYVEKIQ-RENANGTNVYVGIHPSKVLIVKLKLD--------KDRKAILERRGKGRLAALG
Ce                 KHGIRAIPIRTDDEVVVMRGRHKGN-TGRVLRCYRKKFVIHIDKIT-REKANGSTVHIGIHPSKVAITKLKLD--------KDRRALVERKAAGRSRVTG
Sp                 QYKIRSLPVRRDDQITVIRGSNKGR-EGKITSVYRKKFLLLIERVT-REKANGASAPVGIDASKVVITKLHLD--------KDRKDLIVRKG-GKVE---
Dd                 KYNVRSLPIRKDDEVRVVCGDNKNH-EGKVVACYRKKYVIHIDKLT-VTKTNGNTAQIGVSPSNVILTKLKLD--------KDRKALLERKNRTVAADKG
At                 KHNVRSMPIRKDDEVQVVRGTFKGR-EGKVMQVYRRKWVIHIERIT-REKVNGSTVNVGVNASNVMITKLRLD--------KDRKSLLERKANGRAAADK
Pf                 KYKTRSLPVRKDDEVLICRGHNHGR-EGKVVKINRKRYKIYVERVT-REKVNGESTFIGIHPSNVVLTKLKVD--------KNRKKILDRK----AAKEN
Hs_MRPL24          PISDEDWYLFCGDTVEILEGKDAGK-QGKVVQVIRQRNWVVVGGLNTHYRYIGKTMD---YRGTMIPSEAPL--LHRQVKLVDPMDRKPTEIEWRFTEAG
Ol                 PLEAEDWSVVRGDLVEILAGKDKGK-HGKVIQVFRRRNWVILEGLNTHFRYVGKSPD---YRGTYVASEAPL--LLGDVALVDPSDRKPTQVEWKYTEEG
Ci                 VIPPDAWLFRVGDKVEILTGKDKGK-QGDVIQVVQQGNIVVVGGLN-CERIKGRDGMLVRQEKPLEHHEVTLL---------DPKDEKPVDVEFRVNENG
Dm                 PI--EDWSFFRGDRIEVLVGKDKGK-QGIVTQVIPERNWVIVEGLNWHYRKVGGEKE---FPGIIIKSEAPLH-VTKDIRLVDPSDLQGTDFEWRFTEEG
Ce                 RMADTEWTFFPGDLVQVMVGKDKGR-QGLVLTTSRDSSDVIVDGLHTKLGEDMEGSEKLGVDKTLRWQEQPLSVSKKQVMLVDPNDEQPCEARWQLNGPG
Rp_RPL24           ---MIKLKVKKGDEVVIITGKYKGK-KGKVLKVFPEENTVVVSGVN-LVKKHTKPN-KMSE-GGIITQESPIH--ISNIAHIDPKTGNPTKVAFKFLEDG
Ec                 ----MAAKIRRDDEVIVLTGKDKGK-RGKVKNVLSS-GKVIVEGIN-LVKKHQKPVPALNQPGGIVEKEAAIQ--VSNVAIFNAATGKADRVGFRF-EDG


Hs_RPL26           KEKGKYKEET-IEKMQE--------------------------------------------------------------
Ol                 KEKGKYKEET-IEKMQE--------------------------------------------------------------
Ci                 LEKGKHTEET-IQAMES--------------------------------------------------------------
Dm                 KDKGKYTEETAAQPMETA-------------------------------------------------------------
Ce                 ILKGKHTDET-VN------------------------------------------------------------------
Sp                 -------------------------------------------------------------------------------
Dd                 KVKVSADVN----------------------------------------------------------------------
At                 EKGTKFSAEDVMENVD---------------------------------------------------------------
Pf                 -------------------------------------------------------------------------------
Hs_MRPL24          -ERVRVSTRSGRIIPKPEFP--RADGIVPETWIDGP-KDTSVEDALERTYVPCLKTLQEEVMEAMGIKETRKYKKVYWY
Ol                 -ERVRVSVRTGRIIPKPVVE--RRDGIVPQQWKDGP-KDTSPEDALEKTYVPSLKTLEEDVMEKLGIQENRRHRTSYWY
Ci                 -QRVRVSTRSGYIIHWPPEILWDGTP---KTEYECQDKDTTYENACEKTYKPTLDNWQDELKKIYKISDSPR-RETFWY
Dm                 -EKVRVSLRSGRIIPIPETNNQTHDYKTPNAYIERE-KDTPGAVVGEITFQPKLSTFEMDIMEEMGIKEERTPVKSYWY
Ce                 DEYIRVSTRSGYEIPIPSQAKVTYEYLQPENYIEVEGKDTPADAVLERTYMPKVASFEQEIMEEMGIKEERTPKPTFWY
Rp_RPL24           -SKVRVAKKSGEIIGKVGNNVKV--------------------------------------------------------
Ec                 -KKVRFFKSNSETIK----------------------------------------------------------------


Hs_RPL27A          -----------------------MP-SRLRKTRKLRGHVSHGHGRIG----KHRKHPGGRGNAGGLHHHRINFDKYHPGYFGKVG-----MKHYHLKRNQ
Ol                 -----------------------MP-TKKSKTRKLRGHVSHGHGRIG----KHRKHPGGRGNAGGLHHHRINFDKYHPGYFGKVG-----MRHYHLKRNT
Ci                 -----------------------MV-ARNKKTRKLRGHVSHGHGRVG----KHRKHPGGRGNAGGQHHHRINYDKYHPGYFGKVG-----MRHFHLKRNP
Dm                 -----------------------MSNIKRKKTRKLRGHVSHGHGRIG----KHRKHPGGRGNAGGMHHHRINFDKYHPGYFGKVG-----MRNFHLRRQH
Ce                 -----------------------MA-HALRKTRKLRGHVSHGHGRIG----KHRKHPGGRGNAGGQHHHRINRDKYHPGYFGKVG-----MRVFHLNKNQ
Sp                 -----------------------MP-THTSKTRKLRGHVSAGHGRIG----KHRKHPGGRGKAGGLQHLRSHFDKYHPGYFGKVG-----MRRFHLMKNP
Dd                 -----------------------MP-TRFSKHRKSRGDVCAGYGRVG----KHRKHPGGRGNAGGLTHHRINFDKYHPGYFGKLG-----MRHFHLLRNQ
At                 -----------------------MA-TALKKNRKKRGHVSAGHGRIG----KHRKHPGGRGNAGGMHHHRILFDKYHPGYFGKVG-----MRYFHKLRNK
Pf                 -----------------------MA-TRFKKNRKKRGHVSAGHGRVG----KHRKHPGGRGKAGGLHHMRINFDKYHPGYFGKVG-----MRHLNLLKNR
Hs_MRPL15          MAGPLQGGGARALDLLRGLPRVSLANLKPNPGSKKPERRP-RGRRRG--RKCGRGHKGERQR-GTRPRLGFEGGQ--TPFYIRIP-KYGFNEGHSFRRQY
Ol                 MSFPKKPGGK-ALDVLQNLPRITLANLRPEPGARKRERRRGRGQHGG--NRSGRGHKGERQR-GTRPRLGFEGGQ--TPFYLAIP-KYGFNEGHSRRPQY
Ci                 MSN---SSYK---SLLRNLPRVNLQNLKANPYAEVKHSPN-RGPH-------AIGLKNGVRP-NIKPRIGYVQDK--TPFQKRIP-KYGYNREQHLKRQY
Dm                 MAHLRETSDK-ALKLLRTLPRVQIGNLRPNPNSKQNDKRG-RAQHGG--DKHGAGNKGSGQR-QNFMRLGYETGN--QPFYLRFP-YEPYYKGHHLKRQY
Ce                 MSQAARSAKERALQIVEQASRIRIQDARDNIGARTTGRQLKKGHN------QMGHTQGALER-AAKPPLGWIWGDFFRPWQRMYPGEKLFNADINTRREY
Rp_RPL15           ---------------------MKLNELYNNLGAKKNKKRIARGIGSGKGKTAGRGIKGQKSRSGVAIK-GFEGGQ--TPMIKRLP-KRGFK--CISTKKY
Ec                 ---------------------MRLNTLSPAEGSKKAGKRLGRGIGSGLGKTGGRGHKGQKSRSGGGVRRGFEGGQ--MPLYRRLP-KFGF-----TSRKA


Hs_RPL27A          SFCPTVNLDKLWTLVSEQTRVNAAKNKTGAAPIIDVVRSGYYK----VLGKGKLPK-QPVIVKAKFFSRRAEEKIKSVGGACVLVA--------------
Ol                 TYCPTINLDKLWTLVSEQTRVNYGKKPDGPAPVIDAVRAGYYK----VLGKGKLPK-QPVIVKAKFFSRQAEEKIKAVGGACVLMA--------------
Ci                 QACPIINLDRLWTLVSEQTREAY-KDRTDVAPVIDCSKAGFYK----VLGKGILPK-QPVIVKARFFSRRAEEKIKAVGGTCVLVA--------------
Dm                 KFRPEINLDKLWSLVGAEKFAELEKEKSTKAPVIDLVKFGYYK----LLGRGHLPA-RPVIVKAKYFSKKAEDKIKKAGGVCLLSA--------------
Ce                 HYCPTVNVERLWSLVPQEVRDKATGGKS---PVIDCTKLGYFK----VLGKGLLPE-TPLIVKARFFSHEAEQKIKKAGGACVLVA--------------
Sp                 LWRPTVNLDRLWTLVPNETREKYLGKNTEVAPVINVLQSGYGK----VLGKGRLPD-TPVIIQTRYVSRRAEEKIKQAGGVVELIA--------------
Dd                 YHCPTVSLEKIWTLVPESVRKSLAAKNDGTAPVVDVTQKGFFK----VLGHGILPT-QPIIVKARYFSKVAEKKIKAVGGACILVA--------------
At                 FFCPIVNLDKLWSLVPEDVKAKSS--KD-NVPLIDVTQHGFFK----VLGKGHLPENKPFVVKAKLISKTAEKKIKEAGGAVVLTA--------------
Pf                 TYCPTINVDKLWGLLPEEKKKEFSENKD-IAPVIDVTRKGYFK----VLGNGKLKHNQPIVVKARYFSSVAEKKIKAVGGQCILVA--------------
Hs_MRPL15          KPLSLNRLQYLIDLGRVDPSQPIDLTQLVNGRGVTIQPLKRDY-GVQLVEEGADTFTAKVNIEVQLASELAIAAIEKNGGVVTTAFYDPRSLDIVCKPVP
Ol                 QPLSLKRLQYLIDLGRVDPTQPIDLTQLVNARGVTIQPLKRDY-GVQLVDEGSDIFAAKINIEVQRASEGAIAAVERNGGVITTSFYDPISIEILIRPVP
Ci                 FPLTLWQLQRHVDLGRIDPTKPVDITAISNGRVLSMVGSESNYYGIYLLEQGANIFKAKLDIEVQIVDELALATVEKAGGTVSTAFYDRRSFVALCNPVE
Dm                 PPISLLQLQVLIDTNRIDISQPIDISTLCNSGLLKLKPAEMEY-GFQLTDDGLDNFQAKINIEVQHASEAVIAAIEKNGGVIRTAYYDPRSLHMLANPKK
Ce                 IPLSLVELARLIDLGWINPRQPIDISTLCTTQKFQINPKMRQY-GFDLTEEGADSFPYSIDIEVQYATPSAIAAVEKAGGRVRTAYYDVESLEAAINPKC
Rp_RPL15           NIINIYNIEEALTDGRLSTNDIITKEKLLEVGLINNKNLVKLL------SICSDDFASPLSLKLDAYSSKAKYLIEKVGGQLL-----------------
Ec                 AIT----AEIRLSDLAKVEGGVVDLNTLKAANIIG-IQIEFAK------VILAGEVTTPVTVRGLRVTKGARAAIEAAGGKIEE----------------
```

Figure S1. Yoshihama *et al.*

```
Hs_RPL27A     ----------------------------------------------------------------------------------------------------
Ol            ----------------------------------------------------------------------------------------------------
Ci            ----------------------------------------------------------------------------------------------------
Dm            ----------------------------------------------------------------------------------------------------
Ce            ----------------------------------------------------------------------------------------------------
Sp            ----------------------------------------------------------------------------------------------------
Dd            ----------------------------------------------------------------------------------------------------
At            ----------------------------------------------------------------------------------------------------
Pf            ----------------------------------------------------------------------------------------------------
Hs_MRPL15     FFLRGQPIPKRMLPPEELVPYYTDAKNRGYLADPAKFPEARLELARKYGYILPDITKDELFKMLCTRKDPRQIFFGLAPGWVVNMADKKILKPTDENLLK
Ol            FFMRGQPIPKRMLPGEDMVPYYTSAENRGYLADPDKIQQARLALAQKYGYVLPDISRDELYHMLAMRKDVRQVFFGLSPGWVVNMAERKILKPTDEKLLK
Ci            YFLMGKPIHKRLLPPQELVTYYTDPKVRGYLSDPAKIWEERMKLAKKYGYDLPELSEEQKHLMVEGKKDPRQVFNGLMPGSIVNLADKTVLVPENEHFKQ
Dm            WFEKGVPIPSRMLPPQDAVDYYTDPKNRGYLANPEAISQDRLVLAQKYGYQLPKIEEDSAYEMLTTAKDPRQVFYGLEPGWLINIVDKTIIKRKQ-----
Ce            WFEAGKVVPKRKQPPQSLLEYYMDAKNRGYLCEEQEIEKERALSAQIRGYTLPPPPSS------AALKAVDQVFHGIPSGSVVSLADRKVFVTKNELHRE
Rp_RPL15      ----------------------------------------------------------------------------------------------------
Ec            ----------------------------------------------------------------------------------------------------

Hs_RPL27A     ------------
Ol            ------------
Ci            ------------
Dm            ------------
Ce            ------------
Sp            ------------
Dd            ------------
At            ------------
Pf            ------------
Hs_MRPL15     YYTS--------
Ol            YYTS--------
Ci            HAAM--------
Dm            ------------
Ce            YYSSRRADKLYS
Rp_RPL15      ------------
Ec            ------------


Hs_RPP0       MPREDRATWKSNYFLKIIQLLDDYPKCFIVGADNVGSKQMQQIRMSLRGKAVVLMGKNTMMRKAIRGHLEN--NPALEKLLPHIRGNVGFVFTKEDLTEI
Ci            MPREDRKTWKANYFVKLINLLDEYPKCFIVGADNVSSKQMQQIRTSLREKAVVLMGKNTMMRKAIKGHLNN--NPDLERLLPHIRGNVGLVFTKEDLIET
Dm            MVRENKAAWKAQYFIKVVELFDEFPKCFIVGADNVGSKQMQNIRTSLRGLAVVLMGKNTMMRKAIRGHLEN--NPQLEKLLPHIKGNVGFVFTKGDLAEV
Ce            MVREDRSTWKANYFTKLVELFEEYPKCLLVGVDNVGSKQMQEIRQAMRGHAEILMGKNTMIRKALRGHLGK--NPSLEKLLPHIVENVGFVFTKEDLGEI
Sp            MAISKES--KAQYFEKLRSLFEKYNSLFVVNIDNVSSQQMHTVRKQLRGTAELIMGKNTMIRRAMRGIIND--MPELERLLPVVRGNVGFVFTNADLKEV
Dd            -MSGAGSKRKNVFIEKATKLFTTYDKMIVAEADFVGSSQLQKIRKSIRGIGAVLMGKKTMIRKVIRDLADS--KPELDALNTYLKQNTCIIFCKDNIAEV
At            MVKATKAEKKIAYDTKLCQLIDEYTQILVVAADNVGSTQLQNIRKGLRGDSVVLMGKNTMMKRSVRIHSENTGNTAILNLLPLLQGNVGLIFTKGDLKEV
Pf            MAKLSKQQKKQMYIEKLSSLIQQYSKILIVHVDNVGSNQMASVRKSLRGKATILMGKNTRIRTALKKNLQA--VPQIEKLLPLVKLNMGFVFCKDDLSEI
Hs_MRPL10     ------------------------------MAAAVAG--------MLRGGLLPQAGRLPTLQTVRYGSKAV------------TRHRRVMHFQRQKLMAV
Ol            ------------------------------MAATLCAKLLPKQASVLQFLSLLLSGWLPLSVSVRHGSKAV------------TRHRRPQHILKKKLLAV
Dm            ------------------------------------------MATLIQRSLSLAKSSTPALQFLRFRGKIN------------IQRPKAPHYERARVVAV
Ce            ---------------------------------------------MLNQSLRPLVNVQVRSVSSKYPRPTP-------------RHFKRRLFEAALKPEL
Rp_RPL10      ----------------------------------------------------------------------------------------------------
Ec            ----------------------------------------------------------------------------------------------------


Hs_RPP0       RDMLLANKVPAA-ARAGAIAPC-EVTVPAQNTGLGPEKTSFFQALGITTKISRGTIEILSDVQLIKTGDKVGASEATLLNMLNISPFSFGLVIQQVFDNG
Ci            RDLLLANKVAAP-ARAGAVAPN-DVIVPAQNTGLGPEKTSFFQALAIPTKISRGTIEIISDVHLIKPGDKVGASDATLLSMLKIFPFTYGLEIEQVYDSG
Dm            RDKLLESKVRAP-ARPGAIAPL-HVIIPAQNTGLGPEKTSFFQALSIPTKISKGTIEIINDVPILKPGDKVGASEATLLNMLNISPFSYGLIVNQVYDSG
Ce            RSKLLENRKGAP-AKAGAIAPC-DVKLPPQNTGMGPEKTSFFQALQIPTKIARGTIEILNDVHLIKEGDKVGASESALLNMLGVTPFSYGLVVRQVYDDG
Sp            RETIIANVIAAP-ARPNAIAPL-DVFVPAGNTGMEPGKTSFFQALGIPTKITRGTIEITSDVHLVSKDAKVGPSEATLLNMLNISPFTYGMDVLTIYDQG
Dd            KRVINTQRVGAP-AKAGVFAPN-DVIIPAGPTGMEPTQTSFLQDLKIATKINRGQIDIVNEVHIIKTGQKVGASEATLLQKLNIKPFTYGLEPKIIYDAG
At            SEEVAKYKVGAP-ARVGLVAPI-DVVVQPGNTGLDPSQTSFFQVLNIPTKINKGTVEIITPVELIKQGDKVGSSEAALLAKLGIRPFSYGLVVQSVYDNG
Pf            RNIILDNKSPAP-ARLGVIAPI-DVFIPPGPTGMDPSHTSFFQSLGISTKIVKGQIEIQEHVHLIKQGEKVTASSATLLQKFNMKPFSYGVDVRTVYDDG
Hs_MRPL10     TEYIPPKPAIHPSCLPSPPSPP-QEE-IGLIRLLRREIAAVFQDNRMIAVCQNVALSAEDKLLMRHQLRKHKILMKVFPNQV-LKPFLEDSKYQNLLP--
Ol            TEYIPPTRDAPPGAYPHQIKRG-QEECTGLMLLIKEDLKKVFQDYKMIAVVQNNNSNTDDMLTLRHRLHKHGISVKFFPNQV-MHLFLGDSIYGSMAP--
Dm            TQPKYPELPKAKSCFKTRAERTQQQQENPYNEIIAREVRNWLDHSRLVAFFHLSSITADDIFRVRVQLHKQNLHLKSYGSKIIEQAVKN-TRYEAIVP--
Ce            PPTVNMCIPPGLIKREHTGKSKEYSDVELALSNLVRD-WMVREEFRVMAVCQFLPVPGRTLWFAKNQLRSKNIEFRSYGNKI-LKKIFDKTPMSSLNT--
Rp_RPL10      -------------------------MLRSEKPVAVEDIVNIYKESPSVIITHYHGLTVSQVSSLREELKSKEAGFKVVKNTL-AKIAAKQTGLDSITN--
Ec            -----------------------MALNLQDKQAIVAEVSEVAKGALSAVVADSRGVTVDKMTELRKAGREAGVYMRVVRNTL-LRRAVEGTPFECLKD--


Hs_RPP0       SIYNPEVLDITEET--LHSRFLEGVRN-VASVCLQIGYPTVASVPHSIINGYKRVLALSVETDYTFPLAEKVKAFLADPSAFVAAAPVAAATTAAPAAAA
Ci            SVFSPEVLDITDDD--IRRKFTNAVRN-VASVCLAIGYPTVASAPHSIINGFKNILAVAAETEITFPEAEKMKAYLADPTAFAVAAAPAASDEKKEEAKP
Dm            SIFSPEILDIKPED--LRAKFQQGVAN-LAAVCLSVGYPTIASAPHSIANGFKNLLAIAATTEVEFKEATTIKEYIKDPSKFAAAASASAAPAAGGATEK
Ce            TLYTPEVLDMTTEE--LRKRFLSGVRN-VASVSLAVNYPTLASVAHSLANGLQNMLGVAAVTDVSFKEAETIKAFIADPSKFAAAAPAAAAAPAAAAPAA
Sp            NVFSPEILDVSEED--LIGHLLSAASI-ITAISLGANYPTILSVMHSVVNAYKNLVAVSLATEYTFEGTEQTKAFLADPSAFVVAAAPAAAAGGEAEAPA
Dd            ACYSP---SISEED--LINKFKQGIFN-IAAISLEIGYPTVASIPHSVMNAFKNLLAISFETSYTFDAAEKFKS----------AAAAAPVAAAPSAAAPAA
At            SVFSPEVLDLTEDQ--LVEKFASGISM-VTSLALAVSYPTLAAAPHMFINAYKNALAIAVATEYTFPQAEKVKEYLKDPSKFAVASVAAVSADAGGGAPA
Pf            VIYDAKVLDITDED--ILEKFSKGVSN-VAALSRATGVITEASYPHVFVEAFKNIVALIIDSDYTFPLMENIKKMVENPEAFAAVAAPASAAKAD-EPKK
Hs_MRPL10     LFVGHNMLLVSEEP--KVKEMVRILRT-VPFLPLLGGCIDDTILSRQGFINYSKLPSLPLVQGELVGGLTCLTAQTHSLLQHQPLQLTTLLDQYIREQRE
Ol            LFIGPTVLFVSKTP--KLREMLSTLRV-SPQMTLLGACIDNTLLSVQGVLHYSKLPSMTVVQGELVSGLTMLTSHTVSVLQRHPGQLSALLQQYIKQQ--
Dm            LFHSNHCIVFSPDPE-KTAALLRIVRR-VPQMVLLGGIVEETMLSRNQLVAYAQMPGLHAVQAQLVQTLSQAPGHLIQQLQAHQNSFVQVLDVHAKNQMN
Ce            VLVGNNAILLAKDIS-AIKSILQETDK-LNWIEPLVMMADGRIVDMVDARELAKLSSLEDLRAHTVQLLGQQVAQVTISLDTVTRHLPSLLDSHISTNQE
Rp_RPL10      LFAGPTAIVYSKEPV-EMARLVVNFANSNDNLKIIGGIVDNHILDAYSIKELSKLPSLHELRGKIVGLLQAPATKVVGVLQATSSSIARVIHAHAIKH--
Ec            AFVGPTLIAYSMEHPGAAARLFKEFAKANAKFEVKAAAFEGELIPASQIDRLATLPTYEEAIARLMATMKEASAGKLVRTLAAVRDAKEAA---------


Hs_RPP0       APAKVEAKEESEESDEDMG--FGLFD--
Ci            A------EESEEESDDDMG--FGLFD--
Dm            KEEAKKPESESEEEDDDMG--FGLFD--
Ce            KK-----EEPKEESDDDMG--FGLFD--
Sp            AEA---AAEEEEESDEDMG--FGLFD--
Dd            AAKKVVVEEKKEESDDDMG--MGLFD--
At            AAK---VEEKEESDEEDYGGDFGLFDEE
Pf            EEAKKVEEEEEEEEDGFMG--FGMFD--
Hs_MRPL10     KDSVMSANGKPDPDTVPDS---------
Ol            -SSETTSEAKTESAT-------------
Dm            -------EETPKST--------------
Ce            AKK-------------------------
Rp_RPL10      ----------------------------
Ec            ----------------------------
```

Figure S1. Yoshihama *et al.*

```
Hs_RPP1     -----------------------------------------------------------------MASVS----------ELACIYSALILHDDEVTVT-
Ol          -----------------------------------------------------------------MASVS----------ELACIYSALILHDDEVTVT-
Ci          ------------------------------------------------------------------MSTS----------ELACVYASLILSDDQVDIT-
Dm          -----------------------------------------------------------------MSTKA----------ELACVYASLILVDDDVAVT-
Ce          -----------------------------------------------------------------MASNQ----------ELACVYAALILQDDEVAIT-
Sp          ------------------------------------------------------------------MSAS----------ELATSYSALILADEGIEIT-
Dd          -----------------------------------------------------------------MSEIKT--------EELACIYSGLLQDDGIEIT-
At          -----------------------------------------------------------------MSTVG----------ELACSYAVMILEDEGIAIT-
Pf          -----------------------------------------------------------------MASIPASELPECEKQELLCTYAALILHEEKMSIT-
Hs_MRPL12   MLPAAARPLWGPCLGLRAAAFRLARR---QVPCVCAVRHMRSSGHQRCEALAGAP---LDNAPKEYPPKIQQLVQDIASLTLLEISDLNELLKKTLKIQD
Ol          -MYCTSRCLR-TTLRAAASTHRYRQQLQHQVPSLCALRLLKTSCHS--ETIATPP---LDGAPKQYSPKVQQLVNDIASLTLLEVSDLNELLKKTLNIQD
Ci          --PILWRIIRPTVKCLSAVN---------KSSALHTRVLLSRTATTNAAVEEPLPPPERDNEARVFEPKIVNLVDSISSLTLREVADLNELLKSTLNIKD
Dm          -MHITRLALRQ----ISRQV---------QLHRMYSAAAPAAAVSGAEKLVPPAP----EGAAKPPNPKLDSIVNNIAALNLLEVAELSTLLKQKLNLPE
Ce          ---MLSRTLRP----ISKVL---------RSSAKYS---QAAGLPLNEGSPAPLP-----SEDRAISAKVSSLVEEIANLSLLDVSDLNWALKKRLNIPD
Rp_RPL12    ------------------------------------------------------------------MADLAKIEEQLSSLTLMQAAELVKMLEEKWGVS-
Ec          -----------------------------------------------------------------MSITKDQIIEAVAAMSVMDVVELISAMEEKFGVS-

Hs_RPP1     -------------EDKINALIKAAGVNVEPFWPGLFAKALANVNIGSLICNVGAGGPAPAAGAAPAGGPAPSTAAAPA-EEKKVEAKKEESEESDDDMGF
Ol          -------------ADKLNALIKAAGVTVEPFWPTLFAKALSSIDIGSLICNVGAGGAAPAG--APAAGAAAAAADAPAKEEEKKEEKKEESEESDDDMGF
Ci          -------------ADRISKLLSAANVTVEPFWPGLFAKALDGVDIKSLICNVGSGSGAPAG-----GAAAAEETKAEVKEEEKKE---ESEEESDDDMGF
Dm          -------------GEKINTILKAANVEVEPYWPGLFAKALEGINVKDLITNIGSGVGAAPAGGAAPAAAAAAPAAESKKEEKKKE---EESDQSDDDMGF
Ce          -------------GEKIATLLKAANVEFEPYWPGLFAKALEGVDVKNLITSVSSGAGSGPAP-AAAAAAPAAGGAAPAAETKKKE---EPKEESDDDMGF
Sp          -------------SDKLLSLTKAANVDVEPIWATIFAKALEGKDLKELLLNIGSAAAAPAAG--GAGAPAAAAGGEAAAEEQKEE--AKEEEESDEDMGF
Dd          -------------ADKIKTLLEAANITVASHWPGLYARSLAKVNIPELLLNAGSSGAAGAAP-VAAATSAAAPAAAAKKETKKEE---VKKEESDDDMGM
At          -------------ADKIATLVKAAGVSIESYWPMLFAKMAEKRNVTDLIMNVGAGGGGGAPVAAAAPAAGGGAAAAPAAEEKKKD---EPAEESDGDLGF
Pf          -------------SDNILKLIKNSNNTVLPYLPMLFERALKGKDIQSLLSNLSVGSAPAAA-----AQVTTEKPSEDKKEAKKEE--KVEEEEEEDDLGF
Hs_MRPL12   VGLVPMGGVMSGAVPAAAAQEAVEEDIPIAKERTHFTVRLTEAKPVDKVKLIKEIKNYIQGINLVQAKKLVESLPQEIKANVAKAEAEKIKAALEAVGGT
Ol          VGMMPMGAMAASAMPATPAAD--EEEAPAKKEKTHFTVKLTELKAADKVKLIKEVKNCIQGLNLVQAKKLVESLPQEIRANVSKEEAEKLKAALEAAGGT
Ci          TPMMAA----QVAAPAAAAEAE-EKQAVPEK--TEFGVKLLEFSPADKIKLIKAIKAVKENFNLVQAKKFVESLPQIVKDEASKEEAEEMKKILEEAGGK
Dm          TAFAPQ----FAAGPARAAPAEDEEEAAPKKVQTSFKVKLVKFDEKQKVALIKEVKNLLEGMNLVQAKKFVESAPTIVKEDIPKEEAEKLKEALSKAGAI
Ce          QPLMAA----AAAAPAAQAEAE-AASDVPQK--MTFTVKLTKFDDTKKIAIIKEIRNAIPGLNLVQAKKFVETAPVNVKEDLGKAEADELKAILEKAGAT
Rp_RPL12    ----------AAAPITVASAGVAAPLAEAVTEKTEFEVVLTATGD-KKVEVIKIVKDITG-LGLIEAKKLVDEAPKSIKNNVKKAEAEEIKSKLEAAGAK
Ec          ----------AAAAVAVAAG-----PVEAAEEKTEFDVILKAAGA-NKVAVIKAVRGATG-LGLKEAKDLVESAPAALKEGVSKDDAEALKKALEEAGAE

Hs_RPP1     GLFD
Ol          GLFD
Ci          GLFD
Dm          GLFD
Ce          GLFD
Sp          GLFD
Dd          GLFD
At          GLFD
Pf          SLFG
Hs_MRPL12   VVLE
Ol          VVLE
Ci          VDIV
Dm          IEIE
Ce          IEIV
Rp_RPL12    VELK
Ec          VEVK
```

Figure S1. Yoshihama *et al.*
