# Supplementary material for: Analysis of Ribosomal Protein Gene Structures: Implications for Intron Evolution
Source: PLoS Genet. 2006 Mar 3;2(3):e25. doi: 10.1371/journal.pgen.0020025 (PMC1386722; doi:10.1371/journal.pgen.0020025)
Supplement: Supplementary file 2 [file pgen.0020025.sg002.pdf]

		Exon	Intron	Exon
Hs	*RPS13*	GAACAGAAAG	+	GATAAGGATG
Ol		AAACAGAAAG	+	GACAAAGATG
Ci		AAACAGAAAA	+	GACACTGACT
Dm		CAAGGACAAG		GACGGCAAGT
Ce		ATCCCGCAAG		GACATTGACA
Sp		TAACCGTAAG		GATAAGGACT
Dd		AGCCCGTAAA		GATTACGATG
At		GAACAGGAAA		GATAAGGATT
Pf		GAACAAGAAA		GATAAAGATT
Hs	*MRPS15*	ACATCGAAAG	+	GACAAAGCCC
Ol		ACATCACAAG	+	GACAAAGCTA
Ci		AATATTTAGA		CAGGACAAAA
Dm		GGAGCACATG		GACAAGCATC
Ce		CATTGGACAG		GAAACCAAAA

		Exon	Intron	Exon
Hs	*RPS23-2*	CTTTATTGAG	+	GAAAATGATG
Ol		CTTCATCGAG	+	GAAAACGACG
Ci		CTATATTGAG	+	GAAAACGATG
Dm		CTACATTGAG		GAGAACGACG
Ce		CTTTGTTGAA	+	GAAAACGACG
Sp		CTTTGTCGAT		GAGAACGACG
Dd		TTACATCCAA		GAAAATGACA
At		CTACATTGAA		GAAAATGACG
Pf		TTTCATCGAT		GAAAATGACG
Hs	*MRPS12-2*	CACCCTGCAG		GAGCACCAGA
Ol		CAACCTGCAG		GAGCATAATG
Ci		TAATCTTCAG	+	CAACATAATG
Dm		CAACCTGCAA		GAGCACAACA
Ce		CAATTTGCAG		GAACATTCAC

		Exon	Intron	Exon
Hs	*RPL8-2*	CGGGCATCAG		GGAACTATGC
Ol		CGAGCCTCAG		GGAACTACGC
Ci		AAAACCTCTG		GAAATTATGC
Dm		CGCACCTCTG		GCAACTACGC
Ce		CGTGCCTCTG	+	GTAACTACGC
Sp		CGTTCTTCTG		GTAACTACGT
Dd		CGTTGTTCAG		GTAACTATGC
At		AGAGCTTCTG		GTGATTACGC
Pf		AAAGCTTCCG		GATGTTATGC
Hs	*MRPL2-2*	CGAGCTGCAG	+	GGACGTGTGG
Ol		CGTGCTGCAG	+	GAACCAGCGG
Ci		AGATCTGCAG		GTGCTTACTG
Dm		CATGCCGCCG		GCACCTTCGG
Ce		AAAGCCGCCG		GAACTTCAGC

		Exon	Intron	Exon
Hs	*RPS14*	GATCTTTCTG		GCAAGGAAAC
Ol		GTGGCCGAGG		GCGAGAATGT
Ci		GATCTATCTG		GCAAAGAAAC
Dm		GATCTGTCCG		GCCGTGAGAC
Ce		GATATCTCAG	+	GACGTGAAAC
Sp		GATTTGACCG		GCAAGGAAAC
Dd		TATCCTCAAA		AGAAACCATT
At		GATTTGTCTG		GTCGTGAAAC
Pf		GATTTAAGTG		GAAGAGAAAC
Hs	*MRPS11*	TCTGCTAGTA		ATGAGCCCCT
Ol		GACAGCGAGG		GACAGTCTCT
Ci		AAGTACCGGA		AAGAAGTTTT
Dm		GATCACAAAG	+	GAGTCCTCCG
Ce		GTTATGGATA		ATAAGAACGA

		Exon	Intron	Exon
Hs	*RPL3*	CAACAAGAAG	+	ATTTATAAGA
Ol		CAACAAGAAG		ATCTACAAGA
Ci		GAACAAGAAG		ATCTACAGAT
Dm		CAACAAGAAG		ATCTACCGCA
Ce		CAACAACAAG		ATCTACCGTA
Sp		GAACTCCAAG		ATCTACCGCA
Dd		AAACAAGAAG		ATTTACAGAA
At		TAACAAGAAG		ATTTACAGGT
Pf		AAATAAAAAA		ATTTATAGAA
Hs	*MRPL3*	TGGACTGAAA	+	GTGTGGAGAA
Ol		TGGACTGAAG	+	ATATGGAGGG
Ci		AGGACTAAAG	+	GTTTTAAGGA
Dm		GGGTCTGCGC		GTGTGGCGCA
Ce		TGGTTTGGAA	+	ATTGTTCGAA

		Exon	Intron	Exon
Hs	*RPL10*	CCGCCAGAAG	+	ATCCACATCT
Ci		GCGTCAGAAG	+	ATCTATGATT
Dm		ACGTCAAAAG	+	ATCTATGTTT
Ce		ACGTCAAATC		ATCGTCTCTT
Sp		TCAACAGAGA		ATCATTGTCT
Dd		TCGTCAAAAG		ATCGTTGTCT
At		TCGTCAAAAG		ATCATTGTTA
Pf		AAGACAAAAA		GTTTTTGTTA
Hs	*MRPL16*	CGCAGCAAAG		GCTGTGAGCC
Ol		CCCTGCTAAG	+	GTTATGAGCA
Ci		GAAGGCACTT		CCTGTAGATG
Dm		CCAGGCCACC		GTCGTTTCCC
Ce		CCCAGTCGAG		TTCGTAAGTG

		Exon	Intron	Exon
Hs	*RPS16*	ACAGTACAAG	+	CTGCTGGAGC
Ol		TCAGTACAAG	+	CTTCTGGAGC
Ci		CAGATATAAG	+	ATGGAGGAAC
Dm		GCAATACAAA		CTGCAGGAGC
Ce		GCGCATCAAG		CTTCAGGAGC
Sp		GCGCATGAAA		GTTTACGAAC
Dd		AAAATTAAAA		GTTTACGAAC
At		CCGATTCAAG		ATCTTCGAGC
Pf		AAAAACTAAA		GTATATGAAC
Hs	*MRPS9*	CAGAGAACAG		CTGATGTTCC
Ol		CAGAGAGCAG		CTGATGTTCC
Ci		AAGGGAGATG		GTGATTGCCC
Dm		CAGAGAACAG	+	CTGCTCTTCC
Ce		CAGAGAGATT		CTTCTTGCAC

		Exon	Intron	Exon
Hs	*RPL7*	CCTTCGTCAA		ATCTTCAATG
Ol		TCTGCGCCAG		ATCTTCAACG
Ci		ACTTCGTCAA		ATCCACAATG
Dm		ACTTCGTCAA		ATCCACAATG
Ce		TCTTCGTCAG		ATCAACAACG
Sp		TATCCGTGGT		ATTAACAATA
Dd		ACTCTTACAA		TTAAACAATG
At		TTTGAGACAG	+	ATTTTCAATG
Pf		ATTATTACAA		GTACACAATG
Hs	*MRPL30*	ATTAGAAAAA	+	GCACATACCC
Ol		CCTCCAAAAG	+	GCACATGTAC
Ci		TCTATATCGG	+	GTAAATGATA
Dm		ACTAGACGGA		AAGCAGAGCG
Ce		TGGAGCGGAT		TTTAAGGTCG

		Exon	Intron	Exon
Hs	*RPL23*	CAGAAAAAAG		GTACATCCAG
Ol		CAGGAAAAAA		GTGCATCCAG
Ci		TCGGAAAAAA		GTGCATCCTG
Dm		CAGGAAGAAG	+	GTCATGCCTG
Ce		CAGAAAGAAG		GTTCTCCAAG
Sp		GCGTAAGAAG		ATCATGCCTG
Dd		AAGAAAGAAA		GTTTGCACTG
At		CCGTAAGAAG		GTGCTTCCTG
Pf		AAGAAAGAAA		GTTTTGAATG
Hs	*MRPL14*	GGGCGACCAG		ATACTACTGG
Ol		GGGCGACAAG		GTGCTGCTCG
Ci		TGGAGACCAG		GTTTTGATCA
Dm		TGGCGATAAG		GTGCTGGTGG
Ce		CGGTGACAAG	+	ATTCTCGTTG

Figure S2 Yoshihama et al.
