# Supplementary material for: Analysis of Ribosomal Protein Gene Structures: Implications for Intron Evolution
Source: PLoS Genet. 2006 Mar 3;2(3):e25. doi: 10.1371/journal.pgen.0020025 (PMC1386722; doi:10.1371/journal.pgen.0020025)
Supplement: Supplementary file 3 [file pgen.0020025.st001.pdf]

Table S1. List of CRP and MRP homologs.

Gene	No. of intron	Cytoprasm Accession number*	Gene	No. of intron	Mitochondrion Accession number*
RPSA			MRPS2		
Hs	5	NT_022517	Hs	3	NT_019501
Ol	6	scaffold5963	Ol	3	scaffold2983
Ci	1	scaffold_549	Ci	3	scaffold_169
Dm	1	NC_004354	Dm	1	NT_033779
Ce	3	NC_003281	Ce	3	NC_003283
Sp	1	NC_003423†			
Dd	0	DDB0190962			
At	4	NC_003070†			
Pf	1	NC_004314			
RPS2			MRPS5		
Hs	5	NT_037887	Hs	11	NT_026970
Ol	5	scaffold14704	Ol	11	scaffold9496, 6077‡
Ci	4	scaffold_42	Ci	2	scaffold_128
Dm	0	NT_033779	Dm	1	AABU01002755
Ce	2	NC_003282	Ce	5	NC_003283
Sp	0	NC_003421			
Dd	0	DDB0219896			
At	1	NC_003070†			
Pf	3	NC_004317			
RPS5			MRPS7		
Hs	4	NT_011109	Hs	4	NT_010641
Ol	5	scaffold18501	Ol	4	scaffold1530
Ci	4	scaffold_191	Ci	0	scaffold_43
Dm	3	NC_004354†	Dm	1	NT_033779
Ce	3	NC_003282	Ce	4	NC_003282
Sp	1	NC_003424†			
Dd	1	DDB0186804			
At	3	NC_003071†			
Pf	1	NC_004328			
RPS11			MRPS17		
Hs	4	NT_011109	Hs	1	NT_033968
Ol	4	scaffold7322	Ol	1	scaffold899
Ci	3	scaffold_130	Ci	2	scaffold_219
Dm	3	NT_033778	Dm	1	NT_033778
Ce	2	NC_003282	Ce	2	NC_003281
Sp	0	NC_003424†			
Dd	1	DDB0204861			
At	5	NC_003074†			
Pf	2	NC_000521			
RPS13			MRPS15		
Hs	5	NT_009237	Hs	7	NT_004511
Ol	5	scaffold3300	Ol	7	scaffold5573, 100804, 35546‡
Ci	4	scaffold_41	Ci	5	scaffold_327
Dm	2	NT_033779	Dm	2	NT_033778
Ce	2	NC_003281	Ce	7	NC_003279
Sp	3	NC_003424			
Dd	2	DDB0186065			
At	4	NC_003074†			
Pf	0	NC_004331			
RPS14			MRPS11		
Hs	3	NT_029289	Hs	5	NT_010274
Ol	3	scaffold3257	Ol	5	scaffold2637
Ci	3	scaffold_40	Ci	0	scaffold_59
Dm	1	NC_004354†	Dm	4	NT_033777
Ce	2	NC_003281	Ce	1	NC_003283
Sp	0	NC_003423†			
Dd	2	DDB0183952			
At	5	NC_003071†			
Pf	2	NC_004326			

Table S1. - *Continued*

RPS16			MRPS9		
Hs	4	NT_011109	Hs	10	NT_022171
Ol	4	scaffold3619	Ol	10	scaffold6077
Ci	3	scaffold_406	Ci	10	scaffold_2034, 648‡
Dm	4	NT_033778	Dm	6	NT_033777
Ce	1	NC_003283	Ce	8	NC_003281
Sp	1	NC_003424†			
Dd	1	DDB0185840			
At	0	NC_003071†			
Pf	1	NC_004329			
RPS20			MRPS10		
Hs	3	NT_008183	Hs	6	NT_007592
Ol	3	scaffold3867	Ol	4	scaffold2620
Ci	1	scaffold_11	Ci	0	scaffold_169
Dm	3	NT_033777	Dm	1	NT_033777
Ce	3	NC_003279	Ce	2	NC_003281
Sp	0	NC_003421			
Dd	0	DDB0205449			
At	2	NC_003074†			
Pf	1	NC_004314			
RPS23			MRPS12		
Hs	3	NT_006713	Hs	1	NT_011109
Ol	3	scaffold18137	Ol	1	scaffold611
Ci	3	scaffold_813	Ci	3	scaffold_4
Dm	2	NT_033778	Dm	1	NT_033777
Ce	2	NC_003282	Ce	3	NC_003283
Sp	0	NC_003423†			
Dd	4	DDB0203686			
At	3	NC_003074†			
Pf	2	NC_000521			
RPS29			MRPS14		
Hs	2	NT_026437	Hs	2	NT_004487
Ol	2	scaffold567	Ol	2	scaffold3906
Ci	2	scaffold_947	Ci	0	scaffold_2035
Dm	2	NT_033777	Dm	1	AE003512
Ce	2	NC_003281	Ce	3	NC_003280
Sp	2	NC_003423			
Dd	1	DDB0206545			
At	2	NC_003074†			
Pf	0	-			
RPL3			MRPL3		
Hs	9	NT_011520	Hs	9	NT_005612
Ol	8	scaffold9308	Ol	9	scaffold8715
Ci	7	scaffold_15	Ci	7	scaffold_217
Dm	5	NT_033777	Dm	3	NT_004354
Ce	3	NC_003281	Ce	4	NC_003281
Sp	0	NC_003424†			
Dd	2	DDB0184143			
At	4	NC_003070†			
Pf	0	NC_004314			
RPL5			MRPL18		
Hs	7	NT_032977	Hs	3	NT_007422
Ol	7	scaffold2455	Ol	3	scaffold6047
Ci	6	scaffold_308	Ci	3	scaffold_1263
Dm	3	AABU01002768	Dm	0	NT_033778
Ce	3	NC_003280	Ce	2	NC_003281
Sp	0	NC_003423†			
Dd	1	DDB0205521			
At	6	NC_003074†			
Pf	2	NC_004317			

Table S1. - *Continued*

RPL7			MRPL30		
Hs	5	NT_008183	Hs	4	NT_022171
Ol	5	scaffold8151	Ol	4	scaffold9427
Ci	5	scaffold_280	Ci	4	scaffold_240
Dm	1	NT_033779	Dm	1	NT_004354
Ce	4	NC_003279	Ce	4	NC_003281
Sp	1	NC_003424†			
Dd	2	DDB0217817			
At	5	AC006200†			
Pf	2	NC_000521			
RPL8			MRPL2		
Hs	4	NT_037704	Hs	6	NT_007592
Ol	4	scaffold8817	Ol	5	scaffold6789
Ci	4	scaffold_421	Ci	5	scaffold_13
Dm	1	NT_037436	Dm	1	NT_037436
Ce	2	NC_003283	Ce	5	NC_003282
Sp	0	NC_003423†			
Dd	1	DDB0167638			
At	1	NC_003071†			
Pf	1	NC_004326			
RPL10			MRPL16		
Hs	5	NT_025965	Hs	3	NT_033903
Ol	2	scaffold7299§	Ol	4	scaffold133
Ci	4	scaffold_124	Ci	0	scaffold_1002
Dm	3	NT_037436	Dm	2	NT_004354
Ce	2	NC_003280	Ce	3	NC_003281
Sp	0	NC_003424†			
Dd	3	DDB0219325			
At	3	NC_003070†			
Pf	0	NC_004317			
RPL10A			MRPL1		
Hs	5	NT_007592	Hs	7	NT_016354
Ol	5	scaffold3346	Ol	8	scaffold23388
Ci	5	scaffold_175	Ci	5	scaffold_326
Dm	2	NT_037436†	Dm	2	NT_033777
Ce	2	NC_003279	Ce	3	NC_003282
Sp	1	NC_003423†			
Dd	1	DDB0169032			
At	4	NC_003076†			
Pf	1	NC_004317			
RPL12			MRPL11		
Hs	6	NT_008470	Hs	4	NT_033903
Ol	5	scaffold2424	Ol	4	scaffold6616
Ci	5	scaffold_2639	Ci	1	scaffold_22
Dm	3	NT_033778	Dm	2	NT_033777
Ce	3	NC_003282	Ce	2	NC_003281
Sp	1	NC_003421†			
Dd	1	DDB0187878			
At	0	NC_003076†			
Pf	1	NC_004326			
RPL13A			MRPL13		
Hs	7	NT_011109	Hs	6	NT_031821
Ol	6	scaffold1092	Ol	6	scaffold658, 43808‡
Ci	5	scaffold_19	Ci	5	scaffold_155
Dm	2	NT_033777	Dm	2	NT_033779
Ce	2	NC_003281	Ce	3	NC_003279
Sp	1	NC_003423			
Dd	1	DDB0217704			
At	3	NC_003076†			
Pf	1	NC_004314			

Table S1. - *Continued*

RPL17			MRPL22		
Hs	5	NT_010966	Hs	6	NT_029289
Ol	5	scaffold15621	Ol	6	scaffold2018, 23552‡
Ci	2	scaffold_101	Ci	0	scaffold_281§
Dm	1	NC_004354	Dm	2	NT_004354
Ce	2	NC_003279	Ce	3	NC_003281
Sp	0	NC_003421†			
Dd	2	DDB0186425			
At	6	NC_003070†			
Pf	2	NC_004331			
RPL23			MRPL14		
Hs	4	NT_010755	Hs	1	NT_007592
Ol	4	scaffold467	Ol	1	scaffold4429
Ci	4	scaffold_229	Ci	0	scaffold_1310
Dm	2	NT_033778	Dm	0	NT_004354
Ce	1	NC_003281	Ce	2	NC_003280
Sp	1	NC_003421†			
Dd	3	DDB0188834			
At	3	NC_003074†			
Pf	1	NC_004331			
RPL23A			MRPL23		
Hs	4	NT_010799	Hs	4	NT_009237
Ol	4	scaffold2064	Ol	4	scaffold18271, 9183, 25073§,‡
Ci	3	scaffold_693	Ci	3	scaffold_419
Dm	2	NT_037436	Dm	1	NT_037436
Ce	2	NC_003284	Ce	1	NC_003279
Sp	1	NC_003423†			
Dd	2	DDB0191977			
At	2	NC_003074†			
Pf	3	NC_004331			
RPL26			MRPL24		
Hs	2	NT_010718	Hs	4	NT_009237
Ol	2	scaffold422, 2162‡	Ol	4	scaffold13904
Ci	2	scaffold_140	Ci	5	scaffold_164
Dm	0	NT_037436	Dm	3	NT_033779
Ce	2	NC_003280	Ce	6	NC_003279
Sp	0	NC_003423			
Dd	1	DDB0185662			
At	0	NC_003076†			
Pf	2	NC_000521			
RPL27A			MRPL15		
Hs	4	NT_009237	Hs	4	NT_008183
Ol	4	scaffold5189	Ol	4	scaffold516
Ci	4	scaffold_340	Ci	0	scaffold_62
Dm	2	NT_033779	Dm	1	NT_037436
Ce	2	NC_003279	Ce	2	NC_003279
Sp	0	NC_003421†			
Dd	1	DDB0184374			
At	0	NC_003070†			
Pf	1	NC_004327			
RPLP0			MRPL10		
Hs	6	NT_009775	Hs	4	NT_010783
Ol	0	-	Ol	4	scaffold292
Ci	6	scaffold_24	Ci	0	-
Dm	1	NT_037436	Dm	1	NT_033779
Ce	2	NC_003279	Ce	3	NC_003280
Sp	1	NC_003421			
Dd	0	DDB0218835			
At	4	NC_003074†			
Pf	0	NC_004315			

Table S1. - *Continued*

RPLP1			MRPL7/L12		
Hs	3	NT_010194	Hs	4	NT_024871
Ol	3	scaffold27636, 52950‡	Ol	4	scaffold8
Ci	3	scaffold_150	Ci	0	scaffold_21
Dm	1	NT_033779	Dm	0	NT_037436
Ce	1	NC_003279	Ce	2	NC_003281
Sp	2	NC_003423†			
Dd	1	DDB0205516			
At	2	NC_003070†			
Pf	1	NC_004315			

*For *O. laptides* and *Ci. intestinalis*, Scaffold number was shown. For *Di. discoideum*, dictyBaseID was shown.
†For genes with multiple copies, only one copy was used. When the number of introns in multiple copies of the same gene was different, the one with the most introns was used.
‡Gene structures were determined by a combination of these sequences.
§Gene sequences were partial.
At, *A. thaliana*; Ce, *Ca. elegans*; Ci, *Ci. intestinalis*; Dd, *Di. discoideum*; Dm, *Dr. melanogaster*; Hs, *H. sapiens*; Ol, *O. laptides*; Pf, *P. falciparum*; Sp, *S. pombe*.
